# Supplementary material for: Integrating sustainable Islamic social finance: An Analytical Network Process using the Benefit Opportunity Cost Risk (ANP BOCR) framework: The case of Indonesia
Source: PLoS One. 2022 May 26;17(5):e0269039. doi: 10.1371/journal.pone.0269039 (PMC9135187; doi:10.1371/journal.pone.0269039)
Supplement: S1 Appendix — (DOCX) [file pone.0269039.s001.docx]

**S1 Appendix. Questionnaire.**

*Integrating sustainable Islamic social finance: An Analytical Network Process using the Benefit Opportunity Cost Risk (ANP BOCR) framework: The case of Indonesia*

This questionnaire aims to develop solutions and strategies to build a sustainable Islamic Social Finance integration model by considering the benefits, opportunity costs and risks (BOCR) of the model built. This questionnaire aims to collect primary data in order to develop the Analytical Network Process model. It is hoped that in this study the strategy can be developed using the Analytical Network Process.

This questionnaire is the primary data collection stage for the research “Sustainable Islamic Social Finance Model: Strategic Solution in Countering Adverse Impacts of Social Economy of The Covid-19 Using Analytical Network Process Benefit Opportunities Cost Risk. This questionnaire is designed to determine the personal assessment of key informants, which include practitioners, experts/experts, government representatives, academics, community leaders, and associations. There is no right or wrong answer, but this assessment aims to compare the level of importance or priority of each attribute to a problem. The rating scale will be explained as follows:

1 = Not important or not influential

2-3= Less important or less influential

4-5= Important or influential

6-7= Very important or very influential

8-9= Absolutely important or absolutely influential

**SECTION 1: IMPORTANT ASPECT**

1. Looking at the following aspects, **how important are these aspects in the integration of Islamic Social Finance?**
2. Governance
3. Sustainable financing
4. Institutional
5. HR Islamic Social Finance Institute
6. Regulation
7. Use of Technology
8. In your opinion, from the four aspects below, **which aspect has the highest priority compared to all aspects**?
9. Benefit
10. Opportunity
11. Cost
12. Risk

**SECTION 2: ANALYSIS OF BENEFIT, OPPORTUNITY, COST, RISK (BOCR)**

**BENEFIT**

1. The following are several of **benefits from the governance aspect in supporting the creation of Islamic Social Finance integration**, how important are the benefits below in supporting the creation of Islamic Social Finance integration?
2. There is an integration of Islamic social finance into commercial enterprises
3. There is an MoU between BAZNAS and BWI to integrate zakat and waqf
4. Many previous studies support the governance of zakat and waqf
5. There are already standards for zakat and waqf management
6. Some programs increase the motivation of donors to channel funds to Islamic social, financial institutions
7. Every Islamic social finance institution has a supervisor so that the institution's operations run according to the plans and applicable rules
8. The existence of a sharia supervisory board makes decisions more appropriate and following sharia rules
9. Strengthening SDGs for Islamic social finance institutions will provide clear and measurable goals
10. There are financial reporting standards for Islamic social financial institutions
11. National data integration
12. Increased effectiveness and efficiency in the use of operational funds
13. The following are several **benefits from the aspect of sustainable financing in supporting the creation of the integration of Islamic Social Finance**; how important are the benefits below in supporting the creation of the integration of Islamic Social Finance?
14. The availability of cheap funds sourced from zakat, *infaq*, alms, waqf will make it easier for MSMEs to get financing
15. Financing carried out at BMT includes social and financial functions commercial to increase the competitiveness of BMT
16. There are many contracts in financing (*qard, ijarah, mudharabah*, etc.) that can reach a wider target recipient.
17. The following are several **benefits from the institutional aspect in supporting the creation of Islamic Social Finance integration**; how important are the benefits below in supporting the creation of Islamic Social Finance integration?
18. The broad reach of each Islamic social financial institution spread across Indonesia
19. The existence of branch offices or representative offices in various regions provides a broader range of benefits
20. The ZISWAF Fund managed by the institution becomes more organized
21. Islamic social finance institutions established by the community/social community have the potential to develop faster
22. The increasing interest of donors to pay ZISWAF in institutions
23. There is an association in Islamic social fund institutions
24. The following are several **benefits from the HR aspect in the Institution in supporting the creation of the integration of Islamic Social Finance**; how important are the benefits below in supporting the creation of the integration of Islamic Social Finance?
25. Human resource loyalty in Islamic social fund management institutions occurs because of the high level of religiosity
26. Increase professionalism of human resource
27. The following are several **benefits from the regulatory aspect in supporting the creation of Islamic Social Finance integration**; how important are the benefits below in supporting the creation of Islamic Social Finance integration?
28. ZISWAF rules in Indonesia are more complete compared to other countries
29. The regulations do not prohibit one institution from having a dual role, namely as a nadzir and amyl
30. There is political support from the government executive board for the optimization of ZISWAF
31. The following are several **benefits of using technology in supporting the creation of the integration of Islamic Social Finance**; how important are the benefits below in supporting the creation of the integration of Islamic Social Finance?
32. The use of technology makes it easier to raise and distribute funds
33. Availability of platforms for national data integration so that data becomes accurate
34. Access to information becomes faster and easier
35. Technology provides a wider range of socialization

**OPPORTUNITY**

1. The following are several **opportunities from the governance aspect in supporting the creation of Islamic Social Finance integration**; how important are the opportunities below in supporting the creation of Islamic Social Finance integration?
2. Achieved realization of the potential for collecting zakat and waqf in Indonesia
3. The collaboration of ZISWAF funds with BMT will present optimal Islamic social finance management
4. Improving the performance and effectiveness of Islamic social finance institutions
5. The emergence of a more comprehensive performance appraisal
6. The higher contribution of Islamic social finance in achieving the SDGs, especially the needs during the pandemic
7. Increasing the image of Islamic social financial institutions
8. Increased transparency of social financial management
9. Availability of accurate information for stakeholders
10. Increased public trust in Islamic social financial institutions
11. Increased capacity building
12. The ability of Islamic social financial institutions to carry out their duties and functions effectively and efficiently
13. The following are several **opportunities from the aspect of sustainable financing in supporting the creation of the integration of Islamic Social Finance**; how important are the opportunities below in supporting the integration of Islamic Social Finance?
14. Ease of accessibility to Islamic social financial institutions, both in urban and rural areas
15. The creation of a sustainable Islamic social finance ecosystem
16. The level of funding needs is high, especially in the productive sector.
17. Increased profit and income from financing
18. Increased productivity of both the recipient of the financing or the funds used
19. The decrease in the total poverty zone in Indonesia, which is in line with the SDGs
20. The following are several **opportunities from the institutional aspect in supporting the creation of Islamic Social Finance integration**; how important are the opportunities below in supporting the creation of Islamic Social Finance integration?
21. The diversity of Islamic social financial institutions further optimizes ZISWAF funds
22. The increasing number of Islamic social financial institutions spread to the lower levels
23. The existence of Islamic social financial institutions or services to the lowest level, for example: villages, etc.
24. The following are several **opportunities from the HR aspect in the Institution in supporting the creation of Islamic Social Finance integration**; how important are the opportunities below in supporting the creation of Islamic Social Finance integration?
25. Increasing the number of human resources who have competence in the management of Islamic social finance
26. Increasing the competitiveness of institutional HR
27. Availability of new job opportunities and increased absorption of labor
28. The following are a number of **opportunities from the regulatory aspect to support the creation of the integration of Islamic Social Finance**, how important are the opportunities below in supporting the creation of the integration of Islamic Social Finance?
29. There are opportunities for regulatory development if needed
30. Increased government support in innovative ZISWAF development and optimization of Islamic social finance
31. Improved relations between OIC countries related to the management of Islamic social finance
32. The following are several **opportunities from using technology to support the integration of Islamic Social Finance**; how important are the opportunities below in supporting the creation of the integration of Islamic Social Finance?
33. Increased public understanding of technology
34. Increasing the effectiveness and efficiency of Islamic social finance management

**COST**

1. The following are several **costs on the aspects of governance needed in the integration of Islamic Social Finance**, how much are the costs below in the integration of Islamic Social Finance?
2. There is a high-cost requirement to improve people's literacy
3. Islamic social finance management requires high costs, one of which is related to infrastructure development, provision of tools, operational costs to monitoring systems
4. The existence of the Sharia Supervisory Board (DPS) makes the bureaucratic flow take a long time
5. The high cost of conducting a public audit
6. Preparation of standardized reports takes quite a long time
7. The gap between academics and practitioners in the practice of managing Islamic social finance
8. Inefficient administration
9. The following are several **costs from the sustainable financing aspects needed in the integration of Islamic Social Finance**, how much are the costs below in the integration of Islamic Social Finance?
10. High service fees for Islamic financing
11. The position of Islamic social financial institutions is not strong, so they are very dependent on other intermediary institutions
12. Public perception that Islamic financing still contains usury
13. High marketing costs for strengthening Islamic financing literacy in Islamic social financial institutions.
14. The following are several **costs from the institutional aspects needed in the integration of Islamic Social Finance**, how much are the costs below in the integration of Islamic Social Finance?
15. Differences in the pattern of government-based and community/private-based institutions
16. Islamic social finance management through an institutional approach requires high costs
17. The following are several **costs from the HR aspect in institutions that are needed to integrate Islamic Social Finance**; how much are the costs below in the integration of Islamic Social Finance?
18. Training for HR requires a large amount of money
19. Mismatch in the background of the manager of Islamic social financial institutions
20. Low knowledge of human resources in the field of financial management
21. Limited HR mobility during the Pandemic
22. The following are several **costs from the regulatory aspect in the integration of Islamic Social Finance**, how much are the costs below in integrating Islamic Social Finance?
23. Limitations of guidance in the management of ZISWAF when the Act is issued
24. Differences in permits for Islamic social finance management institutions
25. Conflict handling costs
26. Conflict of interest between stakeholders
27. The integration of ZISWAF management takes a long time due to political factors
28. The following are several **costs from the aspect of using technology needed in the integration of Islamic Social Finance**, how much is the cost below in the integration of Islamic Social Finance?
29. Implementation of digital technology requires high costs
30. The use of technology will provide challenges related to the fulfillment of sharia principles

**RISK**

1. The following are several **risks from the governance aspect in integrating Islamic Social Finance**; how influential are the risks below in the integration of Islamic Social Finance?
2. There is mismanagement in the implementation of the functions of Islamic social financial institutions
3. Islamic social finance institutions have not adopted all the principles in governance
4. There is potential for miscommunication both internally and externally by Islamic social financial institutions
5. Mis-Coordination related to the division of tasks and roles
6. Differences in work patterns and culture between institutions
7. Incompatibilities of the supervisory board
8. Supervision is not carried out thoroughly
9. Reports that are not representative and less accurate
10. There is potential for non-compliance with regulations, both formal rules and sharia rules in Islamic social finance governance
11. The achievement of several SDGs points is contradictory to the essence of the function of Islamic social finance institutions
12. The 17 goals in the SDGs are not all relevant to the ZISWAF goals
13. The following are several **risks from the sustainable financing aspect in the integration of Islamic Social Finance**, how influential are the risks below in integrating Islamic Social Finance?
14. There is liquidity risk for financial institutions
15. The absence of guarantees creates a moral hazard problem
16. Lack of motivation of recipients of Islamic social finance to develop
17. Failure in carrying out financing
18. Time is needed in conditioning the synchronization between ziswaf institutions and the government regarding the legality of the law
19. Literacy related to Islamic social funds is still far from expected
20. High refusal to use waqf funds to lend
21. The following are several **risks from the institutional aspect in integrating Islamic Social Finance**; how influential are the risks below in integrating Islamic Social Finance?
22. The institutional model is not flexible in accommodating problems
23. Institutional fund management will limit the space for innovation.
24. The following are several **risks from the HR aspect in institutions in the integration of Islamic Social Finance**, how influential are the risks below in the integration of Islamic Social Finance?
25. High employee turnover rate
26. The profession of nazhir and/or amil is not considered as the primary profession
27. Increasing digitalization will replace the role and number of HR
28. The following are several **risks from regulatory aspects in integrating Islamic Social Finance**; how influential are the risks below in the integration of Islamic Social Finance?
29. There are no mandatory rules
30. There is a period for regulatory amendments, so regulations are less flexible in responding to current conditions
31. There are political challenges in strengthening regulations
32. Change of leader replaces policy direction
33. Conflicts of interest that arise between policymakers
34. The following are several **risks from using technology in the integration of Islamic Social Finance**; how influential are the risks below in the integration of Islamic Social Finance?
35. There is a crime in the implementation of technology
36. There is a potential for non-fulfillment of sharia principles with the use of technology

**SECTION 3: SOLUTION AND STRATEGIES IN INTEGRATING ISLAMIC SOCIAL FINANCE**

1. The following are several **solutions in integrating Islamic Social Finance**; how important are the solutions below in the integration of Islamic Social Finance?
2. Financial integration in providing funding to beneficiaries through the distribution of zakat, waqf, and Islamic microfinance based on the beneficiaries' economic circumstances and capabilities
3. Integrating ISF with state fiscal instruments, which is supported by existing regulations
4. Integration through non-formal coordination between ISF institutions
5. Integration among Islamic social finance institutions based on capacity building and role division
6. Establishing an integrated national data center for all ISF instruments
7. Integration based on regional clusters with a solid authority
8. Integrating ISF through the passage of new laws and regulations
9. Integration based on ISF management clusters, such as zakat *Amils* joining other zakat institutions and waqf manager joining other waqf institutions
10. The following are several **strategies in integrating Islamic Social Finance**; how important are the strategies below in the integration of Islamic Social Finance?
11. Improve the quality of human resources through training and certification
12. Promotes the availability of real-time data through database integration and reporting
13. Optimizing the digital technology in Islamic social finance instruments collection, management, and distribution process
14. Coordination and synergy among stakeholders (e.g., practitioners, regulators, associations, and academicians) to establish shared commitment and vision
15. Encouraging the broadening of the range of benefits by establishing service units at lower levels, such as service units at the district, village, and mosque levels, as well as strategic locations that support the broadening of the reach of the benefits of ISF instruments
16. Establishing a new institution that is capable of integrating all instruments of ISF
17. Realizing the government's role in supporting the integration of ISF through regulation and socialization
18. Developing the ISF institutions to have two functions, namely, *amil* and *nazir*
19. Revising and updating all laws and regulations governing the management of Islamic social finance instruments to accommodate the development of ISF instruments
