# Supplementary material for: Integrating sustainable Islamic social finance: An Analytical Network Process using the Benefit Opportunity Cost Risk (ANP BOCR) framework: The case of Indonesia
Source: PLoS One. 2022 May 26;17(5):e0269039. doi: 10.1371/journal.pone.0269039 (PMC9135187; doi:10.1371/journal.pone.0269039)
Supplement: Attachment — Submitted filename: PONE-D-21-31476_Re_review.pdf [file pone.0269039.s004.pdf]

PLOS ONE

Integrating Sustainable Islamic Social Finance: Analytical Network Process using Benefit Opportunity Cost Risk (ANP BOCR) Framework: The Case of Indonesia

--Manuscript Draft--

**Manuscript Number:**	PONE-D-21-31476R1
**Article Type:**	Research Article
**Full Title:**	Integrating Sustainable Islamic Social Finance: Analytical Network Process using Benefit Opportunity Cost Risk (ANP BOCR) Framework: The Case of Indonesia
**Short Title:**	Integrating Sustainable Islamic Social Finance using ANP BOCR Framework
**Corresponding Author:**	Anidah Robani<br>Universiti Teknikal Malaysia Melaka<br>Durian Tunggal, Melaka MALAYSIA
**Keywords:**	Islamic Social Finance, Financial Sustainability, Integration, Islamic Solidarity Economy, Analytical Network Process, BOCR
**Abstract:**	The utilization of Islamic social finance instruments is far behind what is expected. To realize its full potential, Islamic social finance instruments must be integrated. This study examined solutions and priority strategies for integrating sustainable Islamic social finance that could be implemented in the short and long term using the Benefit, Opportunity, Cost, and Risk (BOCR) framework, which includes six aspects: Governance, sustainable financing, institutional aspect, human resources, regulations, and supporting technology. This qualitative research employed the Analytic Network Process (ANP) method using the benefit, opportunity, cost, and risk analysis. The data were obtained mainly through focus group discussions and in-depth interviews with respondents among academics, practitioners, associations, regulators, and community leaders. The respondents were selected for their expertise and experience in the selected topic. The data were processed using the Microsoft Excel and Super Decision software. There are several findings worth considering from the analysis. First, the highest priority in integrating Islamic social finance aspects are human resources (0.97), regulation (0.86), and technology (0.76). Second, based on the short- and long-term analysis, financial integration through sustainable financing (0.01 and 1.44, respectively) and improving human resource quality through certification and training (0.01 and 1.56, respectively) is a priority solution and strategy to integrate sustainable Islamic social finance. Meanwhile, according to expert judgments, integrating national data (0.24) and optimizing technology use (0.18) are priority solutions and strategies. The findings emphasize the critical role of improving human resource quality to utilize technology, with experts identifying a national data integration as the most critical solution. As a result, relevant stakeholders are concerned about technology management training for Islamic philanthropic managers, with the goal of maximizing the potential of technology's growing role and adoption.
**Order of Authors:**	Tika Widiastuti
	Anidah Robani
	Puji Sucia Sukmaningrum
	Imron Mawardi
	Sri Ningsih
	Sri Herianingrum
	Muhammad Ubaidillah Al-Mustofa
**Response to Reviewers:**	We had made the required amendments and changes required by the academic editor and reviewers in the cover letter and in a rebuttal letter labeled 'Response to Reviewers' as attachment. Kindly refer to the attachment files at the main page.
**Additional Information:**	
**Question**	**Response**

Powered by Editorial Manager® and ProduXion Manager® from Aries Systems Corporation

**Financial Disclosure**<br><br>Enter a financial disclosure statement that describes the sources of funding for the work included in this submission. Review the submission guidelines for detailed requirements. View published research articles from *PLOS ONE* for specific examples.<br><br>This statement is required for submission and **will appear in the published article** if the submission is accepted. Please make sure it is accurate.<br><br>**Unfunded studies**<br>Enter: *The author(s) received no specific funding for this work.*<br><br>**Funded studies**<br>Enter a statement with the following details:<br>• Initials of the authors who received each award<br>• Grant numbers awarded to each author<br>• The full name of each funder<br>• URL of each funder website<br>• Did the sponsors or funders play any role in the study design, data collection and analysis, decision to publish, or preparation of the manuscript?<br>• **NO** - Include this sentence at the end of your statement: *The funders had no role in study design, data collection and analysis, decision to publish, or preparation of the manuscript.*<br>• **YES** - Specify the role(s) played.<br><br>* typeset	This research grant is a Covid-19 Research Mandate with the contract number 1025/UN3.15/PT/2021. The authors would like to thank the Research and Community Service Institution (LPPM) of Universitas Airlangga for their financial assistance. The authors would also like to thank all academicians, regulators, associations, practitioners, and community leaders for their support in writing this research. The funders had no role in study design, data collection and analysis, decision to publish, or preparation of the manuscript.
**Competing Interests**<br><br>Use the instructions below to enter a competing interest statement for this submission. On behalf of all authors, disclose any competing interests that could be perceived to bias this work—acknowledging all financial support and any other relevant financial or non-financial competing interests.<br><br>This statement is required for submission	he authors have declared that no competing interests exist.

and **will appear in the published article** if the submission is accepted. Please make sure it is accurate and that any funding sources listed in your Funding Information later in the submission form are also declared in your Financial Disclosure statement.<br><br>View published research articles from *PLOS ONE* for specific examples.<br><br>**NO authors have competing interests**<br><br>Enter: *The authors have declared that no competing interests exist.*<br><br>**Authors with competing interests**<br><br>Enter competing interest details beginning with this statement:<br><br>*I have read the journal's policy and the authors of this manuscript have the following competing interests: [insert competing interests here]*<br><br>* typeset	
**Ethics Statement**<br><br>Enter an ethics statement for this submission. This statement is required if the study involved:<br><br>• Human participants<br>• Human specimens or tissue<br>• Vertebrate animals or cephalopods<br>• Vertebrate embryos or tissues<br>• Field research<br><br>Write "N/A" if the submission does not require an ethics statement.<br><br>General guidance is provided below. Consult the submission guidelines for detailed instructions. **Make sure that all information entered here is included in the Methods section of the manuscript.**	Informed consent for participation was obtained from respondents who participated in the survey. The respondents who participated in the survey were asked to read the ethical statement (there is no compensation for responding nor is there any known risk. To ensure that all information will remain confidential, please do not include your name. Participation is strictly voluntary and you may refuse to participate at any time) and proceed only if they agree. No data was collected from anyone under 16 years old.<br><br>Human Subject Research which involves human participants. Research and Community Service Institution (LPPM) of Universitas Airlangga for their ethical consent and approval for this research. They could be contacted at penelitian@feb.unair.ac.id

Powered by Editorial Manager® and ProduXion Manager® from Aries Systems Corporation

**Format for specific study types**<br><br>**Human Subject Research (involving human participants and/or tissue)**<br>• Give the name of the institutional review board or ethics committee that approved the study<br>• Include the approval number and/or a statement indicating approval of this research<br>• Indicate the form of consent obtained (written/oral) or the reason that consent was not obtained (e.g. the data were analyzed anonymously)<br><br>**Animal Research (involving vertebrate animals, embryos or tissues)**<br>• Provide the name of the Institutional Animal Care and Use Committee (IACUC) or other relevant ethics board that reviewed the study protocol, and indicate whether they approved this research or granted a formal waiver of ethical approval<br>• Include an approval number if one was obtained<br>• If the study involved *non-human primates*, add *additional details* about animal welfare and steps taken to ameliorate suffering<br>• If anesthesia, euthanasia, or any kind of animal sacrifice is part of the study, include briefly which substances and/or methods were applied<br><br>**Field Research**<br><br>Include the following details if this study involves the collection of plant, animal, or other materials from a natural setting:<br>• Field permit number<br>• Name of the institution or relevant body that granted permission	
**Data Availability**<br><br>Authors are required to make all data underlying the findings described fully available, without restriction, and from the time of publication. PLOS allows rare exceptions to address legal and ethical concerns. See the PLOS Data Policy and FAQ for detailed information.	No - some restrictions will apply

A Data Availability Statement describing where the data can be found is required at submission. Your answers to this question constitute the Data Availability Statement and **will be published in the article**, if accepted.<br><br>**Important:** Stating 'data available on request from the author' is not sufficient. If your data are only available upon request, select 'No' for the first question and explain your exceptional situation in the text box.<br><br>Do the authors confirm that all data underlying the findings described in their manuscript are fully available without restriction?	
**Describe where the data may be found in full sentences. If you are copying our sample text, replace any instances of XXX with the appropriate details.**<br><br>• If the data are **held or will be held in a public repository**, include URLs, accession numbers or DOIs. If this information will only be available after acceptance, indicate this by ticking the box below. For example: *All XXX files are available from the XXX database (accession number(s) XXX, XXX.*).<br>• If the data are all contained **within the manuscript and/or Supporting Information files**, enter the following: *All relevant data are within the manuscript and its Supporting Information files.*<br>• If neither of these applies but you are able to provide **details of access elsewhere**, with or without limitations, please do so. For example:<br><br>*Data cannot be shared publicly because of [XXX]. Data are available from the XXX Institutional Data Access / Ethics Committee (contact via XXX) for researchers who meet the criteria for access to confidential data.*<br><br>*The data underlying the results presented in the study are available from (include the name of the third party*	Data cannot be shared publicly because of data integrity and security. Data are available from the Universitas Airlangga Institutional Data Access / Ethics Committee (contact via email ekis@feb.unair.ac.id) for researchers who meet the criteria for access to confidential data.

and contact information or URL).<br>• This text is appropriate if the data are owned by a third party and authors do not have permission to share the data.<br><br>* typeset	
Additional data availability information:	Tick here if your circumstances are not covered by the questions above and you need the journal's help to make your data available.

Manuscript Click here to access/download;Manuscript;Manuscript.docx

Integrating Sustainable Islamic Social Finance: An Analytical Network Process using the Benefit Opportunity Cost Risk (ANP BOCR) Framework: The Case of Indonesia

Tika Widiastuti[1], Anidah Robani[2*], Puji Sucia Sukmaningrum[3], Imron Mawardi[4], Sri Ningsih[5], Sri Herianingrum[6], Muhammad Ubaidillah Al-Mustofa[7]

[1,3,4,6,7]Department of Islamic Economic, Faculty of Economic and Business, Universitas Airlangga
[2]Institute of Technology Management and Entrepreneurship, Universiti Teknikal Malaysia Melaka (UTeM)
[5]Department of Accounting, Faculty of Economic and Business, Universitas Airlangga

*Corresponding author
Email: anidah@utem.edu.my

Abstract

The utilization of Islamic social finance instruments is far behind what is expected. To realize its full potential, Islamic social finance instruments must be integrated. This study examined solutions and priority strategies for integrating sustainable Islamic social finance that could be implemented in the short and long term using the Benefit, Opportunity, Cost, and Risk (BOCR) framework, which includes six aspects: Governance, sustainable financing, institutional aspect, human resources, regulations, and supporting technology. This qualitative research employed the Analytic Network Process (ANP) method using the benefit, opportunity, cost, and risk analysis. The data were obtained mainly through focus group discussions and in-depth interviews with respondents among academics, practitioners, associations, regulators, and community leaders. The respondents were selected for their expertise and experience in the selected topic. The data were processed using the Microsoft Excel and Super Decision software. There are several findings worth considering from the analysis. First, the highest priority in integrating Islamic social finance aspects are human resources (0.97), regulation (0.86), and technology (0.76). Second, based on the short- and long-term analysis, financial integration through sustainable financing (0.01 and 1.44, respectively) and improving human resource quality through certification and training (0.01 and 1.56, respectively) is a priority solution and strategy to integrate sustainable Islamic social finance. Meanwhile, according to expert judgments, integrating national data (0.24) and optimizing technology use (0.18) are priority solutions and strategies. The findings emphasize the critical role of improving human resource quality to utilize technology, with experts identifying a national data integration as the most critical solution. As a result, relevant stakeholders are concerned about technology management training for Islamic philanthropic managers, with the goal of maximizing the potential of technology's growing role and adoption.

Keywords: Integration, Sustainable Islamic Social Finance, ANP BOCR, Priority Strategy

Introduction

Islamic Social finance (ISF), which includes zakat, *infaq*, *sadaqah*, waqf, and Islamic microfinance, is critical to promoting a long-term economic development [1–3]. Islamic social finance has a vital role in solving the problem of poverty [3-6] , income inequality [7], global education [8,9], health access [10], and various other socio-economic problems. The role of Islamic social finance instruments is not only in the majority Muslim countries, but also in minority Muslim countries, such as the role of zakat in providing supportive programs for the poor in Singapore [11] , the role of Islamic social finance to support economic resilience in the UK [12], and the role of zakat in creating prosperity in Germany, France, and Croatia [13].

Islamic social finance has also shown a great promise in addressing socioeconomic issues during the Covid-19 pandemic. The pandemic has had a significant impact, especially in the economic field [14]. The economic implications of Covid-19, particularly in Indonesia [15,16], include increasing unemployment and underemployment rates, declining income levels, economic contraction, slow and uneven recovery process across sectors, including the health, economic, and education sectors. This is due to the limited sources of funding owned by the state. The government has prepared a reserve budget of USD 5.3 billion and a budget for the National Economic Recovery program of USD 37.3 billion. The funding was used for the health sector (USD 1.0 billion), social protection (USD 2.5 billion), and support for micro, small, and medium enterprises (USD 1.8 billion) [17]. The sources of funds were derived from debt, government bonds, Sukuk, and foreign debt. The Ministry of Finance [18] released the funding components of Covid-19 originating from market financing, bilateral and multilateral debts, private placement, financing from over budget balance (SAL), and the use of government endowment funds. These components certainly have limits to meet the growing needs of handling Covid-19. Consequently, the government needs to find alternative sources of funding. Islamic social finance with a great potential can be utilized to lessen the burden of funding and accelerate the recovery from the impact of the Covid-19 pandemic.

Indonesia, as a Muslim majority country, has a high potential for Islamic social finance. The potential for collecting zakat funds in Indonesia in 2020 was USD 23 billion [19], cash waqf was USD 12.6 billion per year, and the overall value of waqf land is USD 140 billion [18]. However, the collection of zakat and waqf funds is still far from its full potential. The zakat collection in Indonesia in 2019 was only USD 0.7 billion [19], while the cash waqf funds collected were only USD 0.2 billion [18]. One of the reasons for the vast gap between the realization and potential of ISF is a lack of optimal governance [20].

Furthermore, the management of ISF faces several problems, including the lack of technology utilization in its management [20], the lack of ability and competence of managers, and the lack of synergy between stakeholders [20,21], and lack of trust and public literacy [20,22]. Based on a survey conducted by BAZNAS (Zakat Government Institution), nationally, the level of zakat literacy is at 66.78 %, which is in the middle category. Despite the country having the largest Muslim population, the literacy level for several provinces in Indonesia is still in the low category, with a score below 50 %. This means that literacy is not at an advanced

stage, of which this includes knowledge about the existence and functions of ISF institutions, regulations, impacts, programs, and digital payments [23].

The governance of ISF includes aspects of collection, management, distribution, and utilization of social funds. The issues of ISF governance can be categorized into six aspects consisting of Human Resources (HR), Unsupported Policies and Regulations, Technology, Sustainable Financing, Governance, and Institutional Aspect [20,24–26]. Problems in the HR aspect include the lack of ability and motivation of human resources to develop both managers and recipients of Islamic social funds [20]. In addition, Islamic philanthropic institutions are dominated by employees who are not from the background of Islamic Economics and Zakat Waqf management, causing the low productivity to manage ZISWAF. In addition, the low literacy related to Islamic social funds needs to be addressed by policymakers [27].

Unsupported policies and regulations become issues from the regulatory aspect [20]. First, zakat is still a voluntary act [20] and there is no sanction for those who do not carry out zakat [28]. Further, the laws that govern ISF are separated as zakat is governed by Law No. 23 of 2011, whereas waqf is governed by Law No. 41 of 2004.

In addition, the infrastructure required for management needed to be addressed, particularly in the field of technology [20]. This becomes an issue that deserves consideration [29]. Many institutions do not have the required financial capacity to purchase the infrastructure needed to support technology-based governance.

Problems related to the sustainability aspects of the ISF program are of concern to IRTI [29], Pitchay et al. [24], and Sukmana [26]. Based on the findings in the field, most empowerment programs using Islamic social funds do not consider sustainability aspects and seem to end when the program ends, so that the primary goal of empowerment, namely, the transformation of *mustahiq* into *muzakki*, or *mauquf ' alaihi* into *wakif*, cannot be achieved. In addition, in the aspect of governance, Islamic social fund instruments are still partially managed. These instruments have not been integrated with the State's fiscal policy [30]. This is because Indonesia is based on "Pancasila", not a country based on Islamic law, of which its population consists of various beliefs and religions. There could be a sense of injustice if the government only regulates ands takes money from Muslims to help government finances. On the institutional aspect, there is a lack of coordination and synergy between stakeholders [20]. The lack of public trust in Islamic social finance managers [25] needs to be an essential concern for policymakers. To overcome these problems, the solution that can be done is to formulate the integration of the Islamic social finance sector and establish a sustainable management ecosystem.

The purpose of this research was to develop a strategy for the short- and long-term integration of ISF and to determine the best way to achieve that goal. This study was carried out in Indonesia, a country with a high potential for accumulating ISF . In practice, however, the ISF are only partially managed and have not been fully integrated. Raimi et al. [31], Jouti [32], and Shalleh et al. [33] conducted research on ISF integration. Previous research

formulated the integration of ISF without emphasizing sustainability, and thus, the sustainability aspect in integrating Islamic social funds is highlighted in this research. Furthermore, previous studies employed a qualitative approach through case studies, literature reviews, content and thematic analysis, and a quantitative approach through Structural Equation Modeling (SEM). There has been no research that incorporates ISF that employ the two-level ANP method. The analysis of Benefit, Opportunity, Cost, and Risk (BOCR) to build the ANP framework is what makes this research unique. Several factors influenced the decision to use this method. To begin, the ANP method was used to determine the best integration in the long-term management of ISF. Second, the analysis of the benefits, opportunities, costs, and risks of each aspect was to produce findings that can be implemented in the short and long term. Third, this study identified priority solutions and strategies for achieving long-term ISF integration using the ANP BOCR method.

The findings show that the financing integration model is a priority solution in the short and long term. This demonstrates that the ISF serves several functions: Consumptive zakat is used to provide emergency assistance in the short term, productive zakat and benevolence funds are used to support economic recovery in the medium term, and waqf or sukuk are used to build long-term resilience. Furthermore, the findings of this study highlight the importance of improving the quality of HR managers through a technology-based certification and training (particularly for data analysis, e-marketing and big database management) as the best short- and long-term strategy for implementing Islamic social finance in a sustainable manner.

The findings of this study are critical for several reasons. First, solutions and strategies for the integration of sustainable ISF are expected to solve various problems of managing different instruments of ISF, resulting in the formation of a sustainable ISF ecosystem. Second, the findings of this study serve as a resource for the ISF institutions in developing long-term strategic plans. Third, the results can provide a broader impact on people's well-being. Fourth, in the context of the Covid-19 pandemic, the findings can provide an overview of strategic policy steps and guidelines for seeking alternative funding sources by leveraging the potential of sustainable ISF.

Literature review

The concept of Islamic social finance and its instruments

Islamic social finance is a financial system that includes traditional and contemporary ISF instruments [32,34–36]. Traditional ISF can be divided into two types: Philanthropy and cooperation. Zakat, *infaq*, *sadaqah*, and waqf are traditional ISF instruments based on philanthropy, while *qard* and *kafalah* are based on cooperation. Furthermore, Islamic microfinance can be categorized as contemporary ISF. Islamic social finance contributes to the third sector of the economy by assisting in creating more economic activities and reducing unemployment and poverty [37]. Several previous studies have revealed the differences in the instruments used in Islamic social finance as shown in Table 1.

Table 1. Instruments of Islamic Social Finance

No	Researchers	Year	Zakat	*Infaq*	*Sadaqah*	Waqf	*Qard*	*Qard* Hasan	*Kafalah*	Gift	Islamic Microfinance
1.	Jouti	2019	✓		✓	✓	✓		✓		✓
2.	Usman et al.	2019	✓	✓	✓						
3.	Adjar et al.	2020	✓			✓		✓			
4.	Hudaefi	2020		✓	✓	✓					

Source: Authors' Compilation (2021)

Most of the previous research mentions that the instruments for ISF include zakat, *infaq*, *sadaqah*, and waqf [32,38,39]. Therefore, in this study, ISFinstruments refer to zakat, *infaq*, al and waqf, and Islamic microfinance.

Each of these instruments has different roles and functions but shares the same objective in achieving prosperity [32]. The first instrument is zakat, a mandatory economic instrument for every Muslim [40,41]. Zakat serves as a means for distributing wealth in the society [42] from the rich to the poor to achieve socio-economic justice, increase economic growth, and reduce inequality [43]. The second instrument is *infaq* and *sadaqah*, which are the voluntary gifts by capable people to seek the pleasure of Allah [44], significantly to help the needy. Zakat, *infaq*, and *sadaqah* are economic instruments that support economic growth by increasing the purchasing power of the needy [3,31].

As a third instrument, waqf is a social fund that plays a vital role in the Islamic framework. An asset owner donates and dedicates movable or immovable assets for the benefit of the community in perpetuity or impermanence. Waqf can be used to provide a variety of free community services, such as the construction of mosques, hospitals and healthcare centers, *madrasas*, education centers, and libraries [26]. Waqf is a vital tool in the Islamic economic system used to meet the needs of the poorest communities and promote community economic growth [26]. Furthermore, waqf, zakat, and sadaqah are instruments capable of acting as the national income and transfer payments used for redistributive purposes in an Islamic economy [6].

Islamic microfinance is the fourth instrument of ISF. Islamic microfinance serves two purposes: Social and commercial. As a social institution, it manages zakat, *infaq*, *sadaqah*, and waqf in accordance with the Syariah. On the other hand, as a commercial institution, it conducts businesses and provides financing for economic development. Islamic microfinance focuses on economic development through capital assistance and the empowerment of the marginal community with no access to capital to improve their well-being [45].

Sustainability of Islamic social finance

Sustainability refers to the capacity to operate over a long period and continuously [46]. Sustainable growth has become a global development goal in both government and non-governmental organizations [47]. There are two paradigms in sustainability, namely, the institutionalist and welfarist [48]. The institutionalist paradigm considers that financial self-

sufficiency is the key to achieving sustainability. Thus, a person can be said to be sustainable if he or she achieves financial independence [48]. In contrast, the welfarist paradigm considers that social functions in poverty alleviation are more important in achieving sustainability. Both paradigms are essential in the context of ISF. Financial self-sufficiency refers to an institution's ability to fund its operations independently, while also benefiting others. On the other hand, the main goals of ISF are poverty alleviation and its reachability [49–51]. Three factors contribute to the sustainability of Islamic social finance. First, the collection's sustainability is linked to the fact that funds will always be collected from the community. To ensure the long-term viability of funds supply, all information about zakat and its administration must be transparent and easily accessible to the public [52]. Second, institutional sustainability is linked to the institution's ability and availability to carry out operational tasks [48]. Third, the sustainability of distribution is linked to how the Islamic social finance institution performs its social and intermediary functions to improve welfare and achieve broader benefits [50].

In practice, the empowerment programs of ISF institutions have not been able to transform the poor into prosperous. Furthermore, many of these programs come to an end after a certain period and do not track whether the poor have truly become self-sufficient and prosperous. Several factors contribute to the unsustainable nature of ISF institutions' empowerment projects [20]. To begin, massive sums of money are required to provide effective and optimal empowerment. Second, qualified human resources with leadership qualities are needed [53]. Third, the beneficiary's desire and ability to develop and change into *muzakki* or *munfiq* are required [3,54].

Issues with Islamic social finance integration

In order to achieve optimal management, ISF must overcome several challenges. A lack of motivation among beneficiaries to develop, a lack of synergy and coordination among stakeholders, a lack of human resources capabilities and quantity to manages Islamic social finance, a lack of ability to optimize technology, regulations that are still voluntary, and a lack of awareness and trust of *muzakki* in Islamic social finance institutions are among the problems encountered by Islamic social finance [20,24–26,55]. Further, the ISF instruments are still partially (separatedly) managed, which is demonstrated by several points. First, according to the 2017-2022 Indonesian Zakat Architecture, zakat management is supported by regulations but remains a voluntary instrument, not an obligatory instrument [20]. Second, ISF instruments are governed by different regulations and laws. The management of zakat is regulated in Law no. 23 of 2011, while the management of waqf is regulated in Law no. 41 of 2004. Further, ISF instruments are managed by various institutions (government, corporate and community organizations) [56] of which there exist differences in the rights, obligations, and interests of different institutions. Fourth, the regulators of zakat and waqf are not the same. The National *Amil* Zakat Agency (BAZNAS) regulates zakat, while the Indonesian Waqf Board (BWI) is the waqf management regulator. However, BAZNAS and BWI are eventually accountable to the Minister of Religion Affairs. Based on these challenges, the integration of ISF must be carried out.

Integration model of Islamic social finance

Integration refers to the process of blending into a single unit, thus, integration in ISF combines funds management, institutions, governance, functions, etc. According to Balassa [57], integration offers various advantages. First, integration allows for the mobility of production elements, which has a positive impact on enhancing welfare. Second, integration reduces costs, resulting in an increase in output. Third, integration promotes job specialization. Fourth, integration creates a welfare through policy harmonization. Fifth, integration can increase technology adoption. Furthermore, integration has a favorable impact on the socio-economic aspects of the community, including: (1) Improving sustainability performance [58], (2) enhancing economic growth [59], and (3) improving socio-economic fairness [2].

Raimi et al. [31] integrated corporate social responsibility, zakat, and waqf into the faith-based model to reduce poverty, promote business development, and strengthen the economy of Muslim-majority communities. Hassanain [60] conducted a literature review on microfinance, waqf, and zakat to develop an integration model. Haneef et al. [45] used the Structural Equation Model (SEM) analysis to examine the significant influence between differencct aspects of Islamic social finance that include waqf, takaful, human resource management, and financing. Pitchay et al. [24] conducted a literature review to develop a cooperative-waqf model. Further, using the thematic analysis, Ambrose et al. [61] developed a waqf financing model for public goods and mixed public goods in Malaysia, where the proceeds of waqf investment can be used to fund 11 items of government spending.

A research that specifically discusses the sustainability of the ISF model was carried out by Jouti [32], which focuses on creating an ISF ecosystem, and by Shalleh et al. [33] which focuses on the integration of waqf-based takaful for flood victims in Malaysia. This study developed the previous research by building an integration model between ISF instruments with an emphasis on sustainability aspects using the two-level ANP method.

Method

Research method and analysis approach

The Analytical Network Process (ANP) method was used in this qualitative study, along with the analysis of Benefit, Opportunity, Cost, and Risk (BOCR). This two-level ANP method has the characteristics of mathematically examined conditions and inputs obtained based on several constructs, allowing it to aid the decision-making from various options and complex data [62]. The ANP method approach was chosen because ISF include various instruments, each with its own set of characteristics and issues, both tangible and intangible. In this study, the ANP approach was the best choice for producing strategic decisions [63]. Meanwhile, the BOCR analysis was selected for a variety of reasons, to begin, every decision has both positive and negative consequences, which must be considered when determining priority decisions. Second, the BOCR approach was used to make decisions that can be implemented in the short and long terms. The ANP BOCR method prioritizes solutions and strategies to develop a sustainable ISF integration model. The ANP method of research has three phases, which are depicted in Fig 1 [64].

Figure 1. ANP BOCR Research Stage

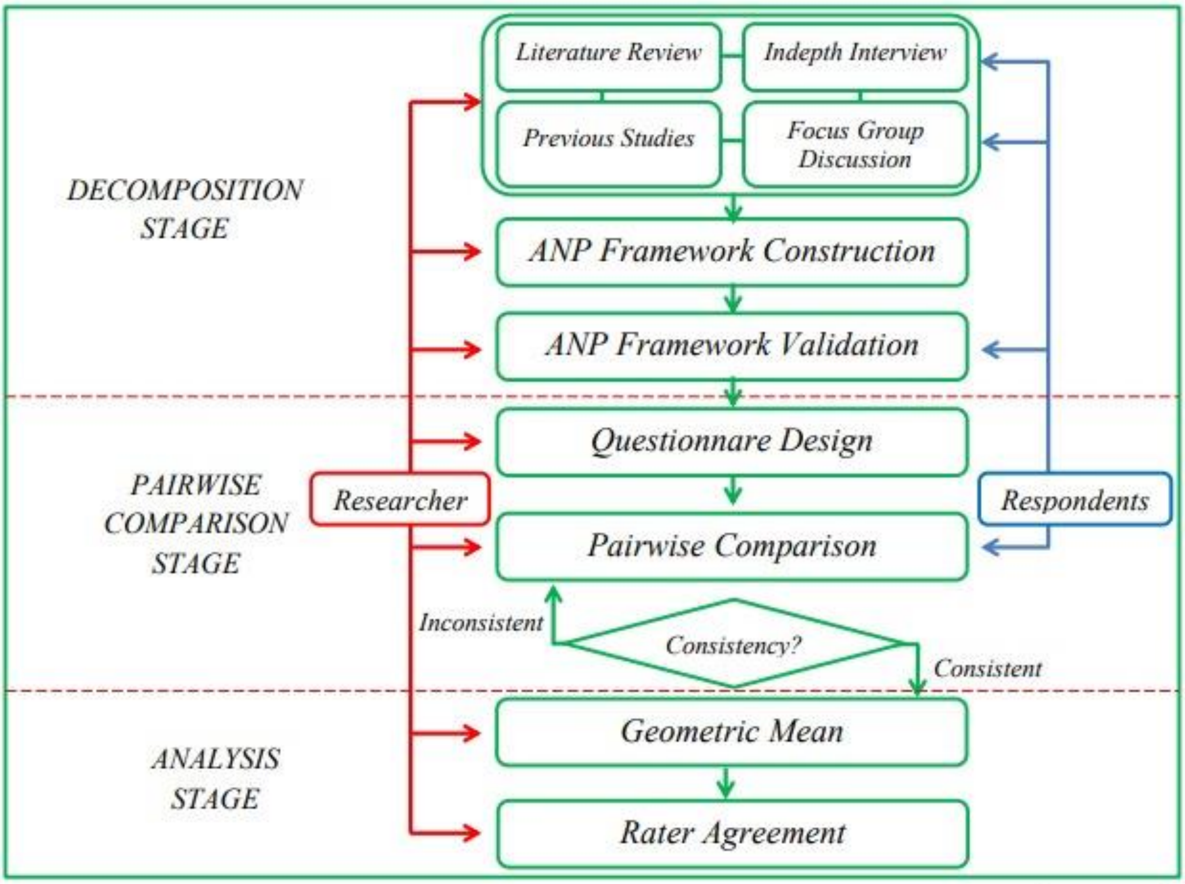

Source: Ascarya [64]

First, the decomposition phase sought to construct and validate the ANP model through focus group discussions and in-depth interviews with experts in ISF among academics, practitioners, associations, regulators, and community leaders. A literature review was conducted as part of this study to strengthen the ANP model that was developed. A literature review analysis was used to collect problems, solutions, and strategies discovered and analyzed by previous researchers to integrate sustainable ISF. In addition, the literature review analysis was used to validate the findings from the focus groups discussion and in-depth interviews.

Three Focus Group Discussions (FGDs) were held. The first and second FGDs were held with experts to identify aspects, solutions, and strategies for integrating sustainable ISF and identify benefits, opportunities, costs, and risks to determine the short-term and long-term analyses. The model developed through FGD 1 and 2 was then strengthened through in-depth interviews and literature reviews. Following that, the third FGD validated the constructed ANP model. The second was the pairwise comparison phase to design a questionnaire in the form of a pairwise comparison to determine and analyze aspects, solutions, and priority strategies in integrating sustainable ISF. The questionnaire that the respondents had answered were used for the next phase. The third was the data analysis phase, which aimed to calculate the priority value of each aspect, solution, and strategy formulated using the Microsoft Excel and Super

Decision software. The use of the ANP method with the BOCR approach made it possible to produce realistic and net values. The net value/additive negative describes alternative options that are more profitable or ideal to be prioritized in the long term, while realistic/multiplicative value represents more favorable outcomes for short-term decisions [63,65]. According to Saaty and Vargas [63] there are several steps to synthesize each priority option from each criterion where:

$$\text{Net Value/additive negative} = bB+oO-cC-rR \tag{1}$$

$$\text{Realistic/multiplicative} = (BxO)/(CxR) \tag{2}$$

B or b stands for Benefit, O or o stand for Opportunity, C or c stands for cost, R or r stands for Risk. After the analysis phase, the next step was to validate the results with various ISF experts to strengthen the integration model that will be built.

Data and research samples

The data used were primary data obtained through focus groups, in-depth interviews, and ANP questionnaires filled out by the respondents. The data sources gathered were from 13 experts among academics, practitioners, associations, regulators, and community leaders. In ANP research, the number of respondents is not a critical requirement for research validation [66] but their expertise is. Each expert completed the questionnaire consciously and in a mentally healthy state. The participants were required to fill in a specific consent form beforehand to ensure that they were not subject to any pressure from any party to participate in the interviews. The research team guaranteed the confidentiality of the experts' data and responses and is fully responsible for all applicable laws. Applicable laws will account for the disclosure of confidential data and experts' responses.

In terms of research ethics, this research followed the rules and ethics of research conduct and writing coordinated by the Institute for Research and Community Service (LPPM), Universitas Airlangga and those mandated by law. It is also explained in the Airlangga University Chancellor's Regulation Number 34 of 2019 concerning the Rules of Conduct clause 16 (b) that researchers must be honest, objective, and adhere to all aspects of the research process and may not falsify or manipulate data or research results, as well as clause 16 (f) that researchers must respect and appreciate the object of research, whether in the form of humans or animals, both living and dead, or parts/fragments of the object of research. In addition, this research was supervised by the Center for Research and Publications (3P) within the Faculty of Economics and Business Universitas Airlangga, which functions as an ethics committee according to the Dean Decree Number 88/UN3.1.4/2020.

The purposive sampling technique aims to select respondents according to the criteria required in the ANP method. For this study, the selected respondents must have the following

criteria: (1) Respondents are experts in the field of ISF among academics, practitioners, associations, regulators, and community leaders, (2) have more than two journal publications in the field of ISF for academic representatives, and (3) for representatives of regulator, practitioners, and associations, they should hold an important position and bear a great responsibility in the management of ISF in their respective institutions. The research team contacted the prospective (representative) respondents by phone and email before conducting the interviews to inquire about their willingness to complete the questionnaire. There were two options for completing the questionnaire: Online or offline. For the respondents who opted to complete the questionnaire offline, the research team attended to them, while adhering to a strict health protocol, by still maintaining a safe distance and wearing a mask. For respondents who opted to fill out questionnaires via teleconference zoom, they were accompanied by three team members with each job desk either as a moderator, a note-taker, or a backup. The list of respondents in this study is shown in Table 2.

Table 2. List of Research Respondents

No	Representative	Name	Institution	Affiliation
1	Association	KH	National Zakat Forum in East Java	Leader
2		BS	National Zakat Forum	Leader
3	Practitioner	CW	Zakat Institution "*Manajemen Infak*"	Director of mobilization, research, and transformation of Zakat Institution
4		MHZ	BAZNAS Centre Strategic of Studies	Director
5	Regulator	SPY	Ministry of Religious Affairs	Staff at Ministry of Religious Affairs
6		SEH	National Committee on Islamic Economy and Finance (KNEKS)	Director of Sharia Ecosystem Infrastructure
7		AJ	National Committee on Islamic Economy and Finance (KNEKS)	Director of Sharia Social Finance
8	Academics	NH	Universitas Yarsi	Professor in Islamic Economics
9		RS	Universitas Airlangga	Professor in Islamic Economics
10		MSN	Universitas Indonesia	Head of Sharia Finance Education and Human Resources Development Division
11	Community Leader	MTH	Department of Population and Civil Registration	Secretary
12		MZK	Al Mustofa Educational and Social Foundation	Founder
13		NS	Indonesian Zakat Initiative (IZI)	Program Director

Source: Author's compilation (2021)

The questionnaire of the study is in the form of a pairwise comparison of the benefits, opportunities, costs, and risks of related aspects in integrating sustainable ISF. Governance, sustainable financing, institutions, regulations, technology, and human resources are among the issues at stake in integrating the instruments of ISF. The questionnaire also comprises various solutions and strategies to achieve the research objectives. The pairwise comparison assessment in the questionnaire is rated on a scale of 1-9, where 1 (one) means not important/needed/influential and 9 (nine) means absolutely important/needed/influential.

Result

This study aimed to integrate sustainable ISF by building solutions and priority strategies in the short and long term. Before developing solutions and strategies, identification of important aspects was carried out to provide a comprehensive analysis of the most appropriate priority solutions and strategies in integrating sustainable ISF.

Priority aspect

Human resources, regulation, technology, sustainable financing, governance, and institutions are six aspects related to the integration of sustainable ISF, according to the FGD and in-depth interviews. Table 3 shows the aspects with the highest priority based on the ANP data processing.

Table 3. ANP BOCR Result of Priority Aspect

Aspect	Code	Benefit	Opportunity	Cost	Risk	Realistic	Rank
Human Resources at Islamic Social Finance Institution	A	0.1029	0.19763	0.16966	0.12334	0.97181	1
Regulation	B	0.15479	0.09765	0.11073	0.1571	0.8689	2
Supporting Technology	C	0.24925	0.16373	0.24824	0.214	0.7682	3
Sustainable Financing	D	0.1229	0.06846	0.10973	0.10029	0.7645	4
Governance	E	0.23496	0.33413	0.17927	0.25364	0.7152	5
Institution	F	0.13521	0.13841	0.18238	0.15163	0.6767	6

Source: Primary Data Prosessed (2021)

The data processing results show that human resource is the highest priority aspect in ISF institutions, with a value of 0.97181. Human resource refers to all people/staff/employees who participate in the management of ISF [20]. With a value of 0.8689, the regulation comes in as the second priority. Regulations are all rules put in place to provide legal guidance and support for the management and development of ISF [67]. With a value of 0.7682, the third priority aspect is the use of technology, while sustainable financing comes in the following priority order with a value of 0.7645. Sustainable funding is associated with business development and achieving financial independence using Islamic social funds [68]. Governance is the fifth priority aspect with a value of 0.7152. The collection, distribution, and utilization process in ISF are governed by transparency, accountability, responsibility, independence, and fairness [69]. The institutional aspect comes as the least priority, with a score of 0.6767. The institutional aspect relates to the structure and coordination system. In Indonesia, ISF institutions can take the form of government and private legal entities [70]. For zakat management, the legal entity can take in the form of Amil Zakat Institution (LAZ) and the National Amil Zakat Agency (BAZNAS), while Nazir is the legal entity for the management of waqf.

Priority solution in integrating sustainable Islamic social finance

There are eight solutions to integrate sustainable ISF based on the findings of the focus group, in-depth interviews, and literature review. The first (SL.1) is a financial integration in providing funding to beneficiaries through the distribution of zakat, waqf, and Islamic microfinance based on the beneficiaries' economic circumstances and capabilities. The second step (SL.2) is integrating ISF with state fiscal instruments, which is supported by existing regulations. Integrating Islamic social finance with the fiscal policy can be accomplished by making zakat a tax credit or tax deduction. The third (SL.3) is an integration through non-formal coordination between ISF institutions. The Islamic social finance institutions may establish joint coordinations to conduct empowerment programs, while prioritizing the work specialization of each institution. The fourth solution (SL.4) is an integration among Islamic social finance institutions based on capacity building and role division. This type of integration includes vertical and horizontal integrations [70]. The vertical integration is achieved through a collaboration between ISF institutions and the government. The government collaborates by providing a database of people who are eligible to receive Islamic social finance funds. The government also provides rules and guidance for the management of ISF. Meanwhile, a horizontal integration is conducted between ISF management institutions, where different institutions collaborate to carry out joint social programs [32]. In addition, a horizontal integration is also carried out with academics and associations for future management development of the ISF [20]. Some organizations concentrate on the health sector, while others concentrate on economic empowerment and education. As a result, combining institutions based on their specialization may be the best option for achieving the research objective. The fifth (SL.5) is establishing an integrated national data center for all ISF instruments; the data that must be integrated include financial statements, donor funds, beneficiary data, and other relevant information. The sixth (SL.6) includes an integration based on regional clusters with a solid authority. This integration may concentrate on integrating Islamic social finance institutions into geographical groups. The seventh solution (SL.7) incorporates integrating ISF through the passage of new laws and regulations. Each instrument of ISF, currently, has its own set of rules and regulations. The eighth solution (SL.8) is an integration based on ISF management clusters, such as zakat *Amils* joining other zakat institutions and waqf manager joining other waqf institutions. Table 4 shows the ANP result of solution and the order of priority solutions in the short term, long term, and based on experts' agreement

Table 4. ANP Results of Solution

Cluster	Code	Benefit	Opportunity	Cost	Risk	Net Value	Net Value Priority	Realistic	Reaslistic Priority	Expert Judment	Expert Judgment Priority
	SL.1	0.0526	0.0588	0.0466	0.04593	**0.0188**	**1**	**1.4435**	**1**	**0.2118**	**2**
	SL.2	0.0277	0.0262	0.0174	0.02957	**0.0070**	**2**	**1.4149**	**2**	0.1016	4
	SL.3	0.0258	0.0235	0.0227	0.02338	0.0032	5	**1.1452**	**3**	0.0952	5
Solution	SL.4	0.0297	0.0204	0.0226	0.023715	0.0038	4	1.1313	4	0.0929	6
	SL.5	0.0555	0.0657	0.0537	0.063225	**0.0042**	**3**	1.0737	5	**0.2438**	**1**
	SL.6	0.0244	0.0144	0.0260	0.020225	-0.0073	7	0.6719	6	0.0787	7
	SL.7	0.0159	0.0289	0.0380	0.020995	-0.0141	7	0.5777	7	**0.1071**	**3**

	SL.8	0.0180	0.0118	0.0226	0.022965	-0.0157	8	0.4113	8	0.0685	8

Source: Primary Data Processed (2021)

The formulated solution was then processed using the ANP BOCR to determine the priority order of solutions. Short term solution considers the marginal cost of the benefits obtained. On the contrary, in the long term, the priority is achieved when the total number of benefits and opportunities are obtained after reducing the total cost and risk [63,65]. On the other hand, an expert judgment does not consider the benefits, opportunity, cost, and risk aspects of each solution formulated in this study. Table 5 summarizes the results of data processing.

Table 5. Summary of Priority Solutions

Solution/Rank	Solution in Sustainable Islamic Social Finance Integration		
	1	2	3
Expert Judgment	Integration of national data centers (SL.5)	Financial integration in providing funding to beneficiaries (SL.1)	Integration through the establishmen of new laws and regulations (SL.7)
Short Term Realistic Value	Financial integration in providing funding to beneficiaries (SL.1)	Integration with state fiscal policy (SL.2)	Integration through non-formal coordination between institutions (SL.3)
Long Term Net Value	Financial integration in providing funding to beneficiaries (SL.1)	Integration with state fiscal policy (SL.2)	Integration of national data centers (SL.5)

Source: Author's compilation (2021)

According to the data processing results, the first and second priorities in the short and long-term are in the same order. The solutions that will be implemented first and foremost in the short and long term are financial integration in providing funding to beneficiaries and integration of Islamic social finance with the state fiscal policy. According to expert judgment, integrating national data centers is the highest priority solution, with financial integration in providing funding to beneficiaries coming in second.

Priority strategies in integrating sustainable Islamic social finance

There are nine strategies to integrate sustainable ISF based on the findings of the FGD, in-depth interviews, and literature review. The first strategy (ST.1) is to improve the quality of human resources through training and certification. The second strategy (ST.2) promotes the availability of real-time data through database integration and reporting. The third strategy (ST.3) is optimizing the digital technology in Islamic social finance instruments collection, management, and distribution process. The fourth (ST.4) is a coordination and synergy among stakeholders (e.g., practitioners, regulators, associations, and academicians) to establish shared commitment and vision. The fifth (ST.5) is encouraging the broadening of the range of benefits by establishing service units at lower levels, such as service units at the district, village, and mosque levels, as well as strategic locations that support the broadening of the reach of the benefits of ISF instruments. The sixth (ST.6) is establishing a new institution that is capable of integrating all instruments of ISF. In Indonesia, various institutions manage different ISF instruments. The seventh strategy (ST.7) is realizing the government's role in supporting the

integration of ISF through regulation and socialization; several previous studies have concluded that government support for integrating ISF is still inadequate. The eighth strategy (ST.8) focuses on developing the ISF institutions to have two functions, namely, *Amil* and *Nazir*. Several Baitul Maals wat Tamwiils (BMT) have operated the two functions under the same body. The ninth strategy (ST.9) is revising and updating all laws and regulations governing the management of Islamic social finance instruments to accommodate the development of ISF instruments. This development includes the management of zakat on profession, corporate zakat, temporary waqf, stock waqf, etc. The ANP results and order of priority strategies is shown in Table 6, which displays the short-term, long-term priority solutions, and expert judgment.

Table 6. ANP Results of Strategies

Cluster	Code	Benefit	Opportunity	Cost	Risk	Net Value	Net Value Priority	Realistic	Reaslistic Priority	Expert Judgment	Expert Judgment Priority
Strategies	ST.1	0.0455	0.0393	0.0377	0.030375	**0.0168**	**1**	**1.5651**	**1**	**0.1546**	**3**
	ST.2	0.0334	0.0437	0.0297	0.03745	**0.0099**	**2**	**1.3113**	**2**	0.1515	4
	ST.3	0.0500	0.0463	0.0444	0.048875	**0.0030**	**3**	**1.0676**	**3**	**0.1885**	**1**
	ST.4	0.0364	0.0450	0.0443	0.03603	0.0011	4	1.0289	4	**0.1666**	**2**
	ST.5	0.0235	0.0148	0.0181	0.02406	-0.0038	6	0.8001	5	0.0752	6
	ST.6	0.0086	0.0087	0.0089	0.01088	-0.0023	5	0.7807	6	0.0365	9
	ST.7	0.0173	0.0179	0.0213	0.019505	-0.0055	7	0.7485	7	0.0750	7
	ST.8	0.0214	0.0140	0.0203	0.02178	-0.0066	8	0.6796	8	0.0723	8
	ST.9	0.0135	0.0199	0.0250	0.02105	-0.0126	9	0.5124	9	0.0795	5

Source: Data Primary Result (2021)

After developing the solution, a strategy must be developed. Strategies are employed to aid in the achievement of solutions, and thus, the integration of ISF will be realized. The strategies are summarized in Table 7.

Table 7. Summary of Priority Strategies

**Strategies**	**Strategies in Sustainable Islamic Social Finance Integration**		
	**1**	**2**	**3**
Expert Judgment	Optimizing the use of digital technology (ST.3)	Coordination and synergy between stakeholders (ST.4)	Improving the quality of human resources (ST.1)
Short Term Realistic Value	Improving the quality of human resources (ST.1)	Establishing real time information through database integration and reporting (ST.2)	Optimizing the use of digital technology (ST.3)
Long Term Net Value	Improving the quality of human resources (ST.1)	Establishing real time information through database integration and reporting (ST.2)	Optimizing the use of digital technology (ST.3)

Source: Author's compilation (2021)

The results show that the short and long-term strategies are in the same order. Improving the quality of human resources, establishing real-time database integration and reporting, and optimizing the use of digital technology, become the priority strategies.

However, the expert judgment considers the optimization use of digital technology to be the priority strategy.

Discussion

Priority solution in developing a sustainable Islamic social finance integration model

Financial integration in providing financing to beneficiaries is a high-priority solution that can be implemented in the short and long term. Further, this solution is very consistent with the sustainability paradigm, where the funding provided will broaden the range of benefits, and ultimately creates financial independence for the beneficiaries, as stated by Haneef et al. [45], Azganin et al. [71], Thaker et al. [72], and Widiastuti et al. [20]. Sari et al. [73] show that by distributing zakat, the time taken by the poor to exit poverty is faster (3.3 years) than without zakat (6.6 years). In addition, a study conducted by Widiastuti et al. [3] analyzed financing programs carried out by the ISF institutions, including Yatim Mandiri, Dompet Dhuafa, Inisiatif Zakat Indonesia, Nurul Hayat, YDSF, and Dompet Amanah Umat, and found that they have a significant impact on increasing the welfare of the poor. The distribution aspect, particularly productive distribution, holds the key to success in ISF integration in transforming the poor into the capables [69]. A financial integration through ISF instruments, such as zakat and waqf, will increase production factors. In this case, financing will be more effective due to an increase in the amount of funds obtained by the poor to achieve welfare. This is consistent with the economic integration theory discussed by Balassa [57]. This also supports Sanyinna and Omar [74] claim that Islamic financing benefits small businesses by maximizing Islamic social financial instruments (zakat, *infaq*, *sadaqah*, and waqf) as the best strategy for improving the recipients' quality of life. The financing provided through integrating Islamic social finance instruments has a cheap cost of the fund, and thus, it ensures the beneficiaries' welfare. Furthermore, the integration of financing demonstrates that Islamic social fund instruments serve multiple purposes: Consumptive zakat is used to provide an emergency assistance in the short term, while productive zakat and benevolence funds are used to support economic recovery in the medium term, and waqf or Sukuk are used to build long-term resilience. The financial integration of Islamic social finance instruments has also occurred in several institutions, although only on a micro-scale. Several institutions in Indonesia have worked to integrate zakat and waqf funds. Dompet Dhuafa, for example, integrated zakat and waqf funds when constructing an Integrated Health Home (Hospital). Cash Waqfs are used to build infrastructure on waqf land, whereas zakat funds provide free health care to the poor. Further, recently, Dompet Dhuafa, BAZNAS, and BWI have collaborated to integrate zakat and waqf, the newly established MoU, to collaborate on social projects.

This priority, however, differs from expert opinion, which emphasizes the importance of integrating nationally centralized data, such as database on donors, beneficiaries, and financial reports. According to Afriadi and Sanrego [54] and Hudaefi et al. [75] the formation of national-centered data is critical for optimizing the integration and management of ISF . The integration of beneficiary-related information is crucial for detecting data on beneficiary development and preventing overlapping distributions. Since ISF are still managed in a fragmented manner (partially), no single platform currently integrates the data from all instruments of ISF in Indonesia. As the latest development, a data integration platform has been

established in the zakat sector. BAZNAS, an Indonesian zakat institution and regulator, released SIMBA (BAZNAS Information Management System), an integrated platform for zakat database management from all BAZNAS and LAZNAS across Indonesia. This platform only integrates the data from national zakat collections. As a result, establishing a central platform capable of integrating data on all Islamic social financial instruments is critical.

Financial integration and integration of national data centers are interrelated. This is so since funding distribution necessitates specific data to ensure the long-term viability of the funds provided. Data integration is needed to reduce the potential risk and realize the collection of distributed financings so that the supply of funds will be guaranteed. Further, the integration of beneficiary data is needed to ensure the depth of the reach of benefits and the creation of financial independence, and thus, sustainability will be achieved. On another side, financial integration remains a short- and long-term priority because it is directly related to the primary goal of Islamic social finance, which is to create prosperity by improving the business and economic capacity of beneficiaries. As one of the priority solutions in this research, the regulator must provide a legal environment to accommodate all changes and developments in the management of Islamic social finance. First, the government must revise the law to accommodate the rapid growth of Islamic social finance instruments [76]. Second, the government must socialize the revised laws and regulations to all stakeholders, and make the law more socially acceptable [20,77]. Third, encourage ISF management institutions to implement the law by offering rewards or incentives that the Institution can use, such as free training and accompaniment [20].

Strategic priorities in developing a sustainable Islamic social finance integration model

According to expert judgments, the strategy with the highest priority is optimizing the use of digital technology. This in line with Thaker et al. [78], Usman et al. [79], Berakon et al. [80], Nor et al. [81], and Widiastuti et al. [20], all of which emphasize the importance of using technology in managing the instrument of ISF. First, technology for creating a centralized database linked to the government and ISF supervisors is one example of a technology that can be used [20]. This strategy is also linked to the immediate solution, which is the consolidation of national data centers for all Islamic social and financial instruments. To become effective and efficient, data integration necessitates the creation of a sophisticated web-based platform that all stakeholders can access. Second, technological advancements in supervision and monitoring [20]. Third, technology in the marketing component to aid in the collection of target achievement [82,83]. Fourth, technology to generate real-time management reports and make them available to the public [20].

However, in terms of short-term and long-term priorities, optimizing the use of technology has slipped to third place. Experts consider improving the quality of human resources to be the most important aspect, both in the short and long term. The importance of enhancing the capability of human resources goes in line with Karami et al. [84], Hidayatullah and Priantina [21], Ali et al. [55], and Widiastuti et al. [20]. The difference in priorities between expert judgments and short/long term orientations occurs because of technology investment, which is considered a long-term investment, necessitates a significant funding source, which

some zakat institutions may not afford [20]. Optimizing the use of technology, which requires a large amount of investment, is a challenge for Islamic social finance institutions, particularly in Indonesia. This is due to the fact that the operational costs incurred by ISF institutions are determined by the amount of funds that can be raised. Meanwhile, there is a significant gap between the realization and potential of ISF. As a result, institutions prefer to improve competencies and capabilities of human resources, relevant to their respective fields of work compared to improving technological features. This is also consistent with the aspects of BOCR addressed in this study. The cost of optimizing technology use is 0.0537, higher than the cost of improving the quality of human resources, which is around 0.0466. Costs associated with technology investments include acquiring digital channels, website management, social media, purchase of computers, and maintenance cost.

Furthermore, when determining priority strategies in the short and long term, experts also consider the risks exposed. The risk of optimizing the use of technology is high (0.0632), with the benefits obtained are also higher (0.0555). Meanwhile, the threat from the institution's strategy to improve the quality of human resources is lower at 0.0459. Cybercrime is one of the risks of optimizing the use of technology in ISF [85]. To mitigate this risk, competent human resources in their field are required. Implementing technology without qualified human resources will result in sub-optimal management. This is consistent with the findings of Widiastuti et al. [20] that a limited number of human resources is capable of optimizing the use of advanced technology, particularly in those newly established institutions. Therefore, experts place qualified human resources as the prioritized strategy to mitigate risks, not only risks associated with technology application, but all risks associated with the integration of sustainable Islamic social finance.

Optimizing the use of technology and improving human resource quality can be done concurrently as stated by Widiastuti et al. [20] that highlights the importance of improving the quality of zakat management resources, particularly in the technological aspect, by emphasizing technology intensification (by developing *Amils*' ability to use technology) and extensification (by increasing the number of *Amils* who master technology). Improving the quality of human resources is the foundation for the long-term integration of ISF, while technology facilitates an optimal ISF management.

Finally, in order to integrate sustainable ISF, stakeholders must prioritize solutions and strategies, by considering the benefits, opportunities, costs, and risks (BOCR) of each priority solution and strategy. Improving the quality of human resources and optimizing the use of digital technology are two strategies that must be implemented immediately. Based on the short- and long-term analysis and expert consensus, the two strategies are given the highest priority.

Managerial relevancy

This study has several implications for stakeholders. The findings of this study emphasize solutions and priority strategies for integrating ISF, with Islamic social finance institutions playing a critical role in providing certification and training to improve the quality of human resources. Each training activity and accreditation must be given in accordance with the employee's field of work. Technology optimization, such as big data management, data security, e-marketing, and others, are examples of certification and training that must be

provided. Human resource quality must be directed to obtain management and technical skills. Managers are encouraged to have managerial skills, such as making strategic decisions, forecasting, and imposing rewards and punishments. Staff are encouraged to learn technical skills, such as collection program operation, *mustahik* guidance, reporting, and administration.

The government, as a regulator, must assist Islamic social finance institutions in providing facilities or infrastructure to improve the quality of human resource managers, such as free training and certification for managers. Further, no law or regulation has required ISF managers to have standardized or specific competencies. As a result, one form of input for the revision of the law and regulation requires all ISF managers to have specific competencies in managing Islamic social finance institutions so that these institutions can be managed more professionally.

This study also emphasizes the significance of financial integration and data integration on the national scale. Associations involved in Islamic social finance can use the research findings to establish strategic planning on integrating ISF instruments. From an academic standpoint, the results of this study develop the previous studies on the integration of the ISF model, which were conducted without using specific mathematical methods. This study used a systematic approach to create an ideal integration model that can be of priorities both in the short and long term, while also taking the aspect of BOCR into account.

Conclusion

This research used the ANP BOCR to develop priority solutions and strategies to integrate Islamic social finance based on expert agreement, short-term and long-term analysis. The aspect of human resources, regulation, and technology use become the highest aspect to be considered in achieving the research objective. Priority solutions in the short and long term have the same order of priorities. Financial integration in providing funding to beneficiaries and integration with the state fiscal policy is the first and second priority solution. Meanwhile, integration through a non-formal coordination between institutions is the third priority solution in the long term. Besides, integrating national data centers has been the third priority solution for a long term and the highest solution based on expert judgments. In essence, the resulting priority solutions relate to priority aspects include financing, regulation, and technology.

As a strategy, improving the quality of human resources, establishing real-time information through database integration and reporting, and optimizing digital technology are three priority strategies that can be implemented in the short and long term. Furthermore, the experts mentioned the need for coordination and synergy between stakeholders in integrating sustainable Islamic social finance. To successfully incorporate Islamic social finance and ensure sustainability, stakeholders are encouraged to implement the research results (solutions and strategies).

Limitation and future research

This research only focuses on six aspects to develop ideal solutions and strategies, however, this research has thoroughly examined each aspect's benefits, opportunities, costs, and risks. Future research can consider essential elements that have not been addressed in developing a sustainable Islamic social finance integration model, such as cultural aspects, donor behavior, and government support.

Acknowledgment

The authors would like to thank Center Research and Publication (3P) of Faculty Economic and Business Universitas Airlangga as ethics committee. The authors would also like to thank all academicians, regulators, associations, practitioners, and community leaders for their support in this research. The authors also appreciate the research team members for their support in the completion of this research.

References

1. Ibrahim P, Ghazali R. Zakah as an Islamic micro-financing mechanism to productive zakah recipients. Asian Econ Financ Rev 4(1), 117-125. 2014;4(1):117–25.
2. Zauro NA, Saad RAJ, Ahmi A, Mohd Hussin MY. Integration of waqf towards enhancing financial inclusion and socio-economic justice in Nigeria. Int J Ethics Syst. 2020;36(4):491–505. doi: 10.1108/IJOES-04-2020-0054
3. Widiastuti T, Auwalin I, Rani LN, Mustofa MU Al. A mediating effect of business growth on zakat empowerment program and mustahiq's welfare. Cogent Bus Manag. 2021;8(1):1882039. doi: 10.1080/23311975.2021.1882039
4. Abdullah M. Waqf, Sustainable Development Goals (SDGs) and maqasid al-shariah. Int J Soc Econ. 2018;45(1):158–72. doi: 10.1108/IJSE-10-2016-0295
5. Ascarya A. The role of Islamic social finance during COVID-19 pandemic in Indonesia's economic recovery. Int J Islam Middle East Financ Manag. 2021; doi: 10.1108/IMEFM-07-2020-0351
6. Robani A, Salih K. Positioning Islamic gift economy for sustainable development at the local level. Humanit Soc Sci Rev. 2018;6(2):111–20. doi: 10.18510/hssr.2018.6214
7. Abdullahi A, Othman AHA, Kassim S. Financial inclusion enhancement through the adoption of Islamic microfinance in nigeria. Int J Ethics Syst. 2021;37(3):486–505. doi: https://doi.org/10.1108/IJOES-02-2021-0040
8. Raies A. Islamic versus conventional fiscal policy: The effect of zakat on education and employment. Acad J Interdiscip Stud. 2020;9(1):27–33. doi: 10.36941/ajis-2020-0003
9. Rusydiana AS, Sukmana R, Laila N. Waqf on education: a bibliometric review based on Scopus. Libr Philos Pract. 2021;2021(June):5537.
10. Suprayitno E, Aslam M, Harun A. Zakat and SDGs : Impact zakat on human development in the five states of Malaysia. Int J Zakat. 2017;2(1):61–9.
11. Riyanto S, Herningtyas R, Nizar M. Muslim's philanthropy in Singapore and the role of government. Sociolohia Y Tecnociencia. 2021;11(2):194–214. doi: https://doi.org/10.24197/st.2.2021.194-214
12. IFSB. Islamic financial services industry stability report. Malaysia; 2021.
13. Gärde J. Concepts on zakat, carita, and diaconia in the changing social welfare landscape of europe. J Relig Spiritual Soc Work Soc Thought. 2017;36(1–2):164–98. doi: 10.1080/15426432.2017.1311242
14. Gatto M, Islam AHMS. Impacts of COVID-19 on rural livelihoods in Bangladesh : evidence using panel data. PLoS One. 2021;16(11):e0259264. doi: 10.1371/journal.pone.0259264

15. Suryahadi A, Izzati R Al, Suryadarma D. Estimating the impact of COVID-19 on poverty in Indonesia*. Bull Indones Econ Stud. 2020;0(0):175–92. doi: 10.1080/00074918.2020.1779390

16. World Bank. From containment to recovery [Internet]. World Bank. 2020. Available from: https://openknowledge.worldbank.org/bitstream/handle/10986/34497/9781464816413.pdf

17. Ministry of Finance. APBN kita: Kinerja dan fakta. Kemenkeu.Go.Id. 2020.

18. Ministry of Finance. Survei dan analisis insentif perpajakan program PEN 2020 tunjukkan dampak positif. 2021.

19. BAZNAS. Outlook zakat indonesia 2021. Jakarta: Pusat Kajian Strategis - Badan Amil Zakat Nasional (PUSKAS BAZNAS); 2021. 1–144 p.

20. Widiastuti T, Cahyono EF, Zulaikha S, Mawardi I, Al Mustofa MU. Optimizing zakat governance in East Java using Analytical Network Process (ANP): The role of Zakat Technology (ZakaTech). J Islam Account Bus Res. 2021;12(3):301–19. doi: 10.1108/JIABR-09-2020-0307

21. Hidayatullah A, Priantina A. Toward zakat management integration in Indonesia: problems and solution. Ahkam J Ilmu Syariah. 2018;18(2):321–46. doi: 10.15408/ajis.v18i2.6319

22. Amalia E. The shariah governance framework for strengthening zakat management in Indonesia: a critical review of zakat regulations. In: Advances in Social Science, Education and Humanities Research. 2018. p. 133–8. doi: 10.2991/iclj-17.2018.28

23. Center of Strategic Studies BAZNAS. Indeks literasi zakat: Teori dan konsep. Jakarta Pusat: Pusat Kajian Strategis – Badan Amil Zakat Nasional (Puskas); 2019. 1–46 p.

24. Pitchay AA, Thaker MAMT, Mydin AA, Azhar Z, Latiff ARA. Cooperative-waqf model: A proposal to develop idle waqf lands in Malaysia. ISRA Int J Islam Financ. 2018;10(2):225–36. doi: 10.1108/IJIF-07-2017-0012

25. Ahmad M. An empirical study of the challenges facing zakat and waqf institutions in Northern Nigeria. ISRA Int J Islam Financ. 2019;11(2):338–56. doi: 10.1108/IJIF-04-2018-0044

26. Sukmana R. Critical assessment of islamic endowment funds (waqf) literature: Lesson for government and future directions. Heliyon. 2020;6(10):e05074. doi: 10.1016/j.heliyon.2020.e05074

27. Ali KM. Integrating zakah, awqaf and IMF for poverty alleviation: Three models of Islamıc micro finance. Irti. 2014;2(3):193–211. doi: 10.13140/RG.2.2.31888.40969

28. Lubis M, Azizah AH. Towards achieving the efficiency in zakat management system: interaction design for optimization in Indonesia. Commun Comput Inf Sci. 2018;886(March 2017):vi. doi: 10.1007/978-981-13-1628-9

29. IRTI. Islamic social finance report 2014. Islam and the Moral Economy. 2014. doi: 10.1017/cbo9780511617614.005

30. Sudirman. Government policy on zakat and tax in Indonesia. AHKAM J Ilmu Syariah. 2015;15(1):1–14.

31. Raimi L, Patel A, Adelopo I. Corporate social responsibility, waqf system and zakat

system as faith-based model for poverty reduction. World J Entrep Manag Sustain Dev. 2014;10(3):228–42. doi: 10.1108/wjemsd-09-2013-0052

32. Jouti AT. An integrated approach for building sustainable Islamic social finance ecosystems. ISRA Int J Islam Financ. 2019;11(2):246–66. doi: 10.1108/IJIF-10-2018-0118

33. Shalleh MCM, Razali SS, Laksana NNM, Embi NAC, Abdullah NI. Developing a sustainable model of waqf-based takaful for flood victims in Malaysia. J Islam Account Bus Res. 2020;11(9):1941–52. doi: 10.1108/JIABR-10-2016-0114

34. IRTI. Islamic social finance report 2020. 2020.

35. Kuanova LA, Sagiyeva R, Shirazi NS. Islamic social finance: A literature review and future research directions. J Islam Account Bus Res. 2021;12(5):707–28. doi: 10.1108/JIABR-11-2020-0356

36. Mateev M, Tariq MU, Sahyouni A. Competition, capital growth and risk-taking in emerging markets: Policy implications for banking sector stability during COVID-19 pandemic. PLoS One. 2021;16(6):e0253803. doi: 10.1371/journal.pone.0253803

37. Arikan G, Bloom PB-N. "I was hungry and you gave me food": Religiosity and attitudes toward redistribution. PLoS One. 2019;14(3):e0214054. doi: 10.1371/journal.pone.0214054

38. Adjar H, Chakir A, Meskine L El. Exploratory study of Islamic social finance in Morocco. Int Rev Entrep Financ. 2020;3(2):45–69.

39. Hudaefi FA. How does Islamic fintech promote the SDGs? Qualitative evidence from Indonesia. Qual Res Financ Mark. 2020;12(4):353–66. doi: 10.1108/QRFM-05-2019-0058

40. Kailani N, Slama M. Accelerating Islamic charities in indonesia: Zakat, sedekah and the immediacy of social media. South East Asia Res. 2020;28(1):70–86. doi: 10.1080/0967828X.2019.1691939

41. Kasri RA. Management of zakah distribution: Empirical evidence from Indonesia. AFEBI Islam Financ Econ Rev. 2017;

42. Bilo C, Machado AC. The role of zakat in the provision of social protection: A comparison between Jordan and Sudan. Int J Sociol Soc Policy. 2020;40(3–4):236–48. doi: 10.1108/IJSSP-11-2018-0218

43. Manurung S. Islamic religiosity and development of zakat institution. Qudus Int J Islam Stud. 2013;1(2):197–220. doi: 10.21043/QIJIS.V1I2.186

44. Arifin N, Anwar AZ. The improvement model of microenterprises of post-disaster through empowerment of productive zakat. J Gov Regul. 2021;10(4):156–63. doi: 10.22495/jgrv10i4art14

45. Haneef MA, Pramanik AH, Mohammed MO, Bin Amin MF, Muhammad AD. Integration of waqf-Islamic microfinance model for poverty reduction: The case of bangladesh. Int J Islam Middle East Financ Manag. 2015;8(2):246–70. doi: 10.1108/IMEFM-03-2014-0029

46. Pischke JD Von. Measuring the trade-off between outreach and sustainability of microenterprise lenders. J Int Dev. 1996;8(2):225–39. doi: 10.1002/(SICI)1099-1328(199603)8:2<225::AID-JID370>3.0.CO;2-6

47. Chow TC, Zailani S, Rahman MK, Qiannan Z, Bhuiyan A, Patwary AK. Impact of sustainable project management on project plan and project success of the manufacturing firm: Structural model assessment. PLoS One. 2021;16(11):e0259819. doi: 10.1371/journal.pone.0259819

48. Brau JC, Woller GM, Brau JC, Woller GM. Microfinance: A comprehensive review of the existing literature. J Entrep Financ. 2004;9(1):1–28.

49. Ali AFM, Rashid ZA, Johari F, Aziz MRA. The effectiveness of zakat in reducing poverty incident: An analysis in Kelantan, Malaysia. Asian Soc Sci. 2015;11(21):355–67. doi: 10.5539/ass.v11n21p355

50. Ahmed BO, Johari F, Wahab KA. Identifying the poor and the needy among the beneficiaries of zakat need for a zakat-based poverty threshold in Nigeria. Int J Soc Econ. 2017;44(4):446–58. doi: 10.1108/IJSE-09-2015-0234

51. Bin-Nashwan SA, Abdul-Jabbar H, Aziz SA, Viswanathan KK. A socio-economic model of zakah compliance. Int J Sociol Soc Policy. 2020;40(3–4):304–20. doi: 10.1108/IJSSP-11-2019-0240

52. Amalia E. Good governance for zakat institutions in Indonesia: a confirmatory factor analysis. Pertanika J Soc Sci Humanit. 2019;27(3):1815–27.

53. Nguyen-Van P, Stenger A, Tiet T. Social incentive factors in interventions promoting sustainable behaviors : a meta- analysis. PLoS One. 2021;16(12):e0260932. doi: 10.1371/journal.pone.0260932

54. Afriadi F, Sanrego YD. Mengurai permasalahan distribusi zakat kepada fakir miskin: pengalaman organisasi pengelola zakat BAZNAS, Dompet Hhuafa, dan LAZIZMU. J Madania. 2016;20(1):23–36. doi: 10.29300/madania.v20i1.83

55. Ali MM, Devi A, Furqani H, Hamzah H. Islamic financial inclusion determinants in Indonesia: An ANP approach. Int J Islam Middle East Financ Manag. 2020;13(4):727–47. doi: 10.1108/IMEFM-01-2019-0007

56. Ryandono MNH, Qulub AS, Cahyono EF, Widiastuti T, Aisyah BN, Robani A. Efficiency analysis of zakat institutions in Indonesia: Data Envelopment Analysis (DEA) and Free Disposal Hull (FDH) approaches. Acad Account Financ Stud J. 2021;25(6):1–12.

57. Balassa B. Economic integration. world Econ. 1991;176–86. doi: 10.47577/technium.v2i4.860

58. Kang M, Yang MG (Mark), Park Y, Huo B. Supply chain integration and its impact on sustainability. Ind Manag Data Syst. 2018; doi: 10.1108/IMDS-01-2018-0004

59. Bong A, Premaratne G. The impact of financial integration on economic growth in Southeast Asia. J Asian Financ Econ Bus. 2019;6(1):107–19. doi: 10.13106/jafeb.2019.vol6.no1.107

60. Hassanain KM. Integrating zakah, awqaf, and IMF for poverty allevation: Three models of Islamic micro finance. J Econ Soc Thought. 2015;2(2):106–20.

61. Ambrose AHAA, Hassan MAG, Hanafi H. A proposed model for waqf financing public goods and mixed public goods in Malaysia. Int J Islam Middle East Financ Manag. 2018;11(3):395–415. doi: 10.1108/IMEFM-01-2017-0001

62. Saaty TL. Decision making with the analytic network process. In: International Series in Operations Research & Management Science. Springer; 2013. p. 1–40.

63. Saaty TL, Vargas LG. Decision making with the analytic network process: Economic, political, social and technological application with benefit, opportunities, cost and risks. II. Vol. 195, International Series in Operations Research and Management Science. London: Springer; 2006. 1–370 p. doi: 10.1007/978-1-4614-7279-7_15

64. Ascarya. Analytic Network Process (ANP): Pendekatan baru studi kualitatif. Cent Cent Bank Educ Stud Bank Indones. 2005;

65. Zammori F, Bigliardi B, Carmignani G. Selecting inbound logistic policies: An ANP-based multi criteria decision making approach. Int J Logist Syst Manag. 2014;19(2):212–44. doi: 10.1504/IJLSM.2014.064659

66. Firmansyah I, Sukmana W. Analisis problematika zakat pada BAZNAS Kota Tasikmalaya: Pendekatan metode Analytic Network Process (ANP). J Ris Akunt dan Keuang. 2014;2(2):392. doi: 10.17509/jrak.v2i2.6593

67. Munandar A, Syakhroza A, Martani D, Siswantoro D. Does regulation increase zakat payment? Acad Account Financ Stud J. 2019;23(6).

68. Sulaiman M, Zakari MA. Financial sustainability of State Waqf Institutions (SWIs) in Malaysia. J Islam Account Bus Res. 2019;10(2):236–58. doi: 10.1108/JIABR-05-2016-0054

69. Adiwijaya ZA, Suprianto E. Good governance of zakat institutions: A literature review. J Southwest Jiaotong Univ. 2020;55(2):1–7. doi: 10.35741/issn.0258-2724.55.2.38

70. Minister of Law and Human Rights Republic of Indonesia. Undang-undang republik indonesia nomor 23 tahun 2011 tentang pengelolaan zakat. 2011.

71. Azganin H, Kassim S, Auwal Adam S. Proposed waqf crowdfunding models for small farmers and the required parameters for their application. Islam Econ Stud. 2021; doi: 10.1108/IES-01-2021-0006

72. Thaker MAMT, Amin MF, Thaker HMT, Khaliq A, Pitchay AA. Cash waqf model for micro enterprises' human capital development. ISRA Int J Islam Financ. 2021;13(1):66–83. doi: 10.1108/ijif-08-2018-0091

73. Sari DF, Beik IS, Rindayati W. Investigating the impact of zakat on poverty alleviation : A case from West Sumatra, Indonesia. Int J Zakat. 2019;4(2):1–12. doi: https://doi.org/10.37706/ijaz.v4i2.180

74. Sanyinna AY, Omar HH. Employing cash waqf in financing micro, small and medium enterprises for sustainable poverty alleviation-a grounded theory approach. In: Proceedings of International Conference on Zakat, Tax, Waqf And Economic Development (ZAWED). 2019. p. 37–48.

75. Hudaefi FA, Caraka RE, Wahid H. Zakat administration in times of COVID-19 pandemic in Indonesia: a knowledge discovery via text mining. Int J Islam Middle East Financ Manag. 2021; doi: 10.1108/IMEFM-05-2020-0250

76. Mutamimah M, Alifah S, Gunawan G, Adnjani MD. ICT-based collaborative framework for improving the performance of zakat management organisations in Indonesia. J Islam Account Bus Res. 2021; doi: 10.1108/jiabr-05-2020-0154

77. Saad RAJ, Farouk AU. A comprehensive review of barriers to a functional zakat system in Nigeria: what needs to be done? Int J Ethics Syst. 2019;35(1):24–42. doi: 10.1108/IJOES-06-2018-0090

78. Thaker MAMT, Thaker HMT, Ptchay AA. Modeling crowdfunders' behavioral

intention to adopt the Crowdfunding-Waqf Model (CWM) in Malaysia: the theory of the technology acceptance model. Int J Islam Middle East Financ Manag. 2018;11(2):231–49. doi: 10.1108/IMEFM-06-2017-0157

79. Usman H, Mulia D, Chairy C, Widowati N. Integrating trust, religiosity and image into technology acceptance model: The case of the Islamic philanthropy in Indonesia. J Islam Mark. 2020; doi: 10.1108/JIMA-01-2020-0020

80. Berakon I, Aji HM, Hafizi MR. Impact of digital sharia banking systems on cash-waqf among Indonesian muslim youth. J Islam Mark. 2021; doi: 10.1108/JIMA-11-2020-0337

81. Nor SM, Abdul-Majid M, Esrati SN. The role of blockchain technology in enhancing islamic social finance: The case of zakah management in Malaysia. Foresight. 2021;23(5):509–27. doi: 10.1108/FS-06-2020-0058

82. Hadi R, Sochimin, Basit A. Sharia strategic economic model on digital zakat technology in Indonesia. Econ Ann. 2021;187(1/2):82–9. doi: 10.21003/EA.V187-08

83. Kasri RA, Yuniar AM. Determinants of digital zakat payments: Lessons from Indonesian experience. J Islam Account Bus Res. 2021;12(3):362–79. doi: 10.1108/JIABR-08-2020-0258

84. Karami A, Farokhzadian J, Foroughameri G. Nurses ' professional competency and organizational commitment : Is it important for human resource management? PLoS One. 2017;12(11):e0187863. doi: 10.1371/journal.pone.0187863 November

85. Ninglasari SY, Muhammad M. Zakat digitalization: effectiveness of zakat management in the COVID-19 pandemic era. J Islam Econ Laws. 2021;4(1):26–44. doi: 10.23917/jisel.v4i1.12442

Appendix. Questionnaire

Integrating sustainable Islamic social finance: An Analytical Network Process using the Benefit Opportunity Cost Risk (ANP BOCR) framework: The case of Indonesia

This questionnaire aims to develop solutions and strategies to build a sustainable Islamic Social Finance integration model by considering the benefits, opportunity costs and risks (BOCR) of the model built. This questionnaire aims to collect primary data in order to develop the Analytical Network Process model. It is hoped that in this study the strategy can be developed using the Analytical Network Process.

This questionnaire is the primary data collection stage for the research “Sustainable Islamic Social Finance Model: Strategic Solution in Countering Adverse Impacts of Social Economy of The Covid-19 Using Analytical Network Process Benefit Opportunities Cost Risk. This questionnaire is designed to determine the personal assessment of key informants, which include practitioners, experts/experts, government representatives, academics, community leaders, and associations. There is no right or wrong answer, but this assessment aims to compare the level of importance or priority of each attribute to a problem. The rating scale will be explained as follows:

1 = Not important or not influential

2-3= Less important or less influential

4-5= Important or influential

6-7= Very important or very influential

8-9= Absolutely important or absolutely influential

SECTION 1: IMPORTANT ASPECT

1. Looking at the following aspects, **how important are these aspects in the integration of Islamic Social Finance?**
   a. Governance
   b. Sustainable financing
   c. Institutional
   d. HR Islamic Social Finance Institute
   e. Regulation
   f. Use of Technology

2. In your opinion, from the four aspects below, **which aspect has the highest priority compared to all aspects**?
   a. Benefit
   b. Opportunity
   c. Cost
   d. Risk

SECTION 2: ANALYSIS OF BENEFIT, OPPORTUNITY, COST, RISK (BOCR)

BENEFIT

1. The following are several of **benefits from the governance aspect in supporting the creation of Islamic Social Finance integration**, how important are the benefits below in supporting the creation of Islamic Social Finance integration?
   a. There is an integration of Islamic social finance into commercial enterprises
   b. There is an MoU between BAZNAS and BWI to integrate zakat and waqf
   c. Many previous studies support the governance of zakat and waqf
   d. There are already standards for zakat and waqf management
   e. Some programs increase the motivation of donors to channel funds to Islamic social, financial institutions
   f. Every Islamic social finance institution has a supervisor so that the institution's operations run according to the plans and applicable rules
   g. The existence of a sharia supervisory board makes decisions more appropriate and following sharia rules
   h. Strengthening SDGs for Islamic social finance institutions will provide clear and measurable goals
   i. There are financial reporting standards for Islamic social financial institutions
   j. National data integration
   k. Increased effectiveness and efficiency in the use of operational funds

2. The following are several **benefits from the aspect of sustainable financing in supporting the creation of the integration of Islamic Social Finance**; how important are the benefits below in supporting the creation of the integration of Islamic Social Finance?
   a. The availability of cheap funds sourced from zakat, *infaq*, alms, waqf will make it easier for MSMEs to get financing
   b. Financing carried out at BMT includes social and financial functions commercial to increase the competitiveness of BMT
   c. There are many contracts in financing (*qard, ijarah, mudharabah*, etc.) that can reach a wider target recipient.

3. The following are several **benefits from the institutional aspect in supporting the creation of Islamic Social Finance integration**; how important are the benefits below in supporting the creation of Islamic Social Finance integration?
   a. The broad reach of each Islamic social financial institution spread across Indonesia
   b. The existence of branch offices or representative offices in various regions provides a broader range of benefits
   c. The ZISWAF Fund managed by the institution becomes more organized
   d. Islamic social finance institutions established by the community/social community have the potential to develop faster
   e. The increasing interest of donors to pay ZISWAF in institutions
   f. There is an association in Islamic social fund institutions

4. The following are several **benefits from the HR aspect in the Institution in supporting the creation of the integration of Islamic Social Finance**; how important are the benefits below in supporting the creation of the integration of Islamic Social Finance?
   a. Human resource loyalty in Islamic social fund management institutions occurs because of the high level of religiosity
   b. Increase professionalism of human resource

5. The following are several **benefits from the regulatory aspect in supporting the creation of Islamic Social Finance integration**; how important are the benefits below in supporting the creation of Islamic Social Finance integration?
   a. ZISWAF rules in Indonesia are more complete compared to other countries
   b. The regulations do not prohibit one institution from having a dual role, namely as a nadzir and amyl
   c. There is political support from the government executive board for the optimization of ZISWAF

6. The following are several **benefits of using technology in supporting the creation of the integration of Islamic Social Finance**; how important are the benefits below in supporting the creation of the integration of Islamic Social Finance?
   a. The use of technology makes it easier to raise and distribute funds
   b. Availability of platforms for national data integration so that data becomes accurate
   c. Access to information becomes faster and easier
   d. Technology provides a wider range of socialization

OPPORTUNITY

1. The following are several **opportunities from the governance aspect in supporting the creation of Islamic Social Finance integration**; how important are the opportunities below in supporting the creation of Islamic Social Finance integration?
   a. Achieved realization of the potential for collecting zakat and waqf in Indonesia
   b. The collaboration of ZISWAF funds with BMT will present optimal Islamic social finance management
   c. Improving the performance and effectiveness of Islamic social finance institutions
   d. The emergence of a more comprehensive performance appraisal
   e. The higher contribution of Islamic social finance in achieving the SDGs, especially the needs during the pandemic
   f. Increasing the image of Islamic social financial institutions
   g. Increased transparency of social financial management
   h. Availability of accurate information for stakeholders
   i. Increased public trust in Islamic social financial institutions
   j. Increased capacity building
   k. The ability of Islamic social financial institutions to carry out their duties and functions effectively and efficiently

2. The following are several **opportunities from the aspect of sustainable financing in supporting the creation of the integration of Islamic Social Finance**; how important are the opportunities below in supporting the integration of Islamic Social Finance?
   a. Ease of accessibility to Islamic social financial institutions, both in urban and rural areas
   b. The creation of a sustainable Islamic social finance ecosystem
   c. The level of funding needs is high, especially in the productive sector.
   d. Increased profit and income from financing
   e. Increased productivity of both the recipient of the financing or the funds used
   f. The decrease in the total poverty zone in Indonesia, which is in line with the SDGs

3. The following are several **opportunities from the institutional aspect in supporting the creation of Islamic Social Finance integration**; how important are the opportunities below in supporting the creation of Islamic Social Finance integration?
   a. The diversity of Islamic social financial institutions further optimizes ZISWAF funds
   b. The increasing number of Islamic social financial institutions spread to the lower levels
   c. The existence of Islamic social financial institutions or services to the lowest level, for example: villages, etc.

4. The following are several **opportunities from the HR aspect in the Institution in supporting the creation of Islamic Social Finance integration**; how important are the opportunities below in supporting the creation of Islamic Social Finance integration?
   a. Increasing the number of human resources who have competence in the management of Islamic social finance
   b. Increasing the competitiveness of institutional HR
   c. Availability of new job opportunities and increased absorption of labor

5. The following are a number of **opportunities from the regulatory aspect to support the creation of the integration of Islamic Social Finance**, how important are the opportunities below in supporting the creation of the integration of Islamic Social Finance?
   a. There are opportunities for regulatory development if needed
   b. Increased government support in innovative ZISWAF development and optimization of Islamic social finance
   c. Improved relations between OIC countries related to the management of Islamic social finance

6. The following are several **opportunities from using technology to support the integration of Islamic Social Finance**; how important are the opportunities below in supporting the creation of the integration of Islamic Social Finance?
   a. Increased public understanding of technology
   b. Increasing the effectiveness and efficiency of Islamic social finance management

COST

1. The following are several **costs on the aspects of governance needed in the integration of Islamic Social Finance**, how much are the costs below in the integration of Islamic Social Finance?
   a. There is a high-cost requirement to improve people's literacy
   b. Islamic social finance management requires high costs, one of which is related to infrastructure development, provision of tools, operational costs to monitoring systems
   c. The existence of the Sharia Supervisory Board (DPS) makes the bureaucratic flow take a long time
   d. The high cost of conducting a public audit
   e. Preparation of standardized reports takes quite a long time
   f. The gap between academics and practitioners in the practice of managing Islamic social finance
   g. Inefficient administration

2. The following are several **costs from the sustainable financing aspects needed in the integration of Islamic Social Finance**, how much are the costs below in the integration of Islamic Social Finance?
   a. High service fees for Islamic financing
   b. The position of Islamic social financial institutions is not strong, so they are very dependent on other intermediary institutions
   c. Public perception that Islamic financing still contains usury

   d. High marketing costs for strengthening Islamic financing literacy in Islamic social financial institutions.

3. The following are several **costs from the institutional aspects needed in the integration of Islamic Social Finance**, how much are the costs below in the integration of Islamic Social Finance?
   a. Differences in the pattern of government-based and community/private-based institutions
   b. Islamic social finance management through an institutional approach requires high costs

4. The following are several **costs from the HR aspect in institutions that are needed to integrate Islamic Social Finance**; how much are the costs below in the integration of Islamic Social Finance?
   a. Training for HR requires a large amount of money
   b. Mismatch in the background of the manager of Islamic social financial institutions
   c. Low knowledge of human resources in the field of financial management
   d. Limited HR mobility during the Pandemic

5. The following are several **costs from the regulatory aspect in the integration of Islamic Social Finance**, how much are the costs below in integrating Islamic Social Finance?
   a. Limitations of guidance in the management of ZISWAF when the Act is issued
   b. Differences in permits for Islamic social finance management institutions
   c. Conflict handling costs
   d. Conflict of interest between stakeholders
   e. The integration of ZISWAF management takes a long time due to political factors

6. The following are several **costs from the aspect of using technology needed in the integration of Islamic Social Finance**, how much is the cost below in the integration of Islamic Social Finance?
   a. Implementation of digital technology requires high costs
   b. The use of technology will provide challenges related to the fulfillment of sharia principles

RISK

1. The following are several **risks from the governance aspect in integrating Islamic Social Finance**; how influential are the risks below in the integration of Islamic Social Finance?
   a. There is mismanagement in the implementation of the functions of Islamic social financial institutions
   b. Islamic social finance institutions have not adopted all the principles in governance
   c. There is potential for miscommunication both internally and externally by Islamic social financial institutions
   d. Mis-Coordination related to the division of tasks and roles
   e. Differences in work patterns and culture between institutions
   f. Incompatibilities of the supervisory board
   g. Supervision is not carried out thoroughly
   h. Reports that are not representative and less accurate
   i. There is potential for non-compliance with regulations, both formal rules and sharia rules in Islamic social finance governance
   j. The achievement of several SDGs points is contradictory to the essence of the function of Islamic social finance institutions
   k. The 17 goals in the SDGs are not all relevant to the ZISWAF goals

2. The following are several **risks from the sustainable financing aspect in the integration of Islamic Social Finance**, how influential are the risks below in integrating Islamic Social Finance?
   a. There is liquidity risk for financial institutions

b. The absence of guarantees creates a moral hazard problem
c. Lack of motivation of recipients of Islamic social finance to develop
d. Failure in carrying out financing
e. Time is needed in conditioning the synchronization between ziswaf institutions and the government regarding the legality of the law
f. Literacy related to Islamic social funds is still far from expected
g. High refusal to use waqf funds to lend

3. The following are several **risks from the institutional aspect in integrating Islamic Social Finance**; how influential are the risks below in integrating Islamic Social Finance?
   a. The institutional model is not flexible in accommodating problems
   b. Institutional fund management will limit the space for innovation.

4. The following are several **risks from the HR aspect in institutions in the integration of Islamic Social Finance**, how influential are the risks below in the integration of Islamic Social Finance?
   a. High employee turnover rate
   b. The profession of nazhir and/or amil is not considered as the primary profession
   c. Increasing digitalization will replace the role and number of HR

5. The following are several **risks from regulatory aspects in integrating Islamic Social Finance**; how influential are the risks below in the integration of Islamic Social Finance?
   a. There are no mandatory rules
   b. There is a period for regulatory amendments, so regulations are less flexible in responding to current conditions
   c. There are political challenges in strengthening regulations
   d. Change of leader replaces policy direction
   e. Conflicts of interest that arise between policymakers

6. The following are several **risks from using technology in the integration of Islamic Social Finance**; how influential are the risks below in the integration of Islamic Social Finance?
   a. There is a crime in the implementation of technology
   b. There is a potential for non-fulfillment of sharia principles with the use of technology

SECTION 3: SOLUTION AND STRATEGIES IN INTEGRATING ISLAMIC SOCIAL FINANCE

1. The following are several **solutions in integrating Islamic Social Finance**; how important are the solutions below in the integration of Islamic Social Finance?
   a. Financial integration in providing funding to beneficiaries through the distribution of zakat, waqf, and Islamic microfinance based on the beneficiaries' economic circumstances and capabilities
   b. Integrating ISF with state fiscal instruments, which is supported by existing regulations
   c. Integration through non-formal coordination between ISF institutions
   d. Integration among Islamic social finance institutions based on capacity building and role division
   e. Establishing an integrated national data center for all ISF instruments
   f. Integration based on regional clusters with a solid authority
   g. Integrating ISF through the passage of new laws and regulations
   h. Integration based on ISF management clusters, such as zakat *Amils* joining other zakat institutions and waqf manager joining other waqf institutions

2. The following are several **strategies in integrating Islamic Social Finance**; how important are the strategies below in the integration of Islamic Social Finance?
   a. Improve the quality of human resources through training and certification
   b. Promotes the availability of real-time data through database integration and reporting
   c. Optimizing the digital technology in Islamic social finance instruments collection, management, and distribution process
   d. Coordination and synergy among stakeholders (e.g., practitioners, regulators, associations, and academicians) to establish shared commitment and vision
   e. Encouraging the broadening of the range of benefits by establishing service units at lower levels, such as service units at the district, village, and mosque levels, as well as strategic locations that support the broadening of the reach of the benefits of ISF instruments
   f. Establishing a new institution that is capable of integrating all instruments of ISF
   g. Realizing the government's role in supporting the integration of ISF through regulation and socialization
   h. Developing the ISF institutions to have two functions, namely, *amil* and *nazir*
   i. Revising and updating all laws and regulations governing the management of Islamic social finance instruments to accommodate the development of ISF instruments

Integrating Sustainable Islamic Social Finance: An Analytical Network Process using the Benefit Opportunity Cost Risk (ANP BOCR) Framework: The Case of Indonesia

Tika Widiastuti[1], Anidah Robani[2*], Puji Sucia Sukmaningrum[3], Imron Mawardi[4], Sri Ningsih[5], Sri Herianingrum[6], Muhammad Ubaidillah Al-Mustofa[7]

[1,3,4,6,7]Department of Islamic Economic, Faculty of Economic and Business, Universitas Airlangga
[2]Institute of Technology Management and Entrepreneurship, Universiti Teknikal Malaysia Melaka (UTeM)
[5]Department of Accounting, Faculty of Economic and Business, Universitas Airlangga
~~[2]Institute of Technology Management and Entrepreneurship, Universiti Teknikal Malaysia Melaka (UTeM)~~
~~Email: [1*]tika.widiastuti@feb.unair.ac.id,~~
*Corresponding author
Email: anidah@utem.edu.my, ~~[3]puji.sucia@feb.unair.ac.id, [3]ronmawardi@feb.unair.ac.id, [5]sri.ningsih@feb.unair.ac.id, [6]sri.herianingrum@feb.unair.ac.id, [7]ubaid9339902@gmail.com~~

Abstract

The utilization of Islamic social finance instruments is far behind what is expected. To realize its full potential, Islamic social finance instruments must be integrated. This study examined solutions and priority strategies for integrating sustainable Islamic social finance that could be implemented in the short and long term using the Benefit, Opportunity, Cost, and Risk (BOCR) framework, which includes six aspects: Governance, sustainable financing, institutional aspect, human resources, regulations, and supporting technology. This qualitative research employed the Analytic Network Process (ANP) method using the benefit, opportunity, cost, and risk analysis. The data were obtained mainly through focus group discussions and in-depth interviews with respondents among academics, practitioners, associations, regulators, and community leaders. The respondents were selected for their expertise and experience in the selected topic. The data were processed using the Microsoft Excel and Super Decision software. There are several findings worth considering from the analysis. First, the highest priority in integrating Islamic social finance aspects are human resources (0.97), regulation (0.86), and technology (0.76). Second, based on the short- and long-term analysis, financial integration through sustainable financing (0.01 and 1.44, respectively) and improving human resource quality through certification and training (0.01 and 1.56, respectively) is a priority solution and strategy to integrate sustainable Islamic social finance. Meanwhile, according to expert judgments, integrating national data (0.24) and optimizing technology use (0.18) are priority solutions and strategies. The findings emphasize the critical role of improving human resource quality to utilize technology, with experts identifying a national data integration as the most critical solution. As a result, relevant stakeholders are concerned about technology management training for Islamic philanthropic managers, with the goal of maximizing the potential of technology's growing role and adoption. ~~**Purpose** – utilization sfsfdsfGThis research analyzes solutions and priority strategies for integrating sustainable Islamic Social Finance that could be implemented in the short and long term by analyzing it through the Benefit, Opportunity, Cost, and Risk (BOCR) framework, which comprises of six aspects: Governance, Sustainable Financing, philanthropic Institutions, Human Resources, Regulations, and Technology.~~

~~**Design/methodology/approach** – This qualitative research employeds the Analytic Network Process (ANP) method using the bBenefit, oOpportunity, cCost, and rRisk analysis. The dData were obtained~~

Style Definition: Heading 1: Font: Bold, Font color: Text 1
Style Definition: Heading 2: Font: Bold
Formatted: Justified
Formatted: Justified
Formatted: Justified
Formatted: Justified
Formatted: Font: (Default) +Headings CS (Times New Roman)
Formatted: Font: +Headings CS (Times New Roman)
Formatted: Heading 1, Left
Formatted: Not Highlight
Formatted: Not Highlight
Formatted: Not Highlight
Formatted: Not Highlight
Formatted: Not Highlight
Formatted: Not Highlight
Formatted: Not Highlight
Formatted: Not Highlight
Formatted: Not Highlight
Formatted: Not Highlight
Formatted: Not Highlight
Formatted: Not Highlight
Formatted: Not Highlight
Formatted: Not Highlight
Formatted: Not Highlight
Formatted: Not Highlight
Formatted: Font: (Default) +Headings CS (Times New Roman)
Formatted: Font: (Default) +Headings CS (Times New Roman), Not Highlight
Formatted: Not Highlight
Formatted: Not Highlight
Formatted: Not Highlight
Formatted: Font: (Default) Times New Roman
Formatted: Font: (Default) Times New Roman

mainly through fFfocus gGgroup dDdiscussions and in-depth interviews with experts fromamong academics, practitioners, associations, regulators, and community leaders. weforthe The data wereis processed with using the Microsoft Eexcel and Super Decision software. from the analysisin the , respectively, respectivelysfaresiesrolea ’edfor BOCR(BOCR) Consequently, the solutions and strategies developed will be more integrative and sustainable in the future for the advancement of Islamic sSocial fFinance.

Findings – There are several findings worth considering. First, the highest priority integrating Islamic social finance aspects are human resources (0.97), regulation (0.86), and technology (0.76). Second, based on a short and long-term analysis, financial integration through sustainable financing (0.01 and 1.44) and improving human resource quality through certification and training (0.01 and 1.56) is a priority solution and strategy to integrate sustainable Islamic Social Finance. Meanwhile, according to expert judgments, integrating national data (0.24) and optimizing technology use (0.18) is a priority solution and strategy.

Practical implication – The findings emphasize the critical importance of improving the quality of technology-based human resources, with experts identifying national data integration as the most critical solution. As a result, relevant stakeholders are concerned about technology management training for Islamic philanthropic managers, with the goal of maximizing the potential of technology's growing role and adoption.

Originality/value – This study develops solutions and strategies for short and long-term Islamic social finance integration by identifying the benefit, opportunity, cost, and risk of six related aspects. As a result, the solutions and strategies developed will be more integrative and sustainable for the betterment of Islamic Social Finance in the near future.

Keywords: Financial Sustainability, Integration, Sustainable Islamic Social Finance, ANP BOCR, Priority Strategy

Paper Type Research paper

Formatted: Not Highlight

Formatted: Not Highlight

Formatted: Not Highlight

Formatted: Not Highlight

Formatted: Not Highlight

Formatted: Space After: 10 pt

Formatted: Font: (Default) +Headings CS (Times New Roman), 11 pt, Not Highlight

Formatted: Justified

Formatted: Font: (Default) +Headings CS (Times New Roman)

Formatted: Left

Formatted: Font: (Default) Times New Roman

Formatted: Font: (Default) Times New Roman

IntroductionNTRODUCTION

Islamic social SsSocial finance fFinance (ISF), which includes zakat, *infaq*, *sadaqah*, waqf, and Islamic microfinance, is critical to promoting a long-term economic development [1–3]. (Ibrahim & Ghazali, 2014; Zauro et al., 2020; Widiastuti et al., 2021). Islamic social finance has a vital role in solving the problem of poverty [(Abdullah, 2018; Ascarya, 2021; Robani and Salih, 2018; Widiastuti, Auwalin, *et al.*, 20213-6]), income inequality [7], global education [8,9], health access [10]-, and various other socio-economic problems. The role of Islamic social finance instruments is not only in the majority Muslim countries, but also in minority Muslim countries, such as the role of zakat in providing supportive programs for the poor in Singapore [11] , the role of Islamic social finance into support -economic resilience in the UK [12], and the role of zakat in creating prosperity in Germany, France, and Croatia [13].

-Islamic social finance has also shown a great promise in addressing socioeconomic issues induring , such as the Covid-19 pandemic. -The Covid-19 pandemic has had a significant impact, especially in the economic field [14]. The economic implications of Covid-19, particularly in Indonesia [15,16] (Caraka et al., 2020; Suryahadi et al., 2020; World Bank, 2020), include increasing unemployment and underemployment rates, declining income levels, economic contraction, slow and uneven recovery process across sectors, including the health, economic, and education sectors. This is due to the limited sources of funding owned by the state. The government has prepared a reserve budget of USD 5.3 billion and a budget for the National Economic Recovery program of USD 37.3 billion. The funding wais used for the health sector (USD 1.0 billion), social protection (USD 2.5 billion), and support for mMicro, sSmall, and mMedium eEnterprises (USD 1.8 billioon) [17]. The sSources of funds weare derived from debt, government bonds, Sukuk, and foreign debt. The Ministry of Finance [18] Ministry of Finance (2020) released the funding components of Covid-19 originating from mMarket fFinancing, bBilateral and mMultilateral dDebts, pPrivate placement, fFinancing from oOver bBudget bBalance (SAL), and the use of gGovernment eEndowment fFunds. These components certainly have limits to meet the growing needs of handling Covid-19. Consequently, the government needs to find alternative sources of funding. Islamic social finance with a great potential can be utilized to lessen the burden of funding and accelerate the recovery from the impact of the Covid-19 pandemic.

Indonesia, as a Muslim majority country, has a high potential for Islamic sSocial fFinance. The potential for collecting zakat funds in Indonesia in 2020 wais USD 23 billion [19] (19), cash waqf wais USD 12.6 billion per year, and the overall value of waqf land is USD 140 billion [18] (20). However, the collection of zakat and waqf funds is still far from its full potential. The zakat collection in Indonesia in 2019 was only USD 0.7 billion [19] (19), while the cash waqf funds collected were only USD 0.2 billion [18] (20). One of the reasons for the vast gap between the potential realization and potential of Islamic social financeISF is a lack of optimal governance [20]. (Widiastuti, Cahyono, et al., 2021).

Furthermore, the management of ISF is facesd with severalthe problems, including of the lack of technology utilization in its management [20], k the lack of ability and competence of managers, and the lack of synergy between stakeholders [20,21], and lack of trust and public

Formatted: Font: +Headings CS (Times New Roman)
Formatted ...
Formatted ...
Formatted ...
Formatted ...
Formatted ...
Field Code Changed ...
Formatted ...
Formatted ...
Formatted ...
Formatted ...
Formatted ...
Formatted ...
Formatted ...
Formatted ...
Formatted ...
Formatted ...
Formatted ...
Formatted ...
Formatted ...
Formatted ...
Formatted ...
Formatted ...
Formatted ...
Formatted ...
Formatted ...
Formatted ...
Formatted ...
Formatted ...
Formatted ...
Formatted ...
Formatted ...
Formatted ...
Formatted ...
Formatted ...
Formatted ...
Formatted ...
Formatted ...
Formatted ...
Formatted ...
Formatted ...
Formatted ...
Formatted ...
Formatted ...

literacy [20,22]. Based on a survey conducted by BAZNAS (Zakat Government Institution), nationally, the level of zakat literacy is at 66.78 %, which is in the middle category. ~~However, for several provinces in Indonesia~~Despite ~~being~~ the country ~~with~~having the largest Muslim population, the literacy level for several provinces in Indonesia ~~are~~is ~~is~~ still in the low category, with a score below 50 %. This means that literacy is not at an advanced stage, of which this includes knowledge about the existence and functions of ISF institutions, regulations, impacts, programs, and digital payments [23].

~~According to the law, the~~ The governance of ISF ~~of Islamic social finance~~ includes aspects of collection, management, ~~distribution~~distribution, and utilization of social funds ~~(Law No. 23 of 2011).~~ ~~.~~ The issues of ISF governance can be categorized into six aspects consisting of Human Resources (HR), Unsupported Policies and Regulations~~Regulation~~, ~~,~~ Technology, Sustainable F~~f~~inancing, Governance, and ~~Institutions~~ Institutional Aspect [20,24–26]. ~~(Beik, 2015; Pitchay et al., 2018; Ahmad, 2019; Ali et al., 2020; Mustofa et al., 2020; Sukmana, 2020; Widiastuti et al., 2021).~~ Problems in the HR aspect include the lack of ability and motivation of human resources to develop~~,~~ both ~~human resource~~ managers and recipients of Islamic social funds [20]. ~~(Widiastuti , Cahyono, et al., 2021).~~ In addition, ~~human resources in~~ Islamic philanthropic institutions are dominated by employees who are not from the background of Islamic Economics and ~~ZISWAF~~ Zakat Waqf management, causing the low ~~ability~~ productivity ~~of HR~~ to manage ZISWAF. In addition, the low literacy related to Islamic social funds needs to be addressed by policymakers [27]. ~~(Ali et al., 2020).~~

Unsupported policies and regulations become issues from the regulatory aspect [20] ~~(Mustofa et al., 2020; Widiastuti , Cahyono, et al., 2021)~~. First, zakat ~~and waqf regulation i~~is still a voluntary act [20] ~~In Indonesia, zakat regulation is still voluntary,~~ and ~~. Second,~~ there is~~are~~ no sanctions for those who do not carry out zakat [28]. ~~Third~~Further, ~~,~~ the laws that govern ISF are ~~still~~ separated as ~~. Zakat and waqf are governed by different laws;~~ zakat is governed by Law No. 23 of 2011, whereas waqf is governed by Law No. 41 of 2004 ~~Furthermore, zakat and waqf are governed by different laws; zakat is governed by Law No. 23 of 2011, whereas waqf is governed by Law No. 41 of 2004~~.

In addition, ~~Fourth,~~ ~~Furthermore,~~ ~~t~~the infrastructure required for management ~~is incomplete~~needed to be addressed, particularly in the field of technology [20]. This becomes an issue that deserves consideration [29]. ~~(35).~~ Many institutions do not have the required financial capacity to purchase the infrastructure needed ~~for~~ to ~~technology~~support technology-based governance.

Problems related to the sustainability aspects of the ISF program are of concern to IRTI [29], Pitchay et al. [24], and Sukmana [26]. ~~IRTI (2014), Pitchay et al. (2018), and Sukmana (2020).~~ Based on the findings in the field ~~Berdasarkan temuan di lapangan~~, m~~M~~ost empowerment programs using Islamic social funds do not consider sustainability aspects and seem to end when the program ends, so that the primary goal of empowerment, namely, the transformation of *mustahiq* into *muzakki*, or *mauquf '_alaihi* into *wakif*, cannot be achieved. In addition, in the aspect of governance, Islamic social fund instruments are still partially managed. These

Formatted
Formatted
Formatted
Formatted: Font: (Default) +Headings CS (Times New Roman), English (United States), Do not check spelling or grammar
Formatted: Font: (Default) +Headings CS (Times New Roman), English (United States), Do not check spelling or grammar
Formatted: Font: (Default) +Headings CS (Times New Roman), English (United States), Do not check spelling or grammar
Formatted: Font: (Default) +Headings CS (Times New Roman), English (United States), Do not check spelling or grammar
Formatted: Font: (Default) +Headings CS (Times New Roman), English (United States), Do not check spelling or grammar
Formatted: Font: (Default) +Headings CS (Times New Roman), English (United States), Do not check spelling or grammar
Formatted: Font: (Default) +Headings CS (Times New Roman), English (United States), Do not check spelling or grammar
Formatted: Font: (Default) +Headings CS (Times New Roman), English (United States)
Formatted: Font: (Default) +Headings CS (Times New Roman), English (United States)
Formatted: Not Highlight
Formatted: Hyperlink, Font: (Default) +Body (Calibri), 11 pt, Indonesian, Not Highlight
Field Code Changed
Formatted
Formatted
Formatted
Formatted
Formatted
Formatted
Formatted
Formatted: English (United States)
Formatted
Formatted: Font: (Default) +Headings CS (Times New Roman)
Formatted: Font: (Default) +Headings CS (Times New Roman)
Formatted
Formatted

instruments have not been integrated with the State's fiscal policy [30]. (30).This is because Indonesia is based on "Pancasila", not a country withbased on Islamic law, of whichso its population consists of various beliefs and religions. There could be a sense of injustice if the government only regulates ands takes money from Muslims to help government finances. On the institutional aspect, there is a lack of coordination and synergy between stakeholders [20] (Widiastuti , Cahyono, et al., 2021). The lack of public trust in Islamic social finance managers [25] (28) needs to be an essential concern for policymakers. To overcome these problems, the solution that can be done is to formulate the integration of the Islamic social finance sector and establish a sustainable management ecosystem. To overcome these problems, the solution that can be done is to formulate the integration of the Islamic social finance sector and establish a sustainable management ecosystem.

To overcome these problems, the solution that can be done is to formulate the integration of the Islamic social finance sector and establish a sustainable management ecosystem.

The purpose of this research waіs to develop a strategy for the short- and long-term integration of ISF Islamic social finance and to determine the best way to achieve that goal. This study was carried out in Indonesia, a country with a high potential for accumulating ISF Islamic social funds. In practice, however, the ISF Islamic social funds are only partially managed and have not been fully integrated. Raimi et al. [31], Jouti [32], and Shalleh et al. [33] Raimi et al. (2014), Haneef et al. (2015), Jouti (2019), and Shalleh et al. (2020) conducted research on ISF integration. Previous research formulated the integration of ISF Islamic social funds without emphasizing sustainability,; and thus, the sustainability aspect in integrating Islamic social funds iwas highlighted in this research. Furthermore, previous studies employed a qualitative approach through case studies, literature reviews, content and thematic analysis, and a quantitative approach through Structural Equation Modeling (SEM). There has been no research that incorporates ISF Islamic sSocial fFunds that employ the two-level ANP method. The analysis of Benefit, Opportunity, Cost, and Risk (BOCR) to build the ANP framework is what makes this research unique. Several factors influenced the decision to use this method. To begin, the ANP method waіs used to determine the best integration in the long-term management of ISFIslamic social finance. Second, the analysis ofze the benefits, opportunities, costs, and risks of each aspect was to produce findings that can be implemented in the short and long term. Third, this study identifieds priority solutions and strategies for achieving long-term ISF integration using the ANP BOCR method.

The findings show that the financing integration model is a priority solution in the short and long term. This demonstrates that the ISF serves several functions: Consumptive zakat is used to provide emergency assistance in the short term, productive zakat and benevolence funds are used to support economic recovery in the medium term, and waqf or sukuk are used to build long-term resilience. Furthermore, the findings of this study highlight the importance of improving the quality of HR managers through a technology-based certification and training

Formatted: English (United States)
Formatted: Font: (Default) +Headings CS (Times New Roman)
Formatted: English (United States)
Formatted: Not Highlight
Formatted: English (United States)
Formatted: Not Highlight
Formatted: English (United States)
Formatted: Not Highlight
Formatted: English (United States)
Formatted: Not Highlight
Formatted: English (United States)
Formatted: Not Highlight
Formatted: English (United States)
Formatted: English (United States)
Formatted: English (United States)
Formatted: Font: (Default) +Headings CS (Times New Roman)
Formatted: English (United States)
Formatted: Font: (Default) +Headings CS (Times New Roman)
Formatted: Not Highlight
Formatted: Indonesian
Formatted: Font: (Default) +Headings CS (Times New Roman)
Formatted: List Paragraph,Body of text,Daftar Acuan, Indent: Left: 0", Right: 0", Space Before: 0 pt
Formatted: Normal, Left
Formatted: Font: (Default) +Headings CS (Times New Roman), English (United States)
Formatted: Font: (Default) +Headings CS (Times New Roman), English (United States)
Formatted: Font: (Default) +Headings CS (Times New Roman), English (United States)
Formatted: Font: (Default) +Headings CS (Times New Roman), English (United States)
Formatted: Not Highlight
Formatted: Not Highlight
Formatted: Not Highlight
Formatted: Not Highlight
Formatted: Not Highlight
Formatted: Font: (Default) Times New Roman
Formatted: Font: (Default) Times New Roman

(particularly for data analysis, e-marketing and big database management) as the best short- and long-term strategy for implementing Islamic social finance in a sustainable manner. ~~The findings show that the financing integration model is a priority solution in the short and long term. This demonstrates that the ISF serves several functions: Cconsumptive zakat is used to provide emergency assistance in the short term, productive zakat and benevolence funds are used to support economic recovery in the medium term, and waqf or sukuk are used to build long-term resilience. Furthermore, the findings of this study highlight the importance of improving the quality of HR managers through a technology-based certification and training (particularly for data analysis and big database management) as the best short- and long-term strategy for implementing Islamic social finance in a sustainable manner.~~

The findings of this study are critical for several reasons. First, solutions and strategies for the integration of sustainable ISF are expected to solve various problems of managing different instruments of ISF, resulting in the formation of a sustainable ~~Islamic social finance~~ISF ecosystem. Second, the findings of this study ~~will~~ serve as a resource for the ISF institutions in developing long-term strategic plans. Third, the results can provide a broader impact on people's well-being. Fourth, in the context of the Covid-19 pandemic, the findings can provide an overview of strategic policy steps and guidelines for seeking alternative funding sources by leveraging the potential of sustainable ISF~~Islamic social funds~~.

Literature r~~R~~eview

~~2.1~~ The c~~C~~oncept o~~O~~f I~~i~~slamic ~~social~~ s~~S~~ocial ~~finance~~ f~~F~~inance a~~A~~nd i~~I~~ts ~~instruments~~ i~~I~~nstruments

Islamic s~~S~~ocial f~~F~~inance is a financial system that includes traditional and contemporary ISF ~~Islamic social finance~~ instruments [32,34–36]. ~~(IRTI, 2020).~~ Traditional ISF ~~Islamic sSocial fFinance~~ can be divided into two types: P~~p~~hilanthropy and cooperation. Zakat, *infaq*, *sadaqah*, and waqf are traditional ISF ~~Islamic social finance~~ instruments based on philanthropy, while ~~. Meanwhile, the traditional Islamic social finance is based on collaboration that includes~~ *qard* and *kafalah* are based on cooperation. Furthermore, Islamic ~~microfina~~microfinance ~~nce~~ can be categorized as contemporary ~~Islamic social finance~~ISF. Islamic s~~S~~ocial f~~F~~inance contributes to the third sector of the economy by assisting in creating more economic activities and reducing unemployment and poverty [37]. ~~(Hamed, 2020).~~ Several previous studies have revealed the differences in the instruments used in Islamic s~~S~~ocial f~~F~~inance~~,~~ as shown in T~~T~~able 1.~~:~~

Table 1. Instruments of Islamic Social Finance

No	Researchers	Year	Zakat	Infaq	Sadaqah	Waqf	Qard	Qard Hasan	Kafalah	Gift	Islamic Microfinance
~~1~~	~~Sulistyowati~~	~~2018~~	~~✓~~	~~✓~~		~~✓~~					
1. ~~2~~	Jouti~~Jouti~~	2019	✓		✓	✓	✓		✓		✓

Formatted: Not Highlight
Formatted: Not Highlight
Formatted: Heading 1, Left
Formatted: Font: +Headings CS (Times New Roman)
Formatted: Font: Times New Roman
Formatted: Font: Times New Roman
Formatted: Heading 2, Left, No bullets or numbering
Formatted: Font: Times New Roman
Formatted: Font: Times New Roman
Formatted: Font: Times New Roman
Formatted: Heading 2, Left
Formatted: Not Highlight
Formatted: Not Highlight
Formatted: Not Highlight
Formatted: Not Highlight
Formatted: Not Highlight
Formatted: Font: (Default) +Headings CS (Times New Roman), English (United States)
Formatted: Font: Italic
Formatted: Font: Not Italic
Formatted: Not Highlight
Formatted: Not Highlight
Formatted: Not Highlight
Formatted: Not Highlight
Formatted: Hyperlink, Font: (Default) +Body (Calibri), 11 pt, Indonesian, Not Highlight
Formatted: Not Highlight
Formatted: Default Paragraph Font, Font: (Default) +Body (Calibri), 11 pt, Indonesian
Formatted: No underline
Formatted: No underline
Formatted: Centered
Formatted Table
Formatted: Font: (Default) +Headings CS (Times New Roman)
Formatted: Font: (Default) +Headings CS (Times New Roman)
Formatted: Font: (Default) +Headings CS (Times New Roman)
Formatted: Font: (Default) +Headings CS (Times New Roman)
Formatted: Font: (Default) +Headings CS (Times New Roman)
Formatted: Font: (Default) +Headings CS (Times New Roman)
Formatted ...
Formatted ...
Formatted: Font: (Default) Times New Roman
Formatted: Font: (Default) Times New Roman

2. ~~3~~	Usman et al.~~Usman et al.~~	2019	✓	✓	✓						
~~4~~	~~Kamaruddin and Auzair~~	~~2019~~	~~✓~~		~~✓~~	~~✓~~		~~✓~~		~~✓~~	
3. ~~5~~	Adjar et al.~~Adjar et al.~~	2020	✓			✓		✓			
~~6~~	~~Arumsari and Rikawati~~	~~2020~~	~~✓~~	~~✓~~	~~✓~~	~~✓~~					
4. ~~7~~	Hudaefi~~Hudaefi~~	2020		✓	✓	✓					

Source: Authors' Compilation (2021)

Most of the previous research mentions that the instruments for ISF ~~Islamic s~~~~Social f~~~~Finance instruments~~ include z~~Z~~akat, *i~~I~~nfaq*, *s~~S~~adaqah*, and w~~W~~aqf [32,38,39] ~~(Sulistyowati, 2018; Jouti, 2019; Kamaruddin & Auzair, 2019; Adjar et al., 2020; Arumsari & Rikawati, 2020; Hudaefi, 2020; Jouti, 2019; Kamaruddin & Auzair, 2019; Sulistyowati, 2018)~~. Therefore, in this study, ~~what is meant by~~ ISF~~Islamic s~~~~Social f~~~~Finance~~ instruments ~~are~~ refer to z~~Z~~akat, *i~~I~~nfaq*, a~~A~~l~~ms~~ and w~~W~~aqf, and Islamic microfinance.

Each of these instruments has different roles and functions but shares the same objective in~~:~~ achieving prosperity [32] ~~(2)~~. The first instrument is z~~Z~~akat, a mandatory economic instrument for every Muslim [40,41] ~~(Kasri, 2017; Kailani & Slama, 2020; Kasri, 2017)~~. Zakat serves as a means for distributing wealth in the society [42] ~~(10)~~ from the rich to the poor to achieve socio-economic justice, increase economic growth, and reduce inequality [43]. ~~(11).~~ The second instrument is *infaq* and *sadaqah*, which are the voluntary gifts by capable people to seek the pleasure of Allah [44], significantly to help the needy. ~~(12).~~ Zakat, *infaq*, and *sadaqah* are economic instruments that support economic growth by increasing the purchasing power of the needy [3,31]. ~~(Raimi et al., 2014; Jermsittiparsert & Sommanawat, 2019; Raimi et al., 2014; Widiastuti et al., 2021).~~

As a third instrument, w~~W~~aqf is a social fund that plays a vital role in the Islamic framework. An asset owner donates and dedicates movable or immovable assets for the benefit of the community in perpetuity or impermanence. Waqf can be used to provide a variety of free community services, such as the construction of mosques, hospitals and healthcare centers, *madrasas*, education centers, and libraries [26]. ~~(16).~~ Waqf is a vital tool in the Islamic economic system used to meet the needs of the poorest communities and promote community economic growth [26]. ~~(17).~~ Furthermore, waqf, zakat, and sadaqah are instruments capable of acting as the national income and transfer payments used for redistributive purposes in an Islamic economy [6].

Islamic microfinance is the fourth instrument of ISF~~Islamic social finance~~. Islamic microfinance serves two purposes: S~~s~~ocial and commercial. As a social institution, it manages zakat, *infaq*, *sadaqah*, and waqf in accordance with the Syariah. ~~While~~ On the other hand, as a commercial institution, it conducts businesses and provides financing for economic development. Islamic microfinance focuses on economic development through capital assistance and the empowerment of the marginal community with no access to capital to improve their well-being [45]. ~~(Haneef et al., 2013).~~

2.2 Sustainability of I~~i~~slamic s~~S~~social ~~finance~~f~~F~~inance

Sustainability refers to the capacity to operate over a long period and continuously [46]. ~~(Pischke, 1996).~~ Sustainable growth has become a global –development goal in both government and non-governmental organizations [47]. There are two paradigms in sustainability, namely, the institutionalist and welfarist [48]. ~~(21).~~ The institutionalist paradigm considers that financial self-sufficiency is the key to achieving sustainability. Thus, a person can be said to be sustainable if he or she achieves financial independence [48]. ~~(21).~~ In contrast, the welfarist paradigm considers that social functions in poverty alleviation are more important in achieving sustainability. Both paradigms are essential in the context of ISF~~Islamic sSocial fFinance~~. Financial self-sufficiency refers to an institution's ability to fund its operations independently, while also benefiting others. On the other hand, the main goals of ISF ~~Islamic sSocial fFinance~~ are poverty alleviation and its reachability [49–51]. ~~(Ali et al., 2015; Ahmed et al., 2017; Rosli et al., 2018; Bin-Nashwan et al., 2020).~~ Three factors contribute to the sustainability of Islamic s~~S~~ocial f~~F~~inance ~~sustainability~~. First, the collection's sustainability is linked to the fact that funds will always be collected from the community. To ensure the long-term viability of ~~supply~~ funds supply, all information about zakat and its administration must be transparent and easily accessible to the public [52]. ~~(26).~~ Second, institutional sustainability is linked to the institution's ability and availability to carry out operational tasks [48]. ~~(27).~~ Third, the sustainability of distribution is linked to how the Islamic s~~S~~ocial f~~F~~inance i~~I~~nstitution performs its social and intermediary functions to improve welfare and achieve broader benefits [50]. ~~(Ahmed et al., 2017; Bassem et al., 2019).~~

~~In practice, Islamic Social Finance institutions' empowerment programs have not been able to transform the poor into prosperous. Furthermore, many community empowerment programs end after a certain period of time and do not track whether the poor are truly independent and prosperous. Several reasons contribute to the unsustainable nature of empowerment projects carried out by Islamic Social Finance institutions. First, huge sums of money are required to give effective and optimal empowerment (Widiastuti et al., 2018). Third, qualified human resources that have leadership spririt are required (Nguyen-Van et al., 2021). Fourth, it takes the beneficiary's desire and ability to develop and change into a beneficiary (Afriadi & Sanrego, 2016; Widiastuti , Cahyono, et al., 2021).~~

In practice, the empowerment programs of ISF ~~Islamic sSocial fFinance~~ institutions have not been able to transform the poor into prosperous. Furthermore, many of these programs come to an end after a certain period and do not track whether the poor ~~are~~have truly become self-sufficient and prosperous. Several factors contribute to the unsustainable nature of ISF ~~Islamic sSocial fFinance~~ institutions' empowerment projects [20]. To begin, massive sums of money are required to provide effective and optimal empowerment. ~~(Widiastuti et al., 2018).~~ Second, qualified human resources with leadership qualities are needed [53]. ~~(Nguyen-Van et al., 2021).~~ Third, the beneficiary's desire and ability to develop and change into *muzakki* or *munfiq* are required [3,54]. ~~(Afriadi & Sanrego, 2016; Widiastuti et al., 2021).~~

Formatted: Font: +Headings CS (Times New Roman)
Formatted ...
Formatted: Heading 2, Left, No bullets or numbering
Formatted: Font: (Default) +Headings CS (Times New Roman), English (United States)
Formatted ...
Formatted: Font: (Default) +Headings CS (Times New Roman), English (United States)
Formatted: Font: (Default) +Headings CS (Times New Roman), English (United States)
Formatted: Font: (Default) +Headings CS (Times New Roman), English (United States)
Formatted: Font: (Default) +Headings CS (Times New Roman), English (United States), Do not check spelling or grammar
Formatted: Font: (Default) +Headings CS (Times New Roman), English (United States), Do not check spelling or grammar
Formatted: Font: (Default) +Headings CS (Times New Roman), English (United States), Do not check spelling or grammar
Formatted: Font: (Default) +Headings CS (Times New Roman), English (United States), Do not check spelling or grammar
Formatted: Not Highlight
Formatted ...
Formatted: Not Highlight
Formatted ...
Formatted: Font: (Default) +Headings CS (Times New Roman), English (United States), Do not check spelling or grammar
Formatted: Font: (Default) +Headings CS (Times New Roman), English (United States), Do not check spelling or grammar
Formatted: English (United States), Not Highlight
Formatted ...
Formatted ...
Formatted ...
Formatted ...
Formatted: Not Highlight
Formatted ...

~~2.3~~ Issues with I~~i~~slamic s~~S~~ocial ~~finance~~ f~~F~~inance ~~integration~~i~~I~~ntegration

In order to achieve optimal management, ISF ~~Islamic sSocial fFinance~~ must overcome several challenges. A lack of motivation among beneficiaries to develop, a lack of synergy and coordination among stakeholders, a lack of human resources capabilities and quantity to manages Islamic social finance, a lack of ability to optimize technology, regulations that are still voluntary, and a lack of awareness and trust of *muzakki* in Islamic s~~S~~ocial f~~F~~inance institutions are among the problems encountered by Islamic s~~S~~ocial f~~F~~inance [20,24–26,55] ~~(IRTI, 2014; Pitchay et al., 2018; Ahmad, 2019; Ali et al., 2020; IRTI, 2014; Mustofa et al., 2020; Pitchay et al., 2018; Sukmana, 2020; Widiastuti et al., 2021)~~. Further, the ISF~~Islamic social finance~~ instruments are still partially (separatedly) -managed~~. . The following~~, which is ~~points~~ demonstrated by several points~~ that Islamic social finance instruments are still partially managed~~. First, according to the 2017-2022 Indonesian Zakat Architecture, zakat management is supported by regulations but remains a voluntary instrument, not an obligatory instrument [20]. –Second, ISF instruments are governed by different regulations and laws. The management of zakat is regulated in Law no. 23 of 2011, while the management of waqf is regulated in Law no. 41 of 2004. ~~Third, because~~ Further, ISF instruments are managed by various institutions (government, corporate and community organizations~~social community~~) [56] of which there exist differences in the rights, obligations, and interests of different institutions. Fourth, the regulators of zakat and waqf are not the same. The National *Amil* Zakat Agency (BAZNAS) regulates zakat, while the Indonesian Waqf Board (BWI) is the waqf management regulator. However, BAZNAS and BWI a~~were~~ eventually accountable to the Minister of Religion Affairs. Based on these challenges, the integration of ISF must be carried out.~~First, according to the 2017-2022 Indonesian Zakat Architecture, zakat management is supported by regulations but remains a voluntary instrument. Second, Islamic social fund instruments are governed by different regulations and laws. Third, because Islamic social fund instruments are managed by various institutions (government, corporate and social community), there exist differences in the rights, obligations, and interests of different institutions. Fourth, the regulators of zakat and waqf are not the same. The National *Amil* Zakat Agency (BAZNAS) regulates zakat, while the Indonesian Waqf Board (BWI) is the waqf management regulator. However, BAZNAS and BWI were eventually accountable to the Minister of Religion Affairs.~~

~~Given the obstacles that result in a substantial gap between the potential and actualization of Islamic social finance management, it is vital to integrate Islamic Social Finance instruments on a continuous basis to attain an optimum level of management.Given the challenges which consequently create a significant gap between the potential and actualization of Islamic social finance management and accounting for the enormous potential of Islamic social finance, it is necessary to integrate Islamic Social Finance instruments on an ongoing basis to achieve optimum level of management.~~

Formatted: Font: +Headings CS (Times New Roman)
Formatted: Heading 2, Left, No bullets or numbering
Formatted: Font: +Headings CS (Times New Roman)
Formatted: Font: +Headings CS (Times New Roman)
Formatted: Font: +Headings CS (Times New Roman)
Formatted: Font: Italic
Formatted: English (United States)
Formatted: English (United States)
Formatted: English (United States)
Formatted: Not Highlight
Formatted: English (United States)
Formatted: Not Highlight
Formatted: English (United States)
Formatted: Not Highlight
Formatted: English (United States)
Formatted: Not Highlight
Formatted: English (United States)
Formatted: Not Highlight
Formatted: English (United States), Not Highlight
Formatted: Not Highlight
Formatted: Hyperlink, Font: (Default) +Body (Calibri), 11 pt, Not Highlight
Formatted: Not Highlight
Formatted: English (United States)
Formatted: English (United States)
Formatted: Not Highlight
Formatted: English (United States)
Formatted: Not Highlight
Formatted: English (United States)
Formatted: English (United States)
Formatted: Not Highlight
Formatted: English (United States)
Formatted: Not Highlight
Formatted: English (United States)
Formatted: Not Highlight
Formatted ...
Formatted: Not Highlight
Formatted: English (United States)
Formatted: Not Highlight
Formatted: English (United States)
Commented [A1]: Sitasi
Formatted ...
Formatted: English (United States)
Formatted: English (United States)
Formatted: Font: (Default) Times New Roman
Formatted: Font: (Default) Times New Roman

~~2.4~~ Integration model of I~~i~~slamic social finance

Integration refers to the process of blending into a single unit, thus, integration in ISF ~~Islamic social finance~~ combines funds management, institutions, governance, functions, etc. According to Balassa [57], integration offers various advantages. First, integration allows for the mobility of production elements, which has a positive impact on enhancing welfare. Second, integration ~~has the ability to~~ reduces costs, resulting in an increase in output. Third, integration promotes job specialization. Fourth, integration~~the~~ creates~~ion~~ a~~of~~ welfare through policy harmonization. Fifth, integration can increase technology adoption. Furthermore, integration has a favorable impact on the socio-economic aspects of the community, including: (1) I~~i~~mproving sustainability performance [58], ~~;~~(2) enhancing economic growth~~—~~ [59], ~~;~~and (3) improving socio-economic fairness [2].

Raimi et al. [31] ~~Raimi et al. (2014) incorporated~~ integrated c~~C~~orporate s~~S~~ocial r~~R~~esponsibility, z~~Z~~akat, and w~~W~~aqf into the f~~F~~aith-b~~B~~ased m~~M~~odel to reduce poverty, promote business development, and strengthen the economy of Muslim-majority communities. Hassanain [60] ~~Hassanain (2015)~~ conducted a literature review on microfinance, waqf, and zakat to develop an integration model. Haneef et al. [45] ~~employeds qualitative methods to construct the integration of zakat and waqf for the disaster management. Haneef et al. (2015)~~ used the Structural Equation Model (SEM) analysis to examine the significant influence between differencct aspects of Islamic social finance that include waqf, takaful, human resource management, and financing~~, etc~~. Pitchay et al. [24] ~~Pitchay et al. (2018)~~ conducted a literature review to develop a cooperative-waqf model. Further, using the thematic analysis, Ambrose et al. [61] ~~Ambrose et al. (2018)~~ developed a waqf financing model for public goods and mixed public goods in Malaysia, where the proceeds of waqf investment can be used to fund 11 items of government spending.

A r~~R~~esearch that specifically discusses the sustainability of the ISF ~~Islamic sSocial fFinance~~ model was carried out by Jouti [32] ~~Jouti (2019)~~, which focuse~~s~~d on creating an ISF ~~Islamic sSocial fFinance~~ ecosystem, and by Shalleh et al. [33] ~~Shalleh et al. (2020),~~ which focuses on the integration of waqf-based takaful for flood victims in Malaysia. This study developed thes previous research by building an integration model between ISF ~~Islamic sSocial fFinance~~ instruments with an emphasis on sustainability aspects using the two-level ANP method.

Method

~~3.1~~ Research method and analysis approach

The Analytical Network Process (ANP) method wa~~i~~s used in this qualitative study, along with the analysis of Benefit, Opportunity, Cost, and Risk (BOCR). This two-~~-~~level~~s~~ ANP method has the characteristics of mathematically examined conditions and inputs obtained based on several ~~criteria~~constructs, allowing it to aid the decision-making from various options and complex data [62]. ~~(43).~~ The ANP method approach was chosen because ISF ~~Islamic social funds~~ include various instruments, each with its own set of characteristics and issues, both

tangible and intangible. In this study, the ANP approach wa~~i~~s the best choice for producing strategic decisions [63]. Meanwhile, the BOCR analysis was selected for a variety of reasons~~.~~: t~~T~~o begin, every decision has both positive and negative consequences, which must be considered when determining priority decisions ~~(44)~~. Second, the BOCR approach wa~~i~~s used to make decisions that can be implemented in the short and long terms. The ANP BOCR method prioritizes solutions and strategies to develop a sustainable ISF ~~Islamic social finance~~ integration model. The ANP method of research has three phases, which are depicted in Fig~~ure~~ure 1 [64]. ~~below (45).:~~

Figure 1. ANP BOCR Research Stage

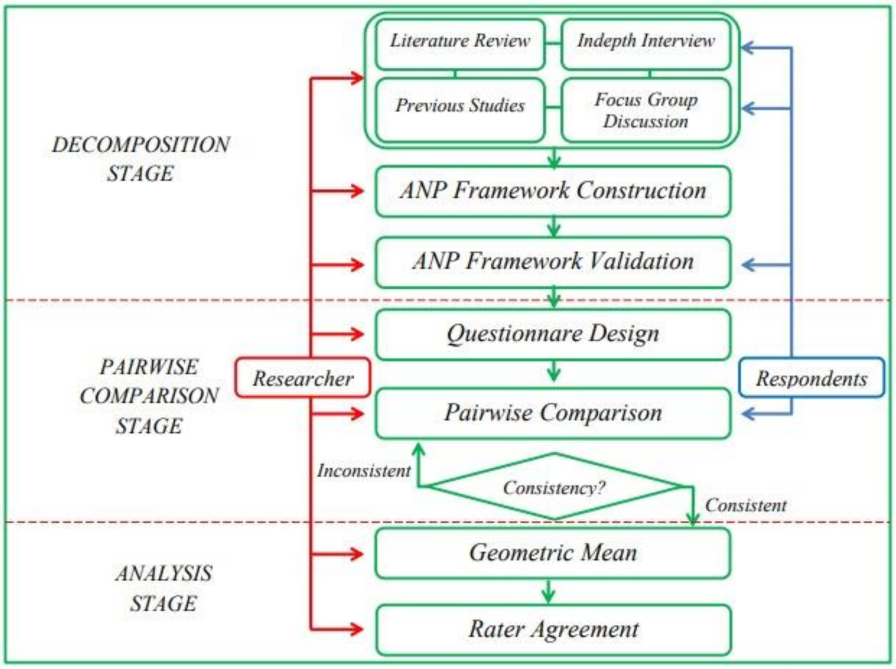

Source: Ascarya [64]~~(45)~~

First, the decomposition phase ~~seeks~~ sought to construct and validate the ANP model through f~~F~~ocus g~~G~~roup d~~D~~iscussions and in-depth interviews with experts in ISF ~~Islamic social finance~~ ~~from~~ among academics, practitioners, associations, regulators, and community leaders. ~~In addition, a~~ A l~~L~~iterature review was conducted as part of this study to strengthen the ANP model that was developed. A literature review analysis wa~~i~~s used to collect problems, solutions, and strategies discovered and analyzed by previous researchers to integrate sustainable ISF. In addition, the literature review analysis was used to validate the findings from the focus groups discussion and in-depth interviews.

Formatted: Not Highlight
Formatted: Not Highlight
Formatted: Font: (Default) +Headings CS (Times New Roman), English (United States)
Formatted: Not Highlight
Formatted: Font: (Default) +Headings CS (Times New Roman), English (United States)
Formatted: Left
Formatted: Font: (Default) +Headings CS (Times New Roman)
Formatted: Not Highlight
Formatted: Not Highlight
Formatted: Not Highlight
Formatted: Not Highlight
Formatted: Not Highlight
Formatted: Not Highlight
Formatted: Not Highlight
Formatted: Not Highlight
Formatted: Not Highlight
Formatted: Not Highlight
Formatted: Not Highlight
Formatted: Not Highlight
Formatted: Font: (Default) Times New Roman
Formatted: Font: (Default) Times New Roman

Three Ffocus Ggroup Ddiscussions (FGDs) were held. The first and second FGDs were held with experts to identify aspects, solutions, and strategies for integrating sustainable ISF, and identify benefits, opportunities, costs, and risks to determine the short-term and long-term analyses. The model developed through FGD 1 and 2 was then strengthened through in-depth interviews and literature reviews. Following that, the third FGD validated the constructed ANP model. The second was the pairwise comparison phase to design a questionnaire in the form of a pairwise comparison to determine and analyze aspects, solutions, and priority strategies in integrating sustainable ISF. The questionnaire that the respondents had answered were used for the next phase. The third was the data analysis phase, which aimed to calculate the priority value of each aspect, solution, and strategy formulated using the Microsoft Excel and Super Decision software. The use of the ANP method with the BOCR approach made it possible to produce realistic and net values. The net value/additive negative describes alternative options that are more profitable or ideal to be prioritized in the long term, while realistic/multiplicative value represents more favorable outcomes for short-term decisionsThree focus group discussions were held. The first and second Ffocus Ggroup Ddiscussions (FGDs) were held with experts to identify aspects, solutions, and strategies for integrating sustainable Islamic social finance and identify benefits, opportunities, costs, and risks to determine the short-term and long-term analyseis. The model developed through FGD 1 and 2 was then strengthened through in-depth interviews and literature reviews. Following that, the third FGD validatedds the constructed ANP model. The sSecond was, the pairwise comparison phase to design a questionnaire in the form of a pairwise comparison to determine and analyze aspects, solutions, and priority strategies in integrating sustainable Islamic social finance. The questionnaire that the respondents hadve answered will bewere used for the next phase. The third wais the data analysis phase, which aimeds to calculate the priority value of each aspect, solution, and strategy formulated using the Microsoft Excel and Super Decision software. The use of the ANP method with the BOCR approach madkes it possible to produce realistic and net values. The net value/additive negative describes alternative options that are more profitable or ideal to be prioritized in the long term, while realistic/multiplicative value represents more favorable outcomes for short-term decisions [63,65] (Saaty & Vargas, 2006; Zammori et al., 2014). According to Saaty and Vargas [63] (Saaty and& Vargas, (2006), there are several steps to synthesize each priority option from each criterion where:

$$\text{Net Value/additive negative} = bB+oO-cC-rR$$

(Equation 1)

$$\text{Realistic/multiplicative} = (BxO)/(CxR)$$

(Equation 2)

B or b stands for Benefit, O or o stand for Opportunity, C or c stands for cost, R or r stands for Risk. After the analysis phase, the next step waiis to validate the results with various ISFIslamic sSocial fFinance experts to strengthen the integration model that will be built.

Formatted: Not Highlight

Formatted: Not Highlight

Formatted: Not Highlight

Formatted: Not Highlight

Formatted: Not Highlight

Formatted: Not Highlight

Formatted: Font: (Default) +Headings CS (Times New Roman)

Formatted: Not Highlight

Formatted: Hyperlink, Font: (Default) +Body (Calibri), 11 pt, Not Highlight

Field Code Changed

Formatted: No underline

Formatted: No underline

Formatted: Justified

Formatted: Font: (Default) Times New Roman

Formatted: Font: (Default) Times New Roman

~~3.2~~ Data and research samples

The data used we~~a~~re primary data obtained through focus groups, in-depth interviews, and ANP questionnaires filled out by the respondents. The data sources gathered were from 13 experts ~~from~~among academics, practitioners, associations, regulators, and community leaders. In ANP research, the number of respondents is not a critical ~~the most important~~ requirement for research validation [66] ~~(Firmansyah and & Sukmana, 2014),~~ but ~~rather~~ their expertise is~~s~~. Each expert completed the questionnaire consciously and in a mentally healthy state. The participants we~~a~~re required to fill in a specific consent form before~~-~~hand to ensure that they we~~a~~re not subject to any pressure from any party to participate in the interviews. The research team guaranteed~~s~~ the confidentiality of the experts' data and ~~answers~~ responses and is fully responsible for all applicable laws. Applicable laws will account for the disclosure of confidential data and experts’ responses.

In terms of research ethics, this research followed~~s~~ the rules and ethics of research conduct and writing coordinated by the Institute for Research and Community Service (LPPM), Universitas Airlangga and those mandated by law. It is also explained in the Airlangga University Chancellor's Regulation Number 34 of 2019 concerning the Rules of Conduct clause 16 (b) that researchers ~~are~~ must be honest, objective, and adhere to all aspects of the research process and may not falsify or manipulate data or research results, as well as clause 16 (f) that researchers must respect and appreciate the object of research, whether in the form of humans or animals, both living and dead, or parts/fragments of the object of research. In addition, this research wa~~i~~s supervised by the Center for Research and Publications (3P) within the Faculty of Economics and Business Universitas Airlangga, which functions as an ethics committee according to the Dean Decree Number 88/UN3.1.4/2020.

The purposive sampling technique aims to select respondents according to the criteria required in the ANP method. For this study, the selected respondents must have the following criteria: (1) R~~r~~espondents are experts in the field of ISF ~~Islamic social finance from~~ among academics, practitioners, associations, regulators, and community leaders,~~;~~ (2) have more than two journal publications in the field of ISF ~~Islamic social finance~~ for academic representatives,~~;~~ and (3) for representatives of regulator, practitioners, and associations, they should hold an important position and bear a great responsibility in the management of ISF ~~Islamic social finance~~ in their respective institutions. The research team ~~will~~ contacted the prospective (representative) respondents by phone and email before conducting the interviews to inquire about their willingness to complete the questionnaire. There we~~a~~re two options for completing the questionnaire: O~~o~~nline or offline. ~~In the case where~~For the respondents who opted to complete the questionnaire offline, the research team ~~will~~ attended to them, while adhering to a strict health protocol, by still maintaining a safe distance and wearing a mask. For respondents who opted to fill out questionnaires via teleconference zoom, they were accompanied by three team members with each job desk either as~~:~~ a moderator, a note-taker, ~~and~~ or a backup. The list of respondents in this study is shown in Table 2.~~:~~

Formatted: Heading 2, Left
Formatted: Font: +Headings CS (Times New Roman)
Formatted: English (United States)
Formatted: English (United States)
Formatted: English (United States)
Formatted: English (United States)
Formatted: Not Highlight
Formatted: English (United States)
Formatted: Not Highlight
Formatted: Hyperlink, Font: (Default) +Body (Calibri), 11 pt, Indonesian, Not Highlight
Formatted: English (United States)
Formatted: Not Highlight
Formatted: English (United States)
Formatted: Not Highlight
Formatted: English (United States)
Formatted: English (United States)
Formatted: English (United States)
Formatted: English (United States)
Formatted: English (United States)
Formatted: English (United States)
Formatted: English (United States)
Formatted: English (United States)
Formatted: English (United States)
Formatted: English (United States)
Formatted: English (United States)
Formatted: Not Highlight
Formatted: Not Highlight
Formatted: Not Highlight
Formatted: English (United States)
Formatted: English (United States)
Formatted: English (United States)
Formatted: English (United States)
Formatted: English (United States)
Formatted: English (United States)
Formatted: English (United States)
Formatted: English (United States)
Formatted: English (United States)
Formatted: English (United States)
Formatted: English (United States)
Formatted: English (United States)
Formatted: English (United States)
Formatted: Font: Bold
Formatted: Font: (Default) Times New Roman
Formatted: Font: (Default) Times New Roman

Table 2. List of Research Respondents

No	Representative	Name	Institution	Affiliation
1	Association	KH	National Zakat Forum in East Java	Leader
2		BS	National Zakat Forum	Leader
3	Practitioner	CW	Zakat Institution "*Manajemen Infak*"	Director of mobilization, research, and transformation of Zakat Institution
4		MHZ	BAZNAS Centre Strategic of Studies	Director
5	Regulator	SPY	Ministry of Religious Affairs	Staff at Ministry of Religious Affairs
6		SEH	National Committee on Islamic Economy and Finance (KNEKS)	Director of Sharia Ecosystem Infrastructure
7		AJ	National Committee on Islamic Economy and Finance (KNEKS)	Director of Sharia Social Finance
8	Academics	NH	Universitas Yarsi	Professor in Islamic Economics
9		RS	Universitas Airlangga	Professor in Islamic Economics
10		MSN	Universitas Indonesia	Head of Sharia Finance Education and Human Resources Development Division
11	Community Leader	MTH	Department of Population and Civil Registration	Secretary
12		MZK	Al Mustofa Educational and Social Foundation	Founder
13		NS	Indonesian Zakat Initiative (IZI)	Program Director

Source: Author's compilation (2021)

The~~is study's~~ questionnaire of the study is in the form of a pairwise comparison of the benefits, opportunities, costs, and risks of related aspects in integrating sustainable ISF~~Islamic social finance~~. Governance, sustainable financing, institutions, regulations, technology, and human resources are among the issues at stake in integrating the instruments of ISF~~Islamic social finance~~. The questionnaire also ~~develops~~ comprises various solutions and strategies to achieve the research objectives. The pairwise comparison assessment in the questionnaire is rated on a scale of 1-9, where 1 (one) means not important/needed/influential and 9 (nine) means absolutely important/needed/influential.

~~4.~~ Result

This study aimeds to integrate sustainable **ISF** by building solutions and priority strategies in the short and long term. Before developing solutions and strategies, identification of important aspects wais carried out to provide a comprehensive analysis of the most appropriate priority solutions and strategies in integrating sustainable **ISF**.

~~4.1~~ Priority aspect

Human resources, regulation, technology, sustainable financing, governance, and institutions are six aspects related to the integration of sustainable ISF~~Islamic social finance~~, according to the FGD and in-depth interviews. Table 3 shows the aspects with the highest priority based on the ANP data processing.

Formatted: Check spelling and grammar
Formatted: Font: (Default) +Headings CS (Times New Roman)
Formatted: Centered
Formatted: Font: (Default) +Headings CS (Times New Roman)
Formatted: Font: (Default) +Headings CS (Times New Roman)
Formatted: Font: (Default) +Headings CS (Times New Roman)
Formatted: Font: (Default) +Headings CS (Times New Roman)
Formatted: Font: (Default) +Headings CS (Times New Roman)
Formatted: Font: (Default) +Headings CS (Times New Roman)
Formatted: Font: (Default) +Headings CS (Times New Roman)
Formatted: Font: (Default) +Headings CS (Times New Roman)
Formatted: Font: (Default) +Headings CS (Times New Roman)
Formatted: Font: (Default) +Headings CS (Times New Roman)
Formatted: Font: (Default) +Headings CS (Times New Roman)
Formatted: Font: (Default) +Headings CS (Times New Roman)
Formatted: Font: (Default) +Headings CS (Times New Roman)
Formatted: Font: (Default) +Headings CS (Times New Roman)
Formatted: Font: (Default) +Headings CS (Times New Roman)
Formatted ...
Formatted ...
Formatted: Left
Formatted: Font: +Headings CS (Times New Roman)
Formatted ...
Formatted: Justified
Formatted: Font: Not Bold, Not Highlight
Formatted ...
Formatted ...
Formatted: Font: Not Bold, Not Highlight
Formatted ...
Formatted ...
Formatted ...
Formatted: Normal
Formatted: Heading 2, Left
Formatted: No underline
Formatted: Font: (Default) Times New Roman
Formatted: Font: (Default) Times New Roman

Table 3. ANP BOCR Result of Priority Aspect

Aspect	Code	Benefit	Opportunity	Cost	Risk	Realistic	Rank
Human Resources at Islamic Social Finance Institution	A	0.1029	0.19763	0.16966	0.12334	0.97181	1
Regulation	B	0.15479	0.09765	0.11073	0.1571	0.8689	2
Supporting Technology Use	C	0.24925	0.16373	0.24824	0.214	0.7682	3
Sustainable Financing	D	0.1229	0.06846	0.10973	0.10029	0.7645	4
Governance	E	0.23496	0.33413	0.17927	0.25364	0.7152	5
Institution	F	0.13521	0.13841	0.18238	0.15163	0.6767	6

Source: Primary Data Prosessed (2021)

The data processing results show that human resource is the highest priority aspect in ISF Islamic social finance institutions, with a value of 0.97181. HThe human resource refers tois all people/staff/employees who participate in the management of ISF [20]. Human resources play a crucial role in Islamic Social Finance management. With a value of 0.8689, the regulation comes in as the second priority. Regulations are all rules put in place to provide legal guidance and support for the management and development of ISF Islamic sSocial fFinance[67]. With a value of 0.7682, the third priority aspect is the use of technology, while sustainable financing comes in the following priority order with a value of 0.7645. Sustainable funding is associated with business development and achieving financial independence using Islamic social funds [68]. Governance is thea fifth priority aspect with a value of 0.7152. The collection, distribution, and utilization process in ISF Islamic social finance are governed by transparency, accountability, responsibility, independence, and fairness [69]. The institutional aspect comes as the least priority, with a score of 0.6767. The institutional aspect relates to the structure and coordination system. In Indonesia, ISF institutions can take the form of government and private legal entities [70]. For zakat management, it can be in the legal entity can take in the form of of the Amil Zakat Institution (LAZ) and the National Amil Zakat Agency (BAZNAS), while. For the management of waqf in the form of a Nazir is the legal entity for the management of waqf..

4.2 Priority solution in integrating sustainable Iislamic social finance

There are eight solutions to integrate sustainable ISF Islamic social finance based on the findings of the focus group, in-depth interviews, and literature review. The first (SL.1) is a (SL.1) financial integration in providing funding to beneficiaries through the distribution of zakat, waqf, and Islamic microfinance based on the beneficiaries' economic circumstances and capabilities. The second step (SL.2) is (SL.2) integrating ISF Islamic social finance with state fiscal instruments, which is supported by existing regulations. Integrating Islamic social philanthropy finance with the fiscal policy can be accomplished by making zakat a tax credit or tax deduction. The the third (SL.3) is (SL.3) an integration through non-formal coordination

Formatted: Left

Formatted: Not Highlight

Formatted: Not Highlight

Commented [A2]: sitasi, cek UU, ZCP, WCP, dll

Formatted: Font: (Default) +Headings CS (Times New Roman)

Formatted: Hyperlink, Font: (Default) +Body (Calibri), 11 pt, Indonesian, Not Highlight

Formatted: Hyperlink, Font: (Default) +Body (Calibri), 11 pt, Indonesian, Not Highlight

Formatted: Not Highlight

Formatted: Not Highlight

Formatted: Not Highlight

Formatted: Not Highlight

Formatted: Not Highlight

Formatted: Not Highlight

Formatted: Font: +Headings CS (Times New Roman)

Formatted: Heading 2, Left

Formatted: Font: (Default) Times New Roman

Formatted: Font: (Default) Times New Roman

between ISF ~~Islamic social finance~~ institutions. The Islamic social finance institutions may establish joint coordinations to conduct empowerment programs, while prioritizing the work specialization of each institution. The f~~F~~ourth solution (SL.4) is ~~(SL.4)~~ an integration among Islamic social finance institutions based on capacity building and role division. This type of integration ~~is dependent on the specialization of each institution and community,~~ includ~~es~~ing vertical and horizontal integrations [70]. ~~This type of integration is dependent on the specialization of each institution and community.~~ The v~~V~~ertical integration is achieved through a collaboration between ISF institutions and the government. The government ~~can~~ collaborates by providing a database of people who are eligible to receive Islamic social finance funds. The government also provides rules and guidance for the management of ISF. –Meanwhile, a horizontal integration ~~is taking~~is conducted ~~place~~ between ISF management institutions, where ~~Islamic social finance~~different -institutions collaborate to carry out joint social programs [32]. ~~Furthermore~~In addition, a horizontal integration is also carried out with academics and associations for future management development of the ISF [20]. –Some organizations concentrate on the health sector, while others concentrate on economic empowerment and education. As a result, combining institutions based on their specialization may be the best option for achieving the research objective. The f~~F~~ifth– (SL.5) is ~~(SL.5)~~ establishing an integrated national data center for all ISF ~~Islamic social finance~~ instruments; the data that must be integrated include financial statements, donor funds, beneficiary data, and other relevant information. The s~~S~~ixth ~~is~~ (SL.6) includes an integration based on regional clusters with a solid authority. This integration may concentrate on integrating Islamic social finance institutions into geographical groups. The seventh solution (SL.7) incorporates integrating ISF ~~Islamic social finance~~ through the passage of new laws and regulations. Each instrument of ISF~~Islamic social finance~~, currently, has its own set of rules and regulations. The eighth solution ~~is~~ (SL.8) is an integration based on ISF ~~Islamic social finance~~ management clusters, such as zakat *Amils* joining other zakat institutions and waqf manager joining other waqf institutions. Table 4 shows the ANP result of solution and the order of priority solutions in the short term, long term, and based on experts' agreement

Table 4. ANP Results of Solution

Cluster	Code	Benefit	Opportunity	Cost	Risk	Net Value	Net Value Priority	Realistic	Reaslistic Priority	Expert Judment	Expert Judment Priority
Solution	SL.1	0.0526	0.0588	0.0466	0.04593	**0.0188**	**1**	**1.4435**	**1**	**0.2118**	**2**
	SL.2	0.0277	0.0262	0.0174	0.02957	**0.0070**	**2**	**1.4149**	**2**	0.1016	4
	SL.3	0.0258	0.0235	0.0227	0.02338	0.0032	5	**1.1452**	**3**	0.0952	5
	SL.4	0.0297	0.0204	0.0226	0.023715	0.0038	4	1.1313	4	0.0929	6
	SL.5	0.0555	0.0657	0.0537	0.063225	**0.0042**	**3**	1.0737	5	**0.2438**	**1**
	SL.6	0.0244	0.0144	0.0260	0.020225	-0.0073	7	0.6719	6	0.0787	7
	SL.7	0.0159	0.0289	0.0380	0.020995	-0.0141	7	0.5777	7	**0.1071**	**3**
	SL.8	0.0180	0.0118	0.0226	0.022965	-0.0157	8	0.4113	8	0.0685	8

Commented [A3]: sitasi, cek UU

Formatted: Font: (Default) +Headings CS (Times New Roman)

Formatted: Not Highlight

Formatted: Not Highlight

Formatted: Not Highlight

Formatted: Not Highlight

Formatted: Not Highlight

Formatted: Not Highlight

Formatted: Not Highlight

Formatted: Not Highlight

Formatted: Not Highlight

Formatted: Not Highlight

Formatted: Not Highlight

Formatted: Not Highlight

Formatted: Not Highlight

Formatted: Not Highlight

Formatted: Not Highlight

Formatted: Left

Formatted Table

Formatted: Font: (Default) Times New Roman

Formatted: Font: (Default) Times New Roman

Source: Primary Data Processed (2021)

The formulated solution wa~~i~~s then processed using the ANP BOCR to determine the priority order of solutions. Short term solution considers the marginal cost of the benefits obtained. ~~While~~O~~o~~n the contrary, ~~At the same time,~~ in the long term, the ~~analysis is~~ ~~a~~ priority is achieved when the total number of benefits and opportunities are ~~can be~~ obtained after ~~by~~ reducing the total cost and risk [63,65]. On the other hand, an expert judgment does not consider the benefits, opportunity, cost, and risk aspects of each solution formulated in this study. ~~Table 4 shows the order of priority solutions in the short term, long term, and based on experts' agreement~~ , while Table 5 summarizes the results of data processing. ~~as follows:~~

Table 5. Summary of Priority Solutions

Solution/Rank	Solution in Sustainable Islamic Social Finance Integration		
	**1**	**2**	**3**
Expert Judgment	Integration of national data centers (SL.5)	Financial integration in providing funding to beneficiaries (SL.1)	Integration through the establishmen of new laws and regulations (SL.7)
Short Term Realistic Value	Financial integration in providing funding to beneficiaries (SL.1)	Integration with state fiscal policy (SL.2)	Integration through non-formal coordination between institutions (SL.3)
Long Term Net Value	Financial integration in providing funding to beneficiaries (SL.1)	Integration with state fiscal policy (SL.2)	Integration of national data centers (SL.5)

Source: Author's compilation (2021)

According to the data processing results, the first and second priorities in the short and long-term are in the same order. The solutions that will be implemented first and foremost in the short and long term are financial integration in providing funding to beneficiaries and integration of Islamic social finance with the state fiscal policy. According to expert judgment, integrating national data centers is the highest priority solution, with financial integration in providing funding to beneficiaries coming in second.

~~4.3~~ Priority strategies in integrating sustainable I~~i~~slamic social finance

There are nine strategies to integrate sustainable ISF ~~Islamic social finance~~ based on the findings of the FGD, in-depth interviews, and literature review. The first strategy (ST.1) is to improve the quality of human resources through training and certification. The second strategy (ST.2) promotes the availability of real-time data through database integration and reporting. The third strategy (ST.3) is optimizing the digital technology in Islamic social finance instruments' collection, management, and distribution process. The fourth ~~is~~ (ST.4) is a coordination and synergy among stakeholders (e.g., practitioners, regulators, associations, and academicians~~, etc.~~) to establish shared commitment and vision. The f~~F~~ifth~~,~~ (ST.5) is encouraging the~~es~~ broadening of the range of benefits by establishing service units at lower levels, such as service units at the district, village, and mosque levels, as well as strategic locations that support the broadening of the reach of the benefits of ISF ~~Islamic social finance~~ instruments. The s~~S~~ixth~~,~~ (ST.6) is establishing a new institution that is capable of integrating

Formatted: Left
Formatted: Font: (Default) +Headings CS (Times New Roman), 12 pt
Formatted: Normal
Formatted: Font: (Default) +Headings CS (Times New Roman), 12 pt
Formatted: Font: (Default) +Headings CS (Times New Roman), 12 pt
Formatted: Not Highlight
Formatted: Not Highlight
Formatted: Not Highlight
Formatted: Not Highlight
Formatted: Not Highlight
Formatted: Not Highlight
Formatted: Not Highlight
Formatted: Not Highlight
Formatted: Not Highlight
Formatted: Left
Formatted: Font: +Headings CS (Times New Roman)
Formatted: Heading 2, Left
Formatted: Font: (Default) Times New Roman
Formatted: Font: (Default) Times New Roman

all instruments of ISF~~Islamic social finance~~. In Indonesia, various institutions manage different ISF ~~Islamic social finance~~ instruments. The seventh strategy ~~is~~ (ST.7) is realizing the government's role in supporting the integration of ISF ~~Islamic social finance~~ through regulation and socialization; several previous studies have concluded that government support for integrating ISF ~~Islamic social finance~~ is still inadequate. The eighth strategy (ST.8) focuses on developing the ISF ~~Islamic social finance~~ institutions to have two functions, namely, *Amil* and *Nazir*. Several Baitul Maals wat Tamwiils (BMT) have~~s~~ operated the two functions under the same body. The n~~N~~inth ~~is~~ strategy (ST.9) is revising and updating all laws and regulations governing the management of Islamic social finance instruments to accommodate the development of ISF ~~Islamic finance~~ instruments. This development includes the management of zakat on profession, corporate zakat, temporary waqf, ~~cryptocurrency waqf,~~ stock waqf, etc. The ANP results and order of priority strategies is shown in Table 6, which displays the short-term, long-term priority solutions, and expert judgment.

Table 6. ANP Results of Strategies

Cluster	Code	Benefit	Opportunity	Cost	Risk	Net Value	Net Value Priority	Realistic	Reaslistic Priority	Expert Judgment	Expert Judgme[illegible] Priority
Strategies	ST.1	0.0455	0.0393	0.0377	0.030375	**0.0168**	**1**	**1.5651**	**1**	**0.1546**	**3**
	ST.2	0.0334	0.0437	0.0297	0.03745	**0.0099**	**2**	**1.3113**	**2**	0.1515	4
	ST.3	0.0500	0.0463	0.0444	0.048875	**0.0030**	**3**	**1.0676**	**3**	**0.1885**	**1**
	ST.4	0.0364	0.0450	0.0443	0.03603	0.0011	4	1.0289	4	**0.1666**	**2**
	ST.5	0.0235	0.0148	0.0181	0.02406	-0.0038	6	0.8001	5	0.0752	6
	ST.6	0.0086	0.0087	0.0089	0.01088	-0.0023	5	0.7807	6	0.0365	9
	ST.7	0.0173	0.0179	0.0213	0.019505	-0.0055	7	0.7485	7	0.0750	7
	ST.8	0.0214	0.0140	0.0203	0.02178	-0.0066	8	0.6796	8	0.0723	8
	ST.9	0.0135	0.0199	0.0250	0.02105	-0.0126	9	0.5124	9	0.0795	5

Source: Data Primary Result (2021)

After ~~the solution has been~~ developing the solution~~ed~~, a strategy must be developed. Strategies are employed to aid in the achievement of solutions, and thus, the integration of ISF will be realized. ~~The order of priority strategies is shown in Ttable 6, which displays the short-term, long-term priority solutions, and expert judgment.~~ The strategies are summarized in T~~t~~able 7. ~~as follows:~~

Table 7. Summary of Priority Strategies

**Strategies**	**Strategies in Sustainable Islamic Social Finance Integration**		
	**1**	**2**	**3**
Expert Judgment	Optimizing the use of digital technology (ST.3)	Coordination and synergy between stakeholders (ST.4)	Improving the quality of h~~H~~uman r~~R~~esources (ST.1)
Short Term Realistic Value	Improving the quality of h~~H~~uman r~~R~~esources (ST.1)	Establishing real time information through database integration and reporting (ST.2)	Optimizing the use of digital technology (ST.3)

Formatted Table

Formatted: Left

Formatted: Not Highlight

Formatted: Not Highlight

Formatted: Left

Formatted Table

Formatted: Font: (Default) Times New Roman

Formatted: Font: (Default) Times New Roman

Strategies	Strategies in Sustainable Islamic Social Finance Integration		
	1	2	3
Long Term Net Value	Improving the quality of h~~H~~uman r~~R~~esources (ST.1)	Establishing real time information through database integration and reporting (ST.2)	Optimizing the use of digital technology (ST.3)

Source: Author's compilation (2021)

The results show that the short and long-term strategies are in the same order. Improving the quality of human resources, establishing real-time database integration and reporting, and optimizing the use of digital technology, become the priority strategies. However, the expert judgment ~~put~~ considers the optimization use of digital technology to be the priority strategy.

~~5.~~ Discussion

~~5.1~~ Priority solution i~~I~~n developing a~~A~~ sustainable I~~i~~slamic social finance integration model

Financial integration in providing financing to beneficiaries is a high-priority solution that can be implemented in the short and long term. ~~-~~Further, this solution is very consistent with the sustainability paradigm, where the funding provided will broaden the range of benefits, and ~~,~~ ultimately~~,~~ creates financial independence for the beneficiaries, as stated by Haneef et al. [45], Azganin et al. [71], Thaker et al. [72], and Widiastuti et al. [20] ~~Haneef et al. (2015), Nurlita and& Ekawaty (2018), Taufiq et al. (2018), Azganin et al. (2021), Thaker et al. (2021), and Widiastuti et al. (2021)~~. Sari et al. [73] shows that by distributing zakat, the time taken by the poor to exit poverty is faster (3.3 years) than without zakat (6.6 years). In addition, a study conducted by Widiastuti et al. [3] ~~which~~ analyze~~d~~s financing programs carried out by the ~~Lembaga Islamic social finance seperti~~ISF institutions, including ~~–~~Yatim Mandiri, Dompet Dhuafa, Inisiatif Zakat Indonesia, Nurul Hayat, YDSF, and Dompet Amanah Umat, and found that they have a significant impact on increasing the ~~on~~ welfare of the poor. The distribution aspect, particularly productive distribution, holds the key to success in ISF integration in transforming the poor into the ~~rich~~capables [69]. A f~~F~~inancial integration through ISF instruments, such as zakat and waqf, will increase production factors. In this case, financing will be more effective due to an increase in the amount of funds obtained by the poor to achieve welfare. This is consistent with the economic integration theory ~~advanced~~discussed ~~-~~by Balassa [57]. This also supports Sanyinna and Omar [74] ~~Hamed's (2020)~~ claim that Islamic~~-~~ financing benefits small businesses by maximizing Islamic social financial instruments (zakat, *infaq*, *sadaqah*, and waqf) as the best strategy for improving the recipients' quality of life. The financing provided through integrating Islamic social finance instruments has a cheap cost of the fund, and~~;~~ thus, it ensures the beneficiaries' welfare. Furthermore, the integration of financing demonstrates that Islamic social fund instruments serve multiple purposes: C~~c~~onsumptive zakat is used to provide an emergency assistance in the short term, while productive zakat and benevolence funds are used to support economic recovery in the medium term, and waqf or Sukuk are used to build long-term resilience. The financial integration of Islamic social ~~fund~~ finance instruments has also occurred in several institutions, although only on a micro-scale. Several institutions in Indonesia have worked to integrate zakat and waqf

Commented [A4]: Cek lagi

funds. Dompet Dhuafa, for example, integrated zakat and waqf funds when constructing an Integrated Health Home (Hospital). Cash Waqfs are used to build infrastructure on waqf land, whereas zakat funds provide free health care to the poor. Further, recently, Dompet Dhuafa, BAZNAS, and BWI have collaborated to integrate zakat and waqf, the newly established MoU, to collaborate on social projects.

This priority, however, differs from expert opinion, which emphasizes the importance of integrating nationally centralized data, such as database on donors, beneficiaries, and financial reports~~, and so on~~. According to Afriadi and Sanrego [54] and Hudaefi et al. [75] ~~[50,68,69],~~ the formation of national-centered data is critical for optimizing the integration and management of ISF ~~Islamic social finance~~. The integration of beneficiary-related information is crucial for detecting data on beneficiary development and preventing overlapping distributions. Since ISF ~~Islamic social funds~~ are still managed in a fragmented manner (partially), no single platform currently integrates the data from all instruments of ISF ~~Islamic social finance~~ in Indonesia. As the latest development, a data integration platform~~,~~ has been established in the zakat sector. BAZNAS, an Indonesian zakat institution and regulator, released SIMBA (BAZNAS Information Management System), an integrated platform for zakat database management from all BAZNAS and LAZNAS across Indonesia. This platform only integrates the data from national zakat collections. As a result, establishing a central platform capable of integrating data on all Islamic social financial~~e~~ instruments is critical.

Financial integration and integration of national data centers are interrelated. ~~-~~This is so since funding distribution necessitates specific data to ensure the long-term viability of the funds provided. Data integration is needed to reduce the potential risk and realize the collection of distributed financings so that the supply of funds will be guaranteed. Further, the integration of beneficiary data is needed to ensure the depth of the reach of benefits and the creation of financial independence, and~~;~~ thus, sustainability will be achieved. On another side, financial integration remains a short- and long-term priority because it is directly related to the primary goal of Islamic social finance, which is to create prosperity by improving the business and economic capacity of beneficiaries. As one of the priority solutions in this research, the regulator must provide a legal environment to accommodate all changes and developments in the management of Islamic s~~S~~ocial f~~F~~inance. First, the government must revise the law to accommodate the rapid growth of Islamic social finance, instruments [76]~~–~~. Second, the government must socialize the revised laws and regulations to all stakeholders, and make the law more socially acceptable, [20,77]. Third, encourage ISF management institutions to implement the law by offering rewards or incentives that the Institution can use, such as free training and~~,~~ accompaniment [20]. ~~and s~~

~~5.2~~ Strategic priorities i~~I~~n developing a~~A~~ sustainable I~~i~~slamic social finance integration model

According to expert judgments, the strategy with the highest priority is optimizing the use of digital technology. This ~~goes~~ in line with Thaker et al. [78], Usman et al. [79], Berakon et al. [80], Nor et al. [81], and Widiastuti et al. [20]. ~~Saad et al. (2017), Thaker et al. (2018), Muneeza~~

Formatted: Not Highlight
Formatted: Hyperlink, Font: (Default) +Body (Calibri), 11 pt, Not Highlight
Formatted: Not Highlight
Formatted: Hyperlink, Font: (Default) +Body (Calibri), 11 pt, Not Highlight
Formatted: Not Highlight
Field Code Changed
Formatted: Not Highlight
Commented [A5]: sitasi
Formatted: Not Highlight
Formatted: Not Highlight
Formatted: Font: (Default) +Headings CS (Times New Roman)
Formatted: Not Highlight
Formatted: Hyperlink, Font: (Default) +Body (Calibri), 11 pt, Not Highlight
Formatted: Not Highlight
Formatted: Not Highlight
Formatted: Not Highlight
Formatted: Not Highlight
Formatted: Not Highlight
Formatted: Not Highlight
Formatted: Not Highlight
Formatted: Not Highlight
Formatted ...
Formatted: Not Highlight
Formatted: Font: 16 pt, Font color: Text 1
Formatted: Heading 2, Left
Formatted: Font: +Headings CS (Times New Roman)
Formatted: Not Highlight
Formatted ...
Formatted: Not Highlight
Formatted ...
Formatted: Not Highlight
Formatted ...
Formatted: Not Highlight
Formatted ...
Formatted: Not Highlight
Formatted ...
Formatted: Not Highlight
Field Code Changed
Formatted ...
Formatted ...
Formatted ...
Formatted: Font: (Default) Times New Roman
Formatted: Font: (Default) Times New Roman

and& Nadwi (2019), Usman et al. (2020), Berakon et al. (2021), Nor et al. (2021), and Widiastuti et al. (2021), all of which emphasize the importance of using technology in managing the instrument of ISFIslamic social finance. First, Technologies that can be employed include technology for creating a centralized database linked to the government and Islamic social finance supervisors (Widiastuti , Cahyono, et al., 2021). This strategy is also linked to the immediate solution, merging national data centers for all Islamic social and financial instrumentsThis strategy is also connected to the immediate solution, integrating national data centers for all Islamic social and finance instruments. Data integration requires the development of an advanced web-based platform that all stakeholders can use in order to become effective and efficient.To become effective and efficient, data integration necessitates the development of an advanced web-based platform that all stakeholders can use, which necessitates the optimization of digital technology. Second, technology in supervision and monitoring (Rachman & Nur Salam, 2018). Third, technology in the marketing component to assist the target collection of Islamic Social Finance instruments (Hadi et al., 2021) (Rahmatina Awaliah Kasri & Yuniar, 2021). Fourth, technology enables management reports to be generated in real time and made available to the public (Widiastuti, Cahyono, et al., 2021).This strategy is also connected to the immediate solution, integrating national data centers for all Islamic social and finance instruments. To become effective and efficient, data integration necessitates the development of an advanced web-based platform that all stakeholders can use, which necessitates the optimization of digital technology.tTechnology for creating a centralized database linked to the government and ISF Islamic social finance supervisors is one example of a technology that can be used [20]. d (Widiastuti et al., 2021). This strategy is also linked to the immediate solution, which is the consolidation of national data centers for all Islamic social and financial instruments. To become effective and efficient, data integration necessitates the creation of a sophisticated web-based platform that all stakeholders can access. Second, technological advancements in supervision and monitoring [20]. (Rachman & Salam, 2018). Third, technology in the marketing component to aid in the collection of target achievement [82,83]. (Hadi et al., 2021; Kasri & Yuniar, 2021). Fourth, technology to generate real-time management reports and make them available to the publipublic [20]c. (Widiastuti et al., 2021).

However, in terms of short-term and long-term priorities, optimizing the use of technology has slipped to third place. Experts consider improving the quality of hHuman rResources to be the most important aspect, both in the short and long -term. The importance of enhancing the capability of human resources goes in line with Karami et al. [84], Hidayatullah and Priantina [21], Ali et al. [55], and Widiastuti et al. [20]. Hidayatullah and& Priantina (2018), Ali et al. (2020), and Widiastuti et al. (2021). The difference in priorities between expert judgments and short/long term orientations occurs because of technology investment, which is considered a long-term investment, necessitates a significant funding source, which some zakat institutions may not afford [20]. (Widiastuti, Cahyono, et al., 2021). Optimizing the use of technology, which requires a large amount of investment, is a challenge for Islamic social finance institutions, particularly in Indonesia. This is due to the fact that the operational costs incurred by ISF Islamic social fund institutions are determined by the amount of funds that can be raised. Meanwhile, there is a significant gap between the realization and potential of ISFIslamic social funds. As a result, institutions prefer to improve competencies and capabilities of human

Commented [A6]: sitasi

resources, relevant to their respective fields of work compared to improving technological features. This is also consistent with the aspects of BOCR addressed in this study. The cost of optimizing technology use is 0.0537, higher than the cost of improving the quality of human resources, which is around 0.0466. Costs associated with technology investments include acquiring digital channels, website management, social media, purchase of computers, and maintenance cost~~, etc~~.

Furthermore, when determining priority strategies in the short and long term, experts also consider the risks exposed. The risk of optimizing the use of technology is high (0.0632), with the benefits obtained are also higher (0.0555). Meanwhile, the threat from the institution's strategy to improve the quality of human resources is lower at 0.0459. Cybercrime is one of the risks of optimizing the use of technology in ISF ~~Islamic social finance~~[85] ~~(72)~~. To mitigate this risk, competent human resources in their field are required. Implementing technology without qualified human resources will result in sub-optimal management. This is consistent with the findings of Widiastuti et al. [20] ~~Widiastuti et al. (2021)~~ that~~,~~ ~~who found~~ a limited number of human resources is capable of optimizing the use of advanced technology, particularly in those newly established institutions. Therefore, experts place qualified human resources as the prioritized strategy to mitigate risks, not only risks associated with technology application, but all risks associated with the integration of sustainable Islamic social finance.

Optimizing the use of technology and improving human resource quality can be done concurrently as stated by Widiastuti et al. [20]~~Widiastuti et al. (2021)~~ that highlights~~ed~~ the importance of improving the quality of zakat management resources, particularly in the technological aspect, by emphasizing technology intensification (by developing *Amil*~~'~~*s'* ability to use technology) and extensification (by increasing the number of *Amils* who master technology). Improving the quality of human resources is the foundation for the long-term integration of ISF~~Islamic social finance~~, while technology facilitates an optimal ISF ~~Islamic social finance~~ management.

Finally, in order to integrate sustainable ISF~~Islamic social finance~~, stakeholders must prioritize solutions and strategies, by ~~taking into account~~considering the benefits, opportunities, costs, and risks (BOCR) of each priority solution and strategy. Improving the quality of human resources and optimizing the use of digital technology are two strategies that must be implemented immediately. Based on the short~~-~~ and long-term analysis and expert consensus, the two strategies are given the highest priority.

~~6.~~ Managerial r~~R~~elevancy

This study has several implications for stakeholders. The findings of this study emphasize solutions and priority strategies for integrating ISF~~Islamic social finance~~, with Islamic social finance institutions playing a critical role in providing certification and training to improve the quality of human resources. Each training activity and accreditation must be given in accordance with the employee's field of work. Technology optimization, such as big data management, data security, e-marketing, and others, are examples of certification and training that must be provided. Human resource quality must be directed ~~at~~to obtain management and technical skills. Managers are encouraged to have managerial skills, such as making strategic decisions, forecasting, and imposing rewards and punishments. Staff are encouraged to learn

Formatted: Font: (Default) +Headings CS (Times New Roman)

Formatted: Not Highlight

Formatted: Hyperlink, Font: (Default) +Body (Calibri), 11 pt, Not Highlight

Formatted: Not Highlight

Formatted: Font: (Default) +Headings CS (Times New Roman)

Formatted: Not Highlight

Formatted: Hyperlink, Font: (Default) +Body (Calibri), 11 pt, Not Highlight

Formatted: Font: (Default) +Headings CS (Times New Roman)

Formatted: Font: Italic

Formatted: Font: Italic

Formatted: Font: +Headings CS (Times New Roman)

Formatted: Heading 1, Left, Space After: 0 pt, Line spacing: single

Formatted: Font: +Headings CS (Times New Roman)

Formatted: Indent: First line: 0"

Formatted: Not Highlight

Formatted: Not Highlight

Formatted: Font: (Default) Times New Roman

Formatted: Font: (Default) Times New Roman

technical skills, such as collection program operation, *mustahik* guidance, reporting, and administration~~, and so on~~.

The government, as a regulator, must assist Islamic ~~philanthropic~~ social finance institutions in providing facilities or infrastructure to improve the quality of human resource managers, such as free training and certification for managers. Further, no law or regulation has required ISF ~~Islamic sSocial fFinance~~ managers to have standardized or specific competencies. As a result, one form of input for the revision of the law and regulation requires all ISF ~~Islamic sSocial fFinance~~ managers to have specific competencies in managing Islamic ~~philanthropic~~ social finance institutions so that these institutions can be managed more professionally.

This study also emphasizes the significance of financial integration and data integration on the national scale. Associations involved in Islamic ~~philanthropy~~ social finance can use the research findings to establish strategic planning on integrating ISF~~Islamic sSocial fFinance~~ instruments. From an academic standpoint, the results of this study develop the previous studies on the integration of the ISF ~~Islamic sSocial fFinance~~ model, which were conducted without using specific mathematical methods. This study used~~s~~ a systematic approach to create an ideal integration model that can be of priorities both in the short and long term, while also taking the aspect of BOCR into account.

~~7.~~ Conclusion

This research used the~~s~~ ANP BOCR to develop priority solutions and strategies to integrate Islamic social finance based on expert agreement, short-term and long-term analysis. The aspect of human resources, regulation, and technology~~ical~~ use become the highest aspect to be considered in achieving the research objective. Priority solutions in the short and long term~~s~~ have the same order of priorities. Financial integration in providing funding to beneficiaries and integration with the state fiscal policy is the first and second priority solution. Meanwhile, integration through a non-formal coordination between institutions is the third priority solution in the long term. Besides, integrating national data centers has been the third priority solution for a long ~~time~~ term and the highest solution based on expert judgments. In essence, the resulting priority solutions relate to priority aspects ~~of~~ include financing, regulation, and technology.

As a strategy, improving the quality of h~~H~~uman r~~R~~esources, establishing real-time information through database integration and reporting, and optimizing digital technology are three priority strategies that can be implemented in the short and long term. Furthermore, the experts mentioned the need for coordination and synergy between stakeholders in integrating sustainable Islamic social finance. To successfully incorporate Islamic social finance and ensure sustainability, stakeholders are encouraged to implement the research results (solutions and strategies~~y~~).

~~8.~~ Limitation and ~~Study~~ f~~F~~uture r~~R~~esearch~~Forward~~

This research only focuses on six aspects to develop ideal solutions and strategies, however, this research has thoroughly examined each aspect's benefits, opportunities, costs, and risks. Future research can consider essential elements that have not been addressed in developing a sustainable Islamic social finance integration model, such as cultural aspects, donor behavior, and government support.

Formatted: Not Highlight
Formatted: Font: Italic
Formatted: Not Highlight
Formatted: Indent: First line: 0"
Formatted: Font: +Headings CS (Times New Roman)
Formatted: Heading 1, Left, Space After: 0 pt, Line spacing: single
Formatted: Font: +Headings CS (Times New Roman)
Formatted: Heading 1, Left, Space After: 0 pt, Line spacing: single
Formatted: Font: +Headings CS (Times New Roman)
Formatted: Not Highlight
Formatted: Font: (Default) Times New Roman
Formatted: Font: (Default) Times New Roman

This research only focuses on six aspects to develop ideal solutions and strategies: Ggovernance, sustainable financing, institutions, regulations, institutional human resources, and technology. However, this research has thoroughly examined each aspect's benefits, opportunities, costs, and risks. Future research can consider essential elements that have not been addressed in developing a sustainable Islamic social finance integration model, such as cultural aspects, donor behavior, and government support, and others.

Acknowledgment

This research grant is a Covid-19 Research Mandate with the contract number 1025/UN3.15/PT/2021. The authors would like to thank the Research and Community Service Institution (LPPM) of Universitas Airlangga for their financial assistance. The authors would like to thank Center Research and Publication (3P) of Faculty Economic and Business Universitas Airlangga as ethics committee. –The authors would also like to thank all academicians, regulators, associations, practitioners, and community leaders for their support and all of the research's team for their support in writing this research. The authors also appreciate the research's team members for their support in the completion of this research.

References

1. Ibrahim P, Ghazali R. Zakah as an Iislamic micro-financing mechanism to productive zakah recipients. Asian Econ Financ Rev 4(1), 117-125. 2014;4(1):117–25.
2. Zauro NA, Saad RAJ, Ahmi A, Mohd Hussin MY. Integration of waqf towards enhancing financial inclusion and socio-economic justice in Nnigeria. Int J Ethics Syst. 2020;36(4):491–505. doi:doi: 10.1108/IJOES-04-2020-0054
3. Widiastuti T, Auwalin I, Rani LN, Mustofa MU Al. A mediating effect of business growth on zakat empowerment program and mustahiq's welfare. Cogent Bus Manag. 2021;8(1):1882039. doi:doi: 10.1080/23311975.2021.1882039
4. Abdullah M. Waqf, Sustainable Development Goals (sdgsSDGs) and maqasid al-shariah. Int J Soc Econ. 2018;45(1):158–72. doi:doi: 10.1108/IJSE-10-2016-0295
5. Ascarya A. The role of islamic Islamic social finance during covidCOVID-19 pandemic in Iindonesia's economic recovery. Int J Islam Middle East Financ Manag. 2021; doi:doi: 10.1108/IMEFM-07-2020-0351
6. Robani A, Salih K. Positioning Iislamic gift economy for sustainable development at the local level. Humanit Soc Sci Rev. 2018;6(2):111–20. doi:doi: 10.18510/hssr.2018.6214
7. Abdullahi A, Othman AHA, Kassim S. Financial inclusion enhancement through the adoption of Iislamic microfinance in nigeria. Int J Ethics Syst. 2021;37(3):486–505. doi:doi: https://doi.org/10.1108/IJOES-02-2021-0040
8. Raies A. Islamic versus conventional fiscal policy: Tthe effect of zakat on education and employment. Acad J Interdiscip Stud. 2020;9(1):27–33. doi:doi: 10.36941/ajis-2020-0003
9. Rusydiana AS, Sukmana R, Laila N. Waqf on education: a bibliometric review based on Sscopus. Libr Philos Pract. 2021;2021(June):5537.
10. Suprayitno E, Aslam M, Harun A. Zakat and sdgsSDGs: Iimpact zakat on human

Formatted: Indent: First line: 0"

Formatted: Font: +Headings CS (Times New Roman)

Formatted: Heading 1, Left, Space After: 0 pt, Line spacing: single

Formatted: Not Highlight

Formatted: Not Highlight

Formatted: Font: +Headings CS (Times New Roman), Not Bold, English (United States)

Formatted: Justified

Formatted: Font: (Default) Times New Roman

Formatted: Font: (Default) Times New Roman

development in the five states of M~~m~~alaysia. Int J Zakat. 2017;2(1):61–9.

11. Riyanto S, Herningtyas R, Nizar M. Muslim's philanthropy in S~~s~~ingapore and the role of government. Sociolohia Y Tecnociencia. 2021;11(2):194–214. ~~doi:~~doi: https://doi.org/ 10.24197/st.2.2021.194-214

12. IFSB. Islamic financial services industry stability report. Malaysia; 2021.

13. Gärde J. Concepts on zakat, carita~~–~~, and diaconia in the changing social welfare landscape of europe. J Relig Spiritual Soc Work Soc Thought. 2017;36(1–2):164–98. ~~doi:~~doi: 10.1080/15426432.2017.1311242

14. Gatto M, Islam AHMS. Impacts of ~~covid~~COVID-19 on rural livelihoods in B~~b~~angladesh : evidence using panel data. PLoS One. 2021;16(11):e0259264. ~~doi:~~doi: 10.1371/journal.pone.0259264

15. Suryahadi A, Izzati R Al, Suryadarma D. Estimating the impact of ~~covid~~COVID-19 on poverty in I~~i~~ndonesia*. Bull Indones Econ Stud. 2020;0(0):175–92. ~~doi:~~doi: 10.1080/00074918.2020.1779390

16. World Bank. From containment to recovery [Internet]. World Bank. 2020. Available from: https://openknowledge.worldbank.org/bitstream/handle/10986/34497/9781464816413.pdf

17. Ministry of Finance. APBN kita: K~~k~~inerja dan fakta. Kemenkeu.Go.Id. 2020.

18. Ministry of Finance. Survei dan analisis insentif perpajakan program ~~pen~~ PEN 2020 tunjukkan dampak positif. 2021.

19. BAZNAS. Outlook zakat indonesia 2021. Jakarta: Pusat Kajian Strategis - Badan Amil Zakat Nasional (PUSKAS BAZNAS); 2021. 1–144 p.

20. Widiastuti T, Cahyono EF, Zulaikha S, Mawardi I, Al Mustofa MU. Optimizing zakat governance in E~~e~~ast J~~j~~ava using Analytical Network Process (~~anp~~ANP): T~~t~~he role of Zakat Technology (Z~~z~~akaT~~t~~ech). J Islam Account Bus Res. 2021;12(3):301–19. ~~doi:~~doi: 10.1108/JIABR-09-2020-0307

21. Hidayatullah A, Priantina A. Toward zakat management integration in I~~i~~ndonesia: proble~~s~~ms and solution. Ahkam J Ilmu Syariah. 2018;18(2):321–46. ~~doi:~~doi: 10.15408/ajis.v18i2.6319

22. Amalia E. The shariah governance framework for strengthening zakat management in I~~i~~ndonesia: a critical review of zakat regulations. In: Advances in Social Science, Education and Humanities Research. 2018. p. 133–8. ~~doi:~~doi: 10.2991/iclj-17.2018.28

23. Center of Strategic Studies BAZNAS. Indeks literasi zakat: T~~t~~eori dan konsep. Jakarta Pusat: Pusat Kajian Strategis – Badan Amil Zakat Nasional (Puskas); 2019. 1–46 p.

24. Pitchay AA, Thaker MAMT, Mydin AA, Azhar Z, Latiff ARA. Cooperative-waqf model: A~~a~~ proposal to develop idle waqf lands in M~~m~~alaysia. ISRA Int J Islam Financ. 2018;10(2):225–36. ~~doi:~~doi: 10.1108/IJIF-07-2017-0012

25. Ahmad M. An empirical study of the challenges facing zakat and waqf institutions in N~~n~~orthern N~~n~~igeria. ISRA Int J Islam Financ. 2019;11(2):338–56. ~~doi:~~doi: 10.1108/IJIF-04-2018-0044

26. Sukmana R. Critical assessment of islamic endowment funds (waqf) literature: L~~l~~esson for government and future directions. Heliyon. 2020;6(10):e05074. ~~doi:~~doi:

Formatted: Font: (Default) Times New Roman

Formatted: Font: (Default) Times New Roman

10.1016/j.heliyon.2020.e05074

27. Ali KM. Integrating zakah, awqaf and ~~imf~~ IMF for poverty alleviation: T~~t~~hree models of I~~i~~slamıc micro finance. Irti. 2014;2(3):193–211. ~~doi:~~doi: 10.13140/RG.2.2.31888.40969

28. Lubis M, Azizah AH. Towards achieving the efficiency in zakat management system: interaction design for optimization in I~~i~~ndonesia ~~muharman~~. Commun Comput Inf Sci. 2018;886(March 2017):vi. ~~doi:~~doi: 10.1007/978-981-13-1628-9

29. IRTI. Islamic social finance report 2014. Islam and the Moral Economy. 2014. ~~doi:~~doi: 10.1017/cbo9780511617614.005

30. Sudirman. Government policy on zakat and tax in I~~i~~ndonesia. AHKAM J Ilmu Syariah. 2015;15(1):1–14.

31. Raimi L, Patel A, Adelopo I. Corporate social responsibility, waqf system and zakat system as faith-based model for poverty reduction. World J Entrep Manag Sustain Dev. 2014;10(3):228–42. ~~doi:~~doi: 10.1108/wjemsd-09-2013-0052

32. Jouti AT. An integrated approach for building sustainable I~~i~~slamic social finance ecosystems. ISRA Int J Islam Financ. 2019;11(2):246–66. ~~doi:~~doi: 10.1108/IJIF-10-2018-0118

33. Shalleh MCM, Razali SS, Laksana NNM, Embi NAC, Abdullah NI. Developing a sustainable model of waqf-based takaful for flood victims in M~~m~~alaysia. J Islam Account Bus Res. 2020;11(9):1941–52. ~~doi:~~doi: 10.1108/JIABR-10-2016-0114

34. IRTI. Islamic social finance report 2020. 2020.

35. Kuanova LA, Sagiyeva R, Shirazi NS. Islamic social finance: A~~a~~ literature review and future research directions. J Islam Account Bus Res. 2021;12(5):707–28. ~~doi:~~doi: 10.1108/JIABR-11-2020-0356

36. Mateev M, Tariq MU, Sahyouni A. Competition, capital growth and risk-taking in emerging markets: P~~p~~olicy implications for banking sector stability during ~~covid~~COVID-19 pandemic. PLoS One. 2021;16(6):e0253803. ~~doi:~~doi: 10.1371/journal.pone.0253803

37. Arikan G, Bloom PB-N. “I was hungry and you gave me food”: R~~r~~eligiosity and attitudes toward redistribution. PLoS One. 2019;14(3):e0214054. ~~doi:~~doi: ~~https://doi.org/~~10.1371/journal. pone.0214054

38. Adjar H, Chakir A, Meskine L El. Exploratory study of I~~i~~slamic social finance in M~~m~~orocco. Int Rev Entrep Financ. 2020;3(2):45–69.

39. Hudaefi FA. How does I~~i~~slamic fintech promote the ~~sdgs~~SDGs? Q~~q~~ualitative evidence from I~~i~~ndonesia. Qual Res Financ Mark. 2020;12(4):353–66. ~~doi:~~doi: 10.1108/QRFM-05-2019-0058

40. Kailani N, Slama M. Accelerating I~~i~~slamic charities in indonesia: Z~~z~~akat, sedekah and the immediacy of social media. South East Asia Res. 2020;28(1):70–86. ~~doi:~~doi: 10.1080/0967828X.2019.1691939

41. Kasri RA. Management of zakah distribution: E~~e~~mpirical evidence from I~~i~~ndonesia. AFEBI Islam Financ Econ Rev. 2017;

42. Bilo C, Machado AC. The role of zakat in the provision of social protection: A~~a~~ comparison between J~~j~~ordan and S~~s~~udan. Int J Sociol Soc Policy. 2020;40(3–4):236–48.

Formatted: Font: (Default) Times New Roman

Formatted: Font: (Default) Times New Roman

~~doi:~~doi: 10.1108/IJSSP-11-2018-0218

43. Manurung S. Islamic religiosity and development of zakat institution. Qudus Int J Islam Stud. 2013;1(2):197–220. ~~doi:~~doi: 10.21043/QIJIS.V1I2.186

44. Arifin N, Anwar AZ. The improvement model of microenterprises of post-disaster through empowerment of productive zakat. J Gov Regul. 2021;10(4):156–63. ~~doi:~~doi: 10.22495/jgrv10i4art14

45. Haneef MA, Pramanik AH, Mohammed MO, Bin Amin MF, Muhammad AD. Integration of waqf-I~~i~~slamic microfinance model for poverty reduction: T~~t~~he case of bangladesh. Int J Islam Middle East Financ Manag. 2015;8(2):246–70. ~~doi:~~doi: 10.1108/IMEFM-03-2014-0029

46. Pischke JD Von. Measuring the trade-off between outreach and sustainability of microenterprise lenders. J Int Dev. 1996;8(2):225–39. ~~doi:~~doi: 10.1002/(SICI)1099-1328(199603)8:2<225::AID-JID370>3.0.CO;2-6

47. Chow TC, Zailani S, Rahman MK, Qiannan Z, Bhuiyan A, Patwary AK. Impact of sustainable project management on project plan and project success of the manufacturing firm: S~~s~~tructural model assessment. PLoS One. 2021;16(11):e0259819. ~~doi:~~doi: 10.1371/journal.pone.0259819

48. Brau JC, Woller GM, Brau JC, Woller GM. Microfinance: A~~a~~ comprehensive review of the existing literature. J Entrep Financ. 2004;9(1):1–28.

49. Ali AFM, Rashid ZA, Johari F, Aziz MRA. The effectiveness of zakat in reducing poverty incident: A~~a~~n analysis in K~~k~~elantan, M~~m~~alaysia. Asian Soc Sci. 2015;11(21):355–67. ~~doi:~~doi: 10.5539/ass.v11n21p355

50. Ahmed BO, Johari F, Wahab KA. Identifying the poor and the needy among the beneficiaries of zakat need for a zakat-based poverty threshold in N~~n~~igeria. Int J Soc Econ. 2017;44(4):446–58. ~~doi:~~doi: 10.1108/IJSE-09-2015-0234

51. Bin-Nashwan SA, Abdul-Jabbar H, Aziz SA, Viswanathan KK. A socio-economic model of zakah compliance. Int J Sociol Soc Policy. 2020;40(3–4):304–20. ~~doi:~~doi: 10.1108/IJSSP-11-2019-0240

52. Amalia E. Good governance for zakat institutions in I~~i~~ndonesia: a confirmatory factor analysis. Pertanika J Soc Sci Humanit. 2019;27(3):1815–27.

53. Nguyen-Van P, Stenger A, Tiet T. Social incentive factors in interventions promoting sustainable behaviors : a meta- analysis. PLoS One. 2021;16(12):e0260932. ~~doi:~~doi: 10.1371/journal.pone.0260932

54. Afriadi F, Sanrego YD. Mengurai permasalahan distribusi zakat kepada fakir miskin: pengalaman organisasi pengelola zakat ~~baznaz~~BAZNAS, D~~d~~ompet H~~d~~huafa, dan L~~l~~AZIZMU. J Madania. 2016;20(1):23–36. ~~doi:~~doi: 10.29300/madania.v20i1.83

55. Ali MM, Devi A, Furqani H, Hamzah H. Islamic financial inclusion determinants in I~~i~~ndonesia: A~~a~~n ANP approach. Int J Islam Middle East Financ Manag. 2020;13(4):727–47. ~~doi:~~doi: 10.1108/IMEFM-01-2019-0007

56. Ryandono MNH, Qulub AS, Cahyono EF, Widiastuti T, Aisyah BN, Robani A. Efficiency analysis of zakat institutions in I~~i~~ndonesia: D~~d~~ata Envelopment Analysis (~~dea~~DEA) and Free Disposal Hull (~~fdh~~FDH) approaches. Acad Account Financ Stud J. 2021;25(6):1–12.

57. Balassa B. Economic integration. world Econ. 1991;176–86. ~~doi:~~doi:

Formatted: Font: (Default) Times New Roman

Formatted: Font: (Default) Times New Roman

10.47577/technium.v2i4.860

58. Kang M, Yang MG (Mark), Park Y, Huo B. Supply chain integration and its impact on sustainability. Ind Manag Data Syst. 2018; doi:doi: 10.1108/IMDS-01-2018-0004

59. Bong A, Premaratne G. The impact of financial integration on economic growth in Southeast Asia. J Asian Financ Econ Bus. 2019;6(1):107–19. doi:doi: 10.13106/jafeb.2019.vol6.no1.107

60. Hassanain KM. Integrating zakah, awqaf, and imf IMF for poverty allevation: Tthree models of Iislamic micro finance. J Econ Soc Thought. 2015;2(2):106–20.

61. Ambrose AHAA, Hassan MAG, Hanafi H. A proposed model for waqf financing public goods and mixed public goods in Mmalaysia. Int J Islam Middle East Financ Manag. 2018;11(3):395–415. doi:doi: 10.1108/IMEFM-01-2017-0001

62. Saaty TL. Decision making with the analytic network process. In: International Series in Operations Research & Management Science. Springer; 2013. p. 1–40.

63. Saaty TL, Vargas LG. Decision making with the analytic network process: Eeconomic, political, social and technological application with benefit, opportunities, cost and risks. II. Vol. 195, International Series in Operations Research and Management Science. London: Springer; 2006. 1–370 p. doi:doi: 10.1007/978-1-4614-7279-7_15

64. Ascarya. Analytic Network Process (ANPnp): Ppendekatan baru studi kualitatif. Cent Cent Bank Educ Stud Bank Indones. 2005;

65. Zammori F, Bigliardi B, Carmignani G. Selecting inbound logistic policies: Aan anpANP-based multi criteria decision making approach. Int J Logist Syst Manag. 2014;19(2):212–44. doi:doi: 10.1504/IJLSM.2014.064659

66. Firmansyah I, Sukmana W. Analisis problematika zakat pada baznas BAZNAS Kota Tasikmalaya: Ppendekatan metode Analytic Network Process (ANPnp). J Ris Akunt dan Keuang. 2014;2(2):392. doi:doi: 10.17509/jrak.v2i2.6593

67. Munandar A, Syakhroza A, Martani D, Siswantoro D. Does regulation increase zakat payment? Acad Account Financ Stud J. 2019;23(6).

68. Sulaiman M, Zakari MA. Financial sustainability of State Waqf Institutions (SwWIsis) in Mmalaysia. J Islam Account Bus Res. 2019;10(2):236–58. doi:doi: 10.1108/JIABR-05-2016-0054

69. Adiwijaya ZA, Suprianto E. Good governance of zakat institutions: a A literature review. J Southwest Jiaotong Univ. 2020;55(2):1–7. doi:doi: 10.35741/issn.0258-2724.55.2.38

70. Minister of Law and Human Rights Republic of Indonesia. Undang-undang republik indonesia nomor 23 tahun 2011 tentang pengelolaan zakat. 2011.

71. Azganin H, Kassim S, Auwal Adam S. Proposed waqf crowdfunding models for small farmers and the required parameters for their application. Islam Econ Stud. 2021; doi:doi: 10.1108/IES-01-2021-0006

72. Thaker MAMT, Amin MF, Thaker HMT, Khaliq A, Pitchay AA. Cash waqf model for micro enterprises' human capital development. ISRA Int J Islam Financ. 2021;13(1):66–83. doi:doi: 10.1108/ijif-08-2018-0091

73. Sari DF, Beik IS, Rindayati W. Investigating the impact of zakat on poverty alleviation : a A case from Wwest Sumatra, Indonesia. Int J Zakat. 2019;4(2):1–12. doi:doi:

Formatted: Font: (Default) Times New Roman

Formatted: Font: (Default) Times New Roman

https://doi.org/10.37706/ijaz.v4i2.180

74. Sanyinna AY, Omar HH. Employing cash waqf in financing micro, small and medium enterprises for sustainable poverty alleviation-a grounded theory approach. In: Proceedings of International Conference on Zakat, Tax, Waqf And Economic Development (ZAWED). 2019. p. 37–48.

75. Hudaefi FA, Caraka RE, Wahid H. Zakat administration in times of ~~covid~~COVID-19 pandemic in I~~i~~ndonesia: a knowledge discovery via text mining. Int J Islam Middle East Financ Manag. 2021; ~~doi:~~doi: 10.1108/IMEFM-05-2020-0250

76. Mutamimah M, Alifah S, Gunawan G, Adnjani MD. ICT-based collaborative framework for improving the performance of zakat management organisations in I~~i~~ndonesia. J Islam Account Bus Res. 2021; ~~doi:~~doi: 10.1108/jiabr-05-2020-0154

77. Saad RAJ, Farouk AU. A comprehensive review of barriers to a functional zakat system in N~~n~~igeria: what needs to be done? Int J Ethics Syst. 2019;35(1):24–42. ~~doi:~~doi: 10.1108/IJOES-06-2018-0090

78. Thaker MAMT, Thaker HMT, Ptchay AA. Modeling crowdfunders' behavioral intention to adopt the C~~c~~rowdfunding-Waqf Model (~~cwm~~CWM) in M~~m~~alaysia: the theory of the technology acceptance model. Int J Islam Middle East Financ Manag. 2018;11(2):231–49. ~~doi:~~doi: 10.1108/IMEFM-06-2017-0157

79. Usman H, Mulia D, Chairy C, Widowati N. Integrating trust, religiosity and image into technology acceptance model: T~~t~~he case of the I~~i~~slamic philanthropy in I~~i~~ndonesia. J Islam Mark. 2020; ~~doi:~~doi: 10.1108/JIMA-01-2020-0020

80. Berakon I, Aji HM, Hafizi MR. Impact of digital sharia banking systems on cash-waqf among I~~i~~ndonesian muslim youth. J Islam Mark. 2021; ~~doi:~~doi: 10.1108/JIMA-11-2020-0337

81. Nor SM, Abdul-Majid M, Esrati SN. The role of blockchain technology in enhancing islamic social finance: T~~t~~he case of zakah management in M~~m~~alaysia. Foresight. 2021;23(5):509–27. ~~doi:~~doi: 10.1108/FS-06-2020-0058

82. Hadi R, Sochimin, Basit A. Sharia strategic economic model on digital zakat technology in I~~i~~ndonesia. Econ Ann. 2021;187(1/2):82–9. ~~doi:~~doi: 10.21003/EA.V187-08

83. Kasri RA, Yuniar AM. Determinants of digital zakat payments: L~~l~~essons from I~~i~~ndonesian experience. J Islam Account Bus Res. 2021;12(3):362–79. ~~doi:~~doi: 10.1108/JIABR-08-2020-0258

84. Karami A, Farokhzadian J, Foroughameri G. Nurses ' professional competency and organizational commitment : I~~i~~s it important for human resource management? PLoS One. 2017;12(11):e0187863. ~~doi:~~doi: ~~https://doi.org/~~10.1371/journal.pone.0187863 November

85. Ninglasari SY, Muhammad M. Zakat digitalization: effectiveness of zakat management in the ~~covid~~COVID-19 pandemic era. J Islam Econ Laws. 2021;4(1):26–44. ~~doi:~~doi: 10.23917/jisel.v4i1.12442

Formatted: Font: (Default) Times New Roman

Formatted: Font: (Default) Times New Roman

Appendix. Questionnaire

Integrating sustainable Islamic social finance: An Analytical Network Process using the Benefit Opportunity Cost Risk (ANP BOCR) framework: The case of Indonesia

This questionnaire aims to develop solutions and strategies to build a sustainable Islamic Social Finance integration model by considering the benefits, opportunity costs and risks (BOCR) of the model built. This questionnaire aims to collect primary data in order to develop the Analytical Network Process model. It is hoped that in this study the strategy can be developed using the Analytical Network Process.

This questionnaire is the primary data collection stage for the research "Sustainable Islamic Social Finance Model: Strategic Solution in Countering Adverse Impacts of Social Economy of The Covid-19 Using Analytical Network Process Benefit Opportunities Cost Risk. This questionnaire is designed to determine the personal assessment of key informants, which include practitioners, experts/experts, government representatives, academics, community leaders, and associations. There is no right or wrong answer, but this assessment aims to compare the level of importance or priority of each attribute to a problem. The rating scale will be explained as follows:

1 = Not important or not influential

2-3= Less important or less influential

4-5= Important or influential

6-7= Very important or very influential

8-9= Absolutely important or absolutely influential

SECTION 1: IMPORTANT ASPECT

1. Looking at the following aspects, **how important are these aspects in the integration of Islamic Social Finance?**
   a. Governance
   b. Sustainable financing
   c. Institutional
   d. HR Islamic Social Finance Institute
   e. Regulation
   f. Use of Technology

2. In your opinion, from the four aspects below, **which aspect has the highest priority compared to all aspects**?
   a. Benefit
   b. Opportunity
   c. Cost
   d. Risk

Formatted: Font: Not Bold, Italic
Formatted: Justified
Formatted: Font: Bold
Formatted: Font: (Default) Times New Roman
Formatted: Font: (Default) Times New Roman

SECTION 2: ANALYSIS OF BENEFIT, OPPORTUNITY, COST, RISK (BOCR)

BENEFIT

1. The following are several of **benefits from the governance aspect in supporting the creation of Islamic Social Finance integration**, how important are the benefits below in supporting the creation of Islamic Social Finance integration?
   a. There is an integration of Islamic social finance into commercial enterprises
   b. There is an MoU between BAZNAS and BWI to integrate zakat and waqf
   c. Many previous studies support the governance of zakat and waqf
   d. There are already standards for zakat and waqf management
   e. Some programs increase the motivation of donors to channel funds to Islamic social, financial institutions
   f. Every Islamic social finance institution has a supervisor so that the institution's operations run according to the plans and applicable rules
   g. The existence of a sharia supervisory board makes decisions more appropriate and following sharia rules
   h. Strengthening SDGs for Islamic social finance institutions will provide clear and measurable goals
   i. There are financial reporting standards for Islamic social financial institutions
   j. National data integration
   k. Increased effectiveness and efficiency in the use of operational funds

2. The following are several **benefits from the aspect of sustainable financing in supporting the creation of the integration of Islamic Social Finance**; how important are the benefits below in supporting the creation of the integration of Islamic Social Finance?
   a. The availability of cheap funds sourced from zakat, *infaq*, alms, waqf will make it easier for MSMEs to get financing
   b. Financing carried out at BMT includes social and financial functions commercial to increase the competitiveness of BMT
   c. There are many contracts in financing (*qard*, *ijarah*, *mudharabah*, etc.) that can reach a wider target recipient.

3. The following are several **benefits from the institutional aspect in supporting the creation of Islamic Social Finance integration**; how important are the benefits below in supporting the creation of Islamic Social Finance integration?
   a. The broad reach of each Islamic social financial institution spread across Indonesia
   b. The existence of branch offices or representative offices in various regions provides a broader range of benefits
   c. The ZISWAF Fund managed by the institution becomes more organized
   d. Islamic social finance institutions established by the community/social community have the potential to develop faster
   e. The increasing interest of donors to pay ZISWAF in institutions
   f. There is an association in Islamic social fund institutions

4. The following are several **benefits from the HR aspect in the Institution in supporting the creation of the integration of Islamic Social Finance**; how important are the benefits below in supporting the creation of the integration of Islamic Social Finance?
   a. Human resource loyalty in Islamic social fund management institutions occurs because of the high level of religiosity
   b. Increase professionalism of human resource

Formatted: Font: (Default) Times New Roman

Formatted: Font: (Default) Times New Roman

5. The following are several **benefits from the regulatory aspect in supporting the creation of Islamic Social Finance integration**; how important are the benefits below in supporting the creation of Islamic Social Finance integration?
   a. ZISWAF rules in Indonesia are more complete compared to other countries
   b. The regulations do not prohibit one institution from having a dual role, namely as a nadzir and amyl
   c. There is political support from the government executive board for the optimization of ZISWAF

6. The following are several **benefits of using technology in supporting the creation of the integration of Islamic Social Finance**; how important are the benefits below in supporting the creation of the integration of Islamic Social Finance?
   a. The use of technology makes it easier to raise and distribute funds
   b. Availability of platforms for national data integration so that data becomes accurate
   c. Access to information becomes faster and easier
   d. Technology provides a wider range of socialization

OPPORTUNITY

1. The following are several **opportunities from the governance aspect in supporting the creation of Islamic Social Finance integration**; how important are the opportunities below in supporting the creation of Islamic Social Finance integration?
   a. Achieved realization of the potential for collecting zakat and waqf in Indonesia
   b. The collaboration of ZISWAF funds with BMT will present optimal Islamic social finance management
   c. Improving the performance and effectiveness of Islamic social finance institutions
   d. The emergence of a more comprehensive performance appraisal
   e. The higher contribution of Islamic social finance in achieving the SDGs, especially the needs during the pandemic
   f. Increasing the image of Islamic social financial institutions
   g. Increased transparency of social financial management
   h. Availability of accurate information for stakeholders
   i. Increased public trust in Islamic social financial institutions
   j. Increased capacity building
   k. The ability of Islamic social financial institutions to carry out their duties and functions effectively and efficiently

2. The following are several **opportunities from the aspect of sustainable financing in supporting the creation of the integration of Islamic Social Finance**; how important are the opportunities below in supporting the integration of Islamic Social Finance?
   a. Ease of accessibility to Islamic social financial institutions, both in urban and rural areas
   b. The creation of a sustainable Islamic social finance ecosystem
   c. The level of funding needs is high, especially in the productive sector.
   d. Increased profit and income from financing
   e. Increased productivity of both the recipient of the financing or the funds used
   f. The decrease in the total poverty zone in Indonesia, which is in line with the SDGs

Formatted: Font: (Default) Times New Roman

Formatted: Font: (Default) Times New Roman

3. The following are several **opportunities from the institutional aspect in supporting the creation of Islamic Social Finance integration**; how important are the opportunities below in supporting the creation of Islamic Social Finance integration?
   a. The diversity of Islamic social financial institutions further optimizes ZISWAF funds
   b. The increasing number of Islamic social financial institutions spread to the lower levels
   c. The existence of Islamic social financial institutions or services to the lowest level, for example: villages, etc.

4. The following are several **opportunities from the HR aspect in the Institution in supporting the creation of Islamic Social Finance integration**; how important are the opportunities below in supporting the creation of Islamic Social Finance integration?
   a. Increasing the number of human resources who have competence in the management of Islamic social finance
   b. Increasing the competitiveness of institutional HR
   c. Availability of new job opportunities and increased absorption of labor

5. The following are a number of **opportunities from the regulatory aspect to support the creation of the integration of Islamic Social Finance**, how important are the opportunities below in supporting the creation of the integration of Islamic Social Finance?
   a. There are opportunities for regulatory development if needed
   b. Increased government support in innovative ZISWAF development and optimization of Islamic social finance
   c. Improved relations between OIC countries related to the management of Islamic social finance

6. The following are several **opportunities from using technology to support the integration of Islamic Social Finance**; how important are the opportunities below in supporting the creation of the integration of Islamic Social Finance?
   a. Increased public understanding of technology
   b. Increasing the effectiveness and efficiency of Islamic social finance management

COST

1. The following are several **costs on the aspects of governance needed in the integration of Islamic Social Finance**, how much are the costs below in the integration of Islamic Social Finance?
   a. There is a high-cost requirement to improve people's literacy
   b. Islamic social finance management requires high costs, one of which is related to infrastructure development, provision of tools, operational costs to monitoring systems
   c. The existence of the Sharia Supervisory Board (DPS) makes the bureaucratic flow take a long time
   d. The high cost of conducting a public audit
   e. Preparation of standardized reports takes quite a long time
   f. The gap between academics and practitioners in the practice of managing Islamic social finance
   g. Inefficient administration

2. The following are several **costs from the sustainable financing aspects needed in the integration of Islamic Social Finance**, how much are the costs below in the integration of Islamic Social Finance?
   a. High service fees for Islamic financing
   b. The position of Islamic social financial institutions is not strong, so they are very dependent on other intermediary institutions
   c. Public perception that Islamic financing still contains usury

Formatted: Font: (Default) Times New Roman
Formatted: Font: (Default) Times New Roman

d. High marketing costs for strengthening Islamic financing literacy in Islamic social financial institutions.

3. The following are several **costs from the institutional aspects needed in the integration of Islamic Social Finance**, how much are the costs below in the integration of Islamic Social Finance?
   a. Differences in the pattern of government-based and community/private-based institutions
   b. Islamic social finance management through an institutional approach requires high costs

4. The following are several **costs from the HR aspect in institutions that are needed to integrate Islamic Social Finance**; how much are the costs below in the integration of Islamic Social Finance?
   a. Training for HR requires a large amount of money
   b. Mismatch in the background of the manager of Islamic social financial institutions
   c. Low knowledge of human resources in the field of financial management
   d. Limited HR mobility during the Pandemic

5. The following are several **costs from the regulatory aspect in the integration of Islamic Social Finance**, how much are the costs below in integrating Islamic Social Finance?
   a. Limitations of guidance in the management of ZISWAF when the Act is issued
   b. Differences in permits for Islamic social finance management institutions
   c. Conflict handling costs
   d. Conflict of interest between stakeholders
   e. The integration of ZISWAF management takes a long time due to political factors

6. The following are several **costs from the aspect of using technology needed in the integration of Islamic Social Finance**, how much is the cost below in the integration of Islamic Social Finance?
   a. Implementation of digital technology requires high costs
   b. The use of technology will provide challenges related to the fulfillment of sharia principles

RISK

1. The following are several **risks from the governance aspect in integrating Islamic Social Finance**; how influential are the risks below in the integration of Islamic Social Finance?
   a. There is mismanagement in the implementation of the functions of Islamic social financial institutions
   b. Islamic social finance institutions have not adopted all the principles in governance
   c. There is potential for miscommunication both internally and externally by Islamic social financial institutions
   d. Mis-Coordination related to the division of tasks and roles
   e. Differences in work patterns and culture between institutions
   f. Incompatibilities of the supervisory board
   g. Supervision is not carried out thoroughly
   h. Reports that are not representative and less accurate
   i. There is potential for non-compliance with regulations, both formal rules and sharia rules in Islamic social finance governance
   j. The achievement of several SDGs points is contradictory to the essence of the function of Islamic social finance institutions
   k. The 17 goals in the SDGs are not all relevant to the ZISWAF goals

2. The following are several **risks from the sustainable financing aspect in the integration of Islamic Social Finance**, how influential are the risks below in integrating Islamic Social Finance?
   a. There is liquidity risk for financial institutions

Formatted: Font: (Default) Times New Roman

Formatted: Font: (Default) Times New Roman

b. The absence of guarantees creates a moral hazard problem
c. Lack of motivation of recipients of Islamic social finance to develop
d. Failure in carrying out financing
e. Time is needed in conditioning the synchronization between ziswaf institutions and the government regarding the legality of the law
f. Literacy related to Islamic social funds is still far from expected
g. High refusal to use waqf funds to lend

3. The following are several **risks from the institutional aspect in integrating Islamic Social Finance**; how influential are the risks below in integrating Islamic Social Finance?
   a. The institutional model is not flexible in accommodating problems
   b. Institutional fund management will limit the space for innovation.

4. The following are several **risks from the HR aspect in institutions in the integration of Islamic Social Finance**, how influential are the risks below in the integration of Islamic Social Finance?
   a. High employee turnover rate
   b. The profession of nazhir and/or amil is not considered as the primary profession
   c. Increasing digitalization will replace the role and number of HR

5. The following are several **risks from regulatory aspects in integrating Islamic Social Finance**; how influential are the risks below in the integration of Islamic Social Finance?
   a. There are no mandatory rules
   b. There is a period for regulatory amendments, so regulations are less flexible in responding to current conditions
   c. There are political challenges in strengthening regulations
   d. Change of leader replaces policy direction
   e. Conflicts of interest that arise between policymakers

6. The following are several **risks from using technology in the integration of Islamic Social Finance**; how influential are the risks below in the integration of Islamic Social Finance?
   a. There is a crime in the implementation of technology
   b. There is a potential for non-fulfillment of sharia principles with the use of technology

SECTION 3: SOLUTION AND STRATEGIES IN INTEGRATING ISLAMIC SOCIAL FINANCE

1. The following are several **solutions in integrating Islamic Social Finance**; how important are the solutions below in the integration of Islamic Social Finance?
   a. Financial integration in providing funding to beneficiaries through the distribution of zakat, waqf, and Islamic microfinance based on the beneficiaries' economic circumstances and capabilities
   b. Integrating ISF with state fiscal instruments, which is supported by existing regulations
   c. Integration through non-formal coordination between ISF institutions
   d. Integration among Islamic social finance institutions based on capacity building and role division
   e. Establishing an integrated national data center for all ISF instruments
   f. Integration based on regional clusters with a solid authority
   g. Integrating ISF through the passage of new laws and regulations
   h. Integration based on ISF management clusters, such as zakat *Amils* joining other zakat institutions and waqf manager joining other waqf institutions

Formatted: Font: (Default) Times New Roman
Formatted: Font: (Default) Times New Roman

2. The following are several **strategies in integrating Islamic Social Finance**; how important are the strategies below in the integration of Islamic Social Finance?
   a. Improve the quality of human resources through training and certification
   b. Promotes the availability of real-time data through database integration and reporting
   c. Optimizing the digital technology in Islamic social finance instruments collection, management, and distribution process
   d. Coordination and synergy among stakeholders (e.g., practitioners, regulators, associations, and academicians) to establish shared commitment and vision
   e. Encouraging the broadening of the range of benefits by establishing service units at lower levels, such as service units at the district, village, and mosque levels, as well as strategic locations that support the broadening of the reach of the benefits of ISF instruments
   f. Establishing a new institution that is capable of integrating all instruments of ISF
   g. Realizing the government's role in supporting the integration of ISF through regulation and socialization
   h. Developing the ISF institutions to have two functions, namely, *amil* and *nazir*
   i. Revising and updating all laws and regulations governing the management of Islamic social finance instruments to accommodate the development of ISF instruments

1. Arikan G, Bloom PB-N. "I was hungry and you gave me food": Religiosity and Attitudes Toward Redistribution. PLoS One [Internet]. 2019;14(3):e0214054. Available from: https://doi.org/10.1371/journal. pone.0214054
2. Jouti AT. An Integrated Approach for Building Sustainable Islamic Social Finance Ecosystems. ISRA Int J Islam Financ. 2019;11(2):246–66.
3. Sulistyowati. Designing Integrated Zakat-Waqf Models for Disaster Management. J Islam Monet Econ Financ. 2018;4(2):347–68.
4. Kamaruddin MIH, Auzair SM. Integrated Islamic Financial Accountability Model for Islamic Social Enterprise (ISE). J Muamalat Islam Finance Res. 2019;16(1):17–36.
5. Adjar H, Chakir A, Meskine L El. Exploratory Study of Islamic Social Finance in Morocco. Int Rev Entrep Financ [Internet]. 2020;3(2):45–69. Available from: https://revues.imist.ma/index.php/IREF/article/view/25539
6. Arumsari V, Rikawati R. Islamic Social Funds: Enabling the Unable To Be Able. J Appl Bus Adm. 2020;4(1):88–97.
7. Hudaefi FA. How does Islamic fintech Promote the SDGs? Qualitative Evidence from Indonesia. Qual Res Financ Mark. 2020;12(4):353–66.
8. Kasri RA. Management of Zakah Distribution: Empirical Evidence From Indonesia. AFEBI Islam Finance Econ Rev. 2017;
9. Kailani N, Slama M. Accelerating Islamic Charities in Indonesia: Zakat, Sedekah and the Immediacy of Social Media. South East Asia Res [Internet]. 2020;28(1):70–86. Available from: https://doi.org/10.1080/0967828X.2019.1691939
10. Bilo C, Machado AC. The Role of Zakat in the Provision of Social Protection: A Comparison between Jordan and Sudan. Int J Sociol Soc Policy. 2020;40(3–4):236–48.
11. Manurung S. Islamic Religiosity and Development of Zakat Institution. Qudus Int J Islam Stud. 2013;1(2):197–220.

Formatted: Font: 12 pt

Formatted: Normal, Indent: Left: 0.1", Hanging: 0.3", Space Before: 6 pt, After: 6 pt

Formatted: Font: (Default) Times New Roman

Formatted: Font: (Default) Times New Roman

Formatted: Font: (Default) Times New Roman

12. Azha L, Baharuddin S, Sayurno, Salahuddin SS, Afandi MR, H. HA. The Practice and Management of Waqf Education in Malaysia. In: Procedia - Social and Behavioral Sciences. Elsevier B.V.; 2013. p. 22–30.
13. Widiastuti T, Cahyono EF, Zulaikha S, Mawardi I, Al Mustofa MU. Optimizing Zakat Governance in East Java using Analytical Network Process (ANP): The Role of Zakat Technology (ZakaTech). J Islam Account Bus Res. 2021;12(3):301–19.
14. Raimi L, Patel A, Adelopo I. Corporate social responsibility, Waqf system and Zakat system as faith-based model for poverty reduction. World J Entrep Manag Sustain Dev. 2014;10(3):228–42.
15. Jermsittiparsert K, Sommanawat K. The contributions of strategic Zakat in supply chain and profitability of the National Zakat Agency: A case study from National Zakat Agency Indonesia. Humanit Soc Sci Rev. 2019;7(2):471–8.
16. Cizakca M. Awqaf in History and Its Implications for Modern Islamic Economies. Islam Econ Stud [Internet]. 1998;6(1):43–70. Available from: https://ssrn.com/abstract=3164811
17. Khairi KF, Aziz MRA, Laili NH, Nooh MN, Sbri H, Basah MYA. Human and Economic Development through Share WAQF (WAQF Corporate): The Case of GLCS in Malaysia. Life Sci J [Internet]. 2014;11(x):104–10. Available from: http://www.lifesciencesite.com
18. Haneef MA, Huq A, Mohammed MO, Amin FB. Integration of Waqf and Islamic Microfinance for Poverty Reduction: A Survey in Kuala Selangor, Malaysia. J Islam Financ. 2013;2(2):1–16.
19. Von Pischke JD. Measuring the Trade-off Between Outreach and Sustain-ability of Microenterprise Lenders. J Int Dev. 1996;8(2):225–39.
20. Chow TC, Zailani S, Rahman MK, Qiannan Z, Bhuiyan A, Patwary AK. Impact of Sustainable Project Management on Project Plan and Project Success of the Manufacturing Firm: Structural Model Assessment. PLoS One [Internet]. 2021;16(11):e0259819. Available from: http://dx.doi.org/10.1371/journal.pone.0259819
21. Brau JC, Woller GM, Brau JC, Woller GM. Microfinance: A comprehensive review of the existing literature. J Entrep Financ. 2004;9(1):1–28.
22. Ali AFM, Rashid ZA, Johari F, Aziz MRA. The effectiveness of Zakat in reducing poverty incident: An analysis in Kelantan, Malaysia. Asian Soc Sci. 2015;11(21):355–67.
23. Ahmed BO, Johari F, Wahab KA. Identifying the Poor and the Needy Among the Beneficiaries of Zakat Need for a Zakat-based Poverty Threshold in Nigeria. Int J Soc Econ. 2017;44(4):446–58.
24. Rosli MR Bin, Salamon H Bin, Huda M. Distribution management of zakat fund: Recommended proposal for asnaf riqab in Malaysia. Int J Civ Eng Technol. 2018;9(3):56–64.
25. Bin-Nashwan SA, Abdul-Jabbar H, Aziz SA, Haladu A. Zakah compliance behavior among entrepreneurs: economic factors approach. Int J Ethics Syst. 2020;36(2):285–302.
26. Taha R, Adam F, Ali NNM, Ariff AM. Religiosity and Transparency in the Management of Zakat Institutions. J Leg Ethical Regul Issues. 2017;20(1):1–9.

Formatted: Font: (Default) Times New Roman

Formatted: Font: (Default) Times New Roman

27. Chowdhury MSR, Ghazali MF bin, Ibrahim MF. Economics of Cash WAQF management in Malaysia: A proposed Cash WAQF model for practitioners and future researchers. African J Bus Manag. 2011;5(30):12155–63.

28. Bassem I, Ousman J, Ansar A, Hamidar. Pathway of Welfare and Prosperity : Does Zakat Implementation Reach Indonesian To Economic Sustainability ? J Internet Bank Commer. 2019;24(1):1–13.

29. Ahmad M. An Empirical Study of The Challenges Facing Zakat and Waqf Institutions in Northern Nigeria. ISRA Int J Islam Financ. 2019;11(2):338–56.

30. Ali MM, Devi A, Furqani H, Hamzah H. Islamic Financial Inclusion Determinants in Indonesia: An ANP Approach. Int J Islam Middle East Financ Manag. 2020;13(4):727–47.

31. Mustofa I, Santoso D, Rosmalinda U. The Implementation of the Regulation of Cash Waqf Management in Higher Educational Institution in Indonesia and Malaysia (a Study of Legal System Theory). Humanit Soc Sci Rev. 2020;8(4):69–77.

32. Pitchay AA, Thaker MAMT, Mydin AA, Azhar Z, Latiff ARA. Cooperative-waqf Model: A Proposal to Develop Idle Waqf Lands in Malaysia. ISRA Int J Islam Financ. 2018;10(2):225–36.

33. Sukmana R. Critical Assessment of Islamic Endowment Funds (Waqf) Literature: Lesson For Government and Future Directions. Heliyon [Internet]. 2020;6(10):e05074. Available from: https://doi.org/10.1016/j.heliyon.2020.e05074

34. IRTI. Islamic Social Finance Report 2014. Islam and the Moral Economy. 2014.

35. Purbasari I, Fauzan EM, Azizah. Zakat in Indonesian State Treasury Funds: Its Potential Obstacle and Abuse. IOP Conf Ser Earth Environ Sci. 2018;175:1–6.

36. Halimatusa'diyah I. Zakat and Social Protection: The Relationship Between Socio-religious CSOs and the Government in Indonesia. J Civ Soc. 2015;11(1):79–99.

37. Balassa B. Economic Integration. World Econ. 1991;142:35–52.

38. Zauro NA, Saad RAJ, Sawandi N. Enhancing socio-economic justice and financial inclusion in Nigeria: The role of zakat, Sadaqah and Qardhul Hassan. J Islam Account Bus Res. 2020;11(3):555–72.

39. Hassanain KM. Integrating Zakah, Awqaf, and IMF for Poverty Allevation: Three Models of Islamic Micro Finance. J Econ Soc Thought [Internet]. 2015;2(2):106–20. Available from: https://e-resources.perpusnas.go.id:2350/docview/1873027697/10B524F6CCAD47DAPQ/5?accountid=25704

40. Haneef MA, Pramanik AH, Mohammed MO, Bin Amin MF, Muhammad AD. Integration of Waqf-Islamic Microfinance Model for Poverty Reduction: The Case of Bangladesh. Int J Islam Middle East Financ Manag. 2015;8(2):246–70.

41. Ambrose AHAA, Hassan MAG, Hanafi H. A Proposed Model For Waqf Financing Public Goods and Mixed Public Goods in Malaysia. Int J Islam Middle East Financ Manag. 2018;11(3):395–415.

42. Shalleh MCM, Razali SS, Laksana NNM, Embi NAC, Abdullah NI. Developing a sustainable model of Waqf-based Takaful for flood victims in Malaysia. J Islam Account Bus Res. 2020;11(9):1941–52.

Formatted: Font: (Default) Times New Roman

Formatted: Font: (Default) Times New Roman

43. Saaty TL. Decision Making with the Analytic Network Process. In: International Series in Operations Research & Management Science. Springer; 2013. p. 1–40.

44. Saaty TL, Vargas LG. Decision Making with the Analytic Network Process: Economic, Political, Social and Technological Application with Benefit, Opportunities, Cost and Risks. II. Vol. 195, International Series in Operations Research and Management Science. London: Springer; 2006. 1–370 p.

45. Ascarya. Analytic Network Process (ANP): Pendekatan Baru Studi Kualitatif. Cent Cent Bank Educ Stud Bank Indones. 2005;

46. Zammori F, Bigliardi B, Carmignani G. Selecting Inbound Logistic Policies: An ANP-based Multi Criteria Decision Making Approach. Int J Logist Syst Manag. 2014;19(2):212–44.

47. Munandar A, Syakhroza A, Martani D, Siswantoro D. Does regulation increase zakat payment? Acad Account Financ Stud J. 2019;23(6).

48. Sulaiman M, Alhaji Zakari M. Financial Sustainability of State Waqf Institutions (SWIs) in Malaysia. J Islam Account Bus Res. 2019;10(2):236–58.

49. Adiwijaya ZA, Suprianto E. Good Governance of Zakat Institutions: A Literature Review. J Southwest Jiaotong Univ. 2020;55(2):1–7.

50. Minister of Law and Human Rights Republic of Indonesia. Undang-Undang Republik Indonesia Nomor 23 Tahun 2011 Tentang Pengelolaan Zakat. 2011.

51. Sarif SM, Ismail Y. Influence of Ta'awun in Managing Stickiness of Zakat Institutions. Int J Zakat Islam Philanthr. 2020;2(2):122–8.

52. Nurlita E, Ekawaty M. The Direct and Indirect Effect of Zakat on the Household Consumption of Mustahik (A Study of Zakat Recipients from BAZNAS Probolinggo Municipality). Int J Zakat [Internet]. 2018;3(2):41–56. Available from: https://doi.org/10.37706/ijaz.v3i2.77

53. Taufiq IF, Kusnendi K, Nurasyiah A. The Effect of Productive Zakat , Business Experience , and Mentoring on Farmers ' Revenues (Survey on Lumbung Desa Program by Sinergi Foundation in Cibaeud Village , Cigalontang District , Tasikmalaya Regency). Int J Zakat. 2018;3(3):55–67.

54. Azganin H, Kassim S, Auwal Adam S. Proposed waqf crowdfunding models for small farmers and the required parameters for their application. Islam Econ Stud. 2021;

55. Thaker MAMT, Amin MF, Thaker HMT, Khaliq A, Pitchay AA. Cash Waqf Model for Micro Enterprises' Human Capital Development. ISRA Int J Islam Financ. 2021;13(1):66–83.

56. Sari DF, Beik IS, Rindayati W. Investigating the Impact of Zakat on Poverty Alleviation : A Case from West Sumatra, Indonesia. Int J Zakat [Internet]. 2019;4(2):1–12. Available from: https://doi.org/10.37706/ijaz.v4i2.180

57. Widiastuti T, Auwalin I, Rani LN, Mustofa MU Al. A Mediating Effect of Business Growth on Zakat Empowerment Program and Mustahiq's Welfare. Cogent Bus Manag [Internet]. 2021;8(1):1882039. Available from: https://doi.org/10.1080/23311975.2021.1882039

58. Hamed MM. The Role of Islamic Social Finance in Mitigating Humanitarian Crises; A Multi-Range Strategy to Mitigate COVID-19 Impacts. Eur J Islam Financ [Internet]. 2020;0(16):1–10. Available from: http://www.ojs.unito.it/index.php/EJIF

Formatted: Font: (Default) Times New Roman

Formatted: Font: (Default) Times New Roman

59. Afriadi F, Sanrego yulizar d. Mengurai Permasalahan Distribusi Zakat Kepada Fakir Miskin: Pengalaman Organisasi Pengelola Zakat Baznaz, Dompet Dhuafa, Dan Lazizmu. J Madania. 2016;20(1):23–36.

60. Arwani A. Sharia Accounting on Indonesian Financial Accounting Standard on Zakat and Waqf Take on Industrial Revolution 4.0 and Society Era 5.0. J Islam Account Financ Res. 2020;2(2):229–58.

61. Hudaefi FA, Caraka RE, Wahid H. Zakat Administration in Times of COVID-19 Pandemic in Indonesia: A Knowledge Discovery via Text Mining. Int J Islam Middle East Financ Manag. 2021;

62. Mutamimah M, Alifah S, Gunawan G, Adnjani MD. ICT-Based Collaborative Framework for Improving the Performance of Zakat Management Organisations in Indonesia. J Islam Account Bus Res. 2021;

63. Saad RAJ, Farouk AU. A comprehensive review of barriers to a functional Zakat system in Nigeria: What needs to be done? Int J Ethics Syst. 2019;35(1):24–42.

64. Saad RAJ, Idris KM, Shaari H, Sawandi N, Derashid C. Governance of non-profit organizations: A case of zakat institutions in Malaysia. Int J Econ Res. 2017;14(16):253–65.

65. Mohd Thas Thaker MA, Mohd Thas Thaker H, Allah Pitchay A. Modeling crowdfunders' behavioral intention to adopt the crowdfunding-waqf model (CWM) in Malaysia: The theory of the technology acceptance model. Int J Islam Middle East Financ Manag. 2018;11(2):231–49.

66. Muneeza A, Nadwi S. The Potential of Application of Technology-Based Innovations for Zakat Administration in India. Int J Zakat [Internet]. 2019;4(2):87–100. Available from: https://doi.org/10.37706/ijaz.v4i2.191

67. Usman H, Mulia D, Chairy C, Widowati N. Integrating Trust, religiosity and image into technology acceptance model: the case of the Islamic philanthropy in Indonesia. J Islam Mark. 2020;

68. Berakon I, Aji HM, Hafizi MR. Impact of Digital Sharia Banking Systems on Cash-Waqf among Indonesian Muslim Youth. J Islam Mark. 2021;

69. Mohd Nor S, Abdul-Majid M, Esrati SN. The role of blockchain technology in enhancing Islamic social finance: the case of Zakah management in Malaysia. Foresight. 2021;23(5):509–27.

70. Karami A, Farokhzadian J, Foroughameri G. Nurses ' Professional Competency and Organizational Commitment : Is it Important for Human Resource Management? PLoS One [Internet]. 2017;12(11):e0187863. Available from: https://doi.org/10.1371/journal.pone.0187863 November

71. Hidayatullah A, Priantina A. Toward Zakat Management Integration in Indonesia: Problems and Solution. Ahkam J Ilmu Syariah. 2018;18(2):321–46.

72. Ninglasari SY, Muhammad M. Zakat Digitalization: Effectiveness of Zakat Management in the Covid-19 Pandemic Era. J Islam Econ Laws. 2021;4(1):26–44.

Formatted: Font: (Default) Times New Roman

Formatted: Font: (Default) Times New Roman

Formatted: Font: (Default) Times New Roman

Author's Responses to the Reviewer Comments

Paper ID	PONE-D-21-31476
TITLE	**Integrating Sustainable Islamic Social Finance: An Analytical Network Process using the Benefit Opportunity Cost Risk (ANP BOCR) Framework: The Case of Indonesia**

No.	Comments from Reviewer (s)	Action	Page	Paragraph
1.	Before the objective chapter, provide background on the problem related to the topic discussed because the goal is to answer the existing problems. It is necessary to briefly explain what is meant by a philanthropic institution and how does it relate to the topic of discussion?	Thank you for the advice.<br><br>a. We have added the problem in the abstract (purpose).<br><br>b. Regarding "philanthropic institution", we replaced the word with the word "institutional aspect" because it is easier to understand the integration of Islamic social finance	1	Abstract
2.	Explain how to determine the sample used by the author	Thank you for the comments<br><br>a. In the abstract section, we add the following sentence to explain how to determine the sample used "The respondents were selected for their expertise and experience in the selected topic"<br><br>b. In method section, the purposive sampling technique	1<br><br>10	Abstract<br><br>Method section, in "Data and research samples"

No.	Comments from Reviewer (s)	Action	Page	Paragraph
		aims to select respondents. There are three criteria to select respondent		paragraph 3
3.	Add to keyword: priority strategy	Thank you for the advice.<br><br>We have added those keywords.	1	Abstract
4.	The author needs to describe the role of Islamic social finance in a broader perspective, not only in Muslim countries, or limited to the COVID-19 pandemic. The illustration can be expanded not only to the problem of the Covid-19 Pandemic (monetary crisis or global economic crisis, global food and financial crisis).	Thank you for your comment<br>.<br><br>We have revised this section by adding:<br><br>1. The role of Islamic social finance in general (before covid)<br>2. the role of Islamic social finance in several non-majority Muslim countries	1	Introduction paragraph 1
5.	The author needs to explain what are other supporting factors besides lack of optimal governance, that can be the reasons for the vast gap between the potential of ISF and the reality? Complete with another relevant literature review (LR), such as aspects of Islamic fundraising/finance, management, transparency, mutual trust, etc.	Thank you for your comments<br><br>We have added several factors that cause the vast gap between the potential of Islamic social finance.<br><br>Some of these factors are:<br>1. Lack of technology in the management<br>2. Lack of the ability and competence of the manager<br>3. Lack of synergy<br>4. Lack of trust and public literacy<br><br>We support these factors with various reputable journals.	2-3	Introduction paragraph 4
6.	First, explain what ISF governance is, then	Thank you for the advice	3	Introduction paragraph 5

No.	Comments from Reviewer (s)	Action	Page	Paragraph
	explain its aspects.	We have added an explanation of ISF governance.		
7.	Please cite more fully and in detail regarding policies and regulations that do not support it, as well as what policy solutions can be offered?	Thank you for the comments<br><br>We've added explanations regarding unsupported policies, which include:<br>a. according to regulations, Islamic social finance instruments are still voluntary, so there is a gap in realization and potential for the collection<br>b. there are no sanctions for those who do not pay zakat there are different regulations between Islamic social finance instruments	3	Introduction paragraph 6
8.	Provide an explanation of the current state of sustainability of ISF along with references from previous research. If there is no review on this matter, please provide a reference that strengthens your statement that most of the empowerment programs sourced from the ISF do not meet the sustainability aspect.	Thank you for the review<br><br>Regarding statement that strengthens sustainability of empowerment programs, we apologize that we could not find any journals that support this statement.<br><br>However, we wrote this statement based on the practice that occurred in the field and the results of the focus group discussions that we conducted with experts in Islamic social finances, especially zakat and waqf.	3	Introduction paragraph 8
9.	Add the challenge from the side of	Thank you for the advice	3	Introduction paragraph 8

No.	Comments from Reviewer (s)	Action	Page	Paragraph
	Muslims who oppose the ISF being regulated by the government because Indonesia is based on Pancasila, not a country with Islamic law, so its population consists of various beliefs and religions. There could be a sense of injustice if the government only regulates and takes money from Muslims to help government finances	We have added the challenge to strengthen our introduction.		
10.	Literature Review for contemporary Islamic finance needs to be enriched especially from reputable global indexed primary journals, covering Islamic banking, Islamic insurance, etc.	Thank you for the advice<br><br>We have added reputable journals related to contemporary Islamic social finance.	4	Literature review, sub section "The concept of Islamic social finance and its instrument" paragraph 1
11.	Regarding sentences "Further, the Islamic social finance instruments are still partially Managed", Is this sentence incomplete or imperfect? Correct this sentence so that it is easier to understand	Thank you for your review<br><br>We have revised this sentence	6	Section of Literature review, sub section "Issues with Islamic social finance integration", paragraph 1
12.	Regarding sentences "Given the challenges which consequently create a significant gap between the potential and actualization of Islamic social finance management and accounting for the enormous potential of Islamic social finance, it is necessary to integrate Islamic Social Finance instruments on an ongoing basis to achieve optimum level of management.", In Scientific Writing, avoid in one paragraph consisting of only one sentence	Thank you for your review<br><br>We have revised this sentence	6	Section of Literature review, sub section "Issues with Islamic social finance integration", paragraph 1

No.	Comments from Reviewer (s)	Action	Page	Paragraph
13.	The importance of including Literature Review about integration aspects of growth, aspects of justice and aspects of sustainability	Thank you for the review.<br><br>We have revised this section as directed. We also include citations from reputable journals to support this revision.	7	Literature review sub section "Integration model of Islamic social finance" paragraph 1
14.	Mention what criteria are meant?	Thank you for the comments<br><br>Criteria that we mean is construct. In ANP, there is a construct to build the ANP model framework. Construct consists of six aspects, solutions, and strategies that we develop.	7	Method, sub section "research method and analysis approach" paragraph 1
15.	The author needs to explain some of the criteria used as input in this research using the two-level ANP method and why the BOCR analysis was selected	Thank you for the review<br><br>We have explained the reason we use two-level ANP (BOCR analysis) in the method.<br><br>We use two-level ANP (BOCR analysis) to analyze solutions and strategies that can be implemented in the short term and the most priority in integrating Islamic social finance by analyzing every benefit, opportunity, cost, and risk of each integration aspect.	7-8	Method, sub section "Research method and analysis approach" paragraph 1 line 8-13
16.	In sentences "literature review was conducted as part of this study to strengthen the ANP model that was Developed", Mention the Literature Review focused on what aspects	Thank you for your comments<br><br>The literature review in our method is used to strengthen the ANP model and collect problems, solutions, and	7-8	Method, sub section "Research method and analysis approach" paragraph 2

No.	Comments from Reviewer (s)	Action	Page	Paragraph
	?	strategies related to optimizing Islamic social finance.<br><br>We have added the explanation in the manuscript.		
17.	In sentences "Human resources play a crucial role in Islamic Social Finance management", What is the important role in this case? Give a brief explanation	Thank you for the advice<br><br>We have added explanations regarding the urgency and definition of each aspect we analyze	11	Result sub section "Priority aspect" paragraph 2
18.	What institutional aspects are the focus of this article (structure, function/role, coordination system, business partnerships with other parties).	Thank you for the review<br><br>We focus on the structure and coordination system. And we have added the explanation in our manuscript.	11-12	Result, sub section "Priority aspect" paragraph 2
19.	About integration between Islamic social finance institutions based on capacity development and division of roles, what types of integration are meant (horizontal integration, vertical integration or horizontal coordination, vertical coordination)? Please explain	Thank you for your comments<br><br>The integration is done vertically and horizontally. We have added it to our manuscript.	12	Result, sub section "Priority solution in integrating sustainable Islamic social finance" paragraph 1
20.	Explain what is the difference between short-term and long-term priority solutions shown in table 4 based on the expert agreement?	Thank you for the advice.<br><br>We have added an explanation of the differences between short-term and long-term priority solutions in our manuscript.<br>Short term solution considers the marginal cost of the benefits obtained. On the contrary, in the long term, the	13	Result, sub section "Priority solution in integrating sustainable Islamic social finance" paragraph 2

No.	Comments from Reviewer (s)	Action	Page	Paragraph
		priority is achieved when the total number of benefits and opportunities are obtained after reducing the total cost and risk (Saaty & Vargas, 2006; Zammori et al., 2014).<br><br>On the other hand, an expert judgment does not consider the benefits, opportunity, cost, and risk aspects of each solution formulated in this study.		
21.	Regarding sentences “According to the data processing results, the first and second priorities in the short and long- term are in the same order. The solutions that will be implemented first and foremost in the short and long term are financial integration in providing funding to beneficiaries and integration of Islamic social finance with state fiscal policy. According to expert judgment, integrating national data centers is the highest priority solution, with financial integration in providing funding to beneficiaries coming in second.”, It is necessary to conduct a more in-depth analysis and based on this analysis, it is synthesized by the author	Thank you for your comment<br><br>We provide arguments in the discussion section	15	Discussion, sub section “Priority solution in developing a sustainable Islamic social finance integration model”
22.	Regarding sentences “cryptocurrency waqf”, What is the equivalent in Syar'i or Arabic?	Thank you for the comments<br><br>We reviewed the cryptocurrency waqf and its relevance to Sharia. Then, we decided not to include this type of waqf	15	Result sub section “Priority strategies in integrating sustainable Islamic social finance” paragraph 1

No.	Comments from Reviewer (s)	Action	Page	Paragraph
		in our manuscript.		
23.	Regarding sentences “The order of priority strategies is shown in table 6, which displays short-term, long-term priority solutions and expert judgment. The strategies are summarized in table 7 as follows”, Give a brief description before referring to tables 6 and 7. In scientific writing, avoid one paragraph consisting of only one sentence.	Thank you for the advice<br><br>We've revised this sentence by giving a brief description	14	Result, sub section “Priority strategies in integrating sustainable Islamic social finance”, paragraph 2
24.	Regarding sentences “The results show that the short and long-term strategies are in the same order. Improving the quality of human resources, establishing real-time database integration and reporting, and optimizing the use of digital technology, become the priority strategies. However, the expert judgment put the optimization use of digital technology to be the priority strategy.”, What is the argument for each of the first and second statements, give a brief analysis or review.	Thank you for your comment<br><br>We provide arguments for the first and second statements in the discussion section<br><br>a. Optimizing the use of digital technology<br><br>b. Improving the quality of human resource	17<br><br><br><br><br>17<br><br>17	Discussion, sub section “strategic solution in developing a sustainable Islamic social finance integration model”<br><br>Paragraph 1<br><br>Paragraph 2-3
25.	Provide adequate arguments, in the financing program covered by sharia financing, success is determined by aspects of distribution, use or utilization (consumptive, productive economy), returns, and governance	Thank you for the advice<br><br>We have added an adequate argument about program financing.<br><br>We add arguments that include:<br>1. Relation of financing integration with existing integration theory<br>2. Reputable journals which	15	Discussion, sub section “Priority solution in developing a sustainable Islamic social finance integration model” paragraph 1

No.	Comments from Reviewer (s)	Action	Page	Paragraph
		shows the importance of distribution in zakat management<br>3. Reputable journals which shows that financing from Islamic social, financial instruments has a positive impact on the welfare of the poor		
26.	What are the operational steps to create a legal environment?	Thank you for the comments<br><br>In our opinion, there are several steps to create a legal environment which at least includes: government must revise the regulation, the government must socialize the revised laws and regulations to all stakeholders, encourage Islamic social finance management institutions to implement the law by offering rewards or incentives that the Institution can use, such as free training and accompaniment.	17	Discussion sub section “Priority solution in developing a sustainable Islamic social finance integration model” paragraph 3
27.	Type of technology, technology level, and what technology transfer and adoption processes will be implemented in managing the instrument of ISF	Thank you for the comments<br><br>We added an explanation regarding the type of technology.<br><br>There are at least four types of technology that must be implemented in the integration of Islamic social finance, which consist of:	17	Discussion, sub section “Strategic priorities in developing a sustainable Islamic Social finance integration model” paragraph 1

No.	Comments from Reviewer (s)	Action	Page	Paragraph
		a. technology for creating a centralized database linked to the government and Islamic social finance supervisors<br>b. technological advancements in supervision and monitoring<br>c. technology in the marketing component to aid in the collection of target achievement<br>d. technology to generate real-time management reports		
28.	Aspects of human resources that must be improved are technical skills and managerial capabilities. Which of these two aspects is dominant depends on their respective position.	Thank you for the comment.<br><br>We think that both skills (technical and management) are equally important. Technical skills are essential for staff to do administrative work and empowerment.<br><br>Meanwhile, management skills are needed by leaders or managers in making strategic ISF management decisions.	18	Managerial relevancy paragraph 1
29.	Acknowledgments should also be conveyed to someone (individual) who played an important role in writing this article, but is not listed as a member of the writing team.	Thank you for the comments<br><br>We did not name anyone involved in this study. However, we mention all representatives of academics, regulators, associations, and community leaders involved in this research for their support in the FGDs,	20	Acknowledgement paragraph 1

No.	Comments from Reviewer (s)	Action	Page	Paragraph
		in-depth interviews, and filling out our questionnaires.		
