# Supplementary material for: Integrating sustainable Islamic social finance: An Analytical Network Process using the Benefit Opportunity Cost Risk (ANP BOCR) framework: The case of Indonesia
Source: PLoS One. 2022 May 26;17(5):e0269039. doi: 10.1371/journal.pone.0269039 (PMC9135187; doi:10.1371/journal.pone.0269039)
Supplement: Attachment — Submitted filename: PONE-D-21-31476_Review_1.docx [file pone.0269039.s002.docx]

PLOS ONE

**Integrating Sustainable Islamic Social Finance: Analytical Network Process using Benefit Opportunity Cost Risk (ANP BOCR) Framework: The Case of Indonesia**

--Manuscript Draft--

| **Manuscript Number:** | PONE-D-21-31476 |
| --- | --- |
| **Article Type:** | Research Article |
| **Full Title:** | Integrating Sustainable Islamic Social Finance: Analytical Network Process using Benefit Opportunity Cost Risk (ANP BOCR) Framework: The Case of Indonesia |
| **Short Title:** | Integrating Sustainable Islamic Social Finance using ANP BOCR Framework |
| **Corresponding Author:** | Anidah Robani  Universiti Teknikal Malaysia Melaka Durian Tunggal, Melaka MALAYSIA |
| **Keywords:** | Islamic Social Finance, Financial Sustainability, Integration, Islamic Economy, Analytical Network Process, BOCR |
| **Abstract:** | Purpose – This research analyzes solutions and priority strategies for integrating sustainable Islamic Social Finance that could be implemented in the short and long term by analyzing it through the Benefit, Opportunity, Cost, and Risk (BOCR) framework, which comprises of six aspects: Governance, Sustainable Financing, philanthropic Institutions, Human Resources, Regulations, and Technology.  Design/methodology/approach – This qualitative research employs the Analytic Network Process method using Benefit, Opportunity, Cost, and Risk analysis. Data obtained mainly through focus group discussions and in-depth interviews with experts from academics, practitioners, associations, regulators, and community leaders. The data is processed with Microsoft excel and Super Decision software.  Findings – There are several findings worth considering. First, the highest priority integrating Islamic social finance aspects are human resources (0.97), regulation (0.86), and technology (0.76). Second, based on a short and long-term analysis, financial integration through sustainable financing (0.01 and 1.44) and improving human resource quality through certification and training (0.01 and 1.56) is a priority solution and strategy to integrate sustainable Islamic Social Finance. Meanwhile, according to expert judgments, integrating national data (0.24) and optimizing technology use (0.18) is a priority solution and strategy.  Practical implication – The findings emphasize the critical importance of improving the quality of technology-based human resources, with experts identifying national data integration as the most critical solution. As a result, relevant stakeholders are concerned about technology management training for Islamic philanthropic managers, with the goal of maximizing the potential of technology's growing role and adoption.  Originality/value – This study develops solutions and strategies for short and long-term Islamic social finance integration by identifying the benefit, opportunity, cost, and risk of six related aspects. As a result, the solutions and strategies developed will be more integrative and sustainable for the betterment of Islamic Social Finance in the near future. |
| **Order of Authors:** | Anidah Robani |
|  | Tika Widiastuti |
|  | Puji Sucia Sukmaningrum |
|  | Imron Mawardi |
|  | Sri Ningsih |
|  | Sri Herianingrum |
|  | Muhammad Ubaidillah Al-Mustofa |
| **Additional Information:** |  |
| **Question** | **Response** |

*Powered by Editorial Manager® and ProduXion Manager® from Aries Systems Corporation*

| **Financial Disclosure**  Enter a financial disclosure statement that describes the sources of funding for the work included in this submission. Review the [submission guidelines](http://journals.plos.org/plosone/s/submission-guidelines#loc-financial-disclosure-statement) for detailed requirements. View published research articles from [*PLOS ONE*](http://journals.plos.org/plosone/) for specific examples.  This statement is required for submission and **will appear in the published article** if the submission is accepted. Please make sure it is accurate. | This research grant is a Covid-19 Research Mandate with the contract number 1025/UN3.15/PT/2021. The authors would like to thank the Research and Community Service Institution (LPPM) of Universitas Airlangga for their financial assistance. The authors would also like to thank all academicians, regulators, associations, practitioners, and community leaders for their support in writing this research. |
| --- | --- |
| **Unfunded studies**  Enter: *The author(s) received no specific funding for this work.*  **Funded studies**  Enter a statement with the following details:   - Initials of the authors who received each award - Grant numbers awarded to each author - The full name of each funder - URL of each funder website - Did the sponsors or funders play any role in the study design, data collection and analysis, decision to publish, or preparation of the manuscript? - **NO** - Include this sentence at the end of your statement: *The funders had no role in study design, data collection and analysis, decision to publish, or preparation of the manuscript.* - **YES** - Specify the role(s) played. |  |
| * typeset |  |
| **Competing Interests**  Use the instructions below to enter a competing interest statement for this submission. On behalf of all authors, disclose any [competing interests](http://journals.plos.org/plosone/s/competing-interests) that could be perceived to bias this work—acknowledging all financial support and any other relevant financial or non- financial competing interests.  This statement is required for submission | No authors have competing interests |

*Powered by Editorial Manager® and ProduXion Manager® from Aries Systems Corporation*

| and **will appear in the published article** if the submission is accepted. Please make sure it is accurate and that any funding sources listed in your Funding Information later in the submission form are also declared in your Financial Disclosure statement.  View published research articles from  [*PLOS ONE*](http://journals.plos.org/plosone/) for specific examples. |  |
| --- | --- |
| **NO authors have competing interests**  Enter: *The authors have declared that no competing interests exist.*  **Authors with competing interests**  Enter competing interest details beginning with this statement:  *I have read the journal's policy and the authors of this manuscript have the following competing interests: [insert competing interests here]* |  |
| * typeset |  |
| **Ethics Statement**  Enter an ethics statement for this submission. This statement is required if the study involved:   - Human participants - Human specimens or tissue - Vertebrate animals or cephalopods - Vertebrate embryos or tissues - Field research   Write "N/A" if the submission does not require an ethics statement.  General guidance is provided below. Consult the [submission guidelines](http://journals.plos.org/plosone/s/submission-guidelines#loc-guidelines-for-specific-study-types) for detailed instructions. **Make sure that all information entered here is included in the Methods section of the manuscript.** | Informed consent for participation was obtained from respondents who participated in the survey. The respondents who participated in the survey were asked to read the ethical statement (there is no compensation for responding nor is there any known risk. To ensure that all information will remain confidential, please do not include your name. Participation is strictly voluntary and you may refuse to participate at any time) and proceed only if they agree. No data was collected from anyone under 16 years old.  Human Subject Research which involves human participants. Research and Community Service Institution (LPPM) of Universitas Airlangga for their ethical consent and approval for this research. They could be contacted at [penelitian@feb.unair.ac.id](mailto:penelitian@feb.unair.ac.id) |

*Powered by Editorial Manager® and ProduXion Manager® from Aries Systems Corporation*

| **Format for specific study types**  **Human Subject Research (involving human participants and/or tissue)**   - Give the name of the institutional review board or ethics committee that approved the study - Include the approval number and/or a statement indicating approval of this research - Indicate the form of consent obtained (written/oral) or the reason that consent was not obtained (e.g. the data were analyzed anonymously)   **Animal Research (involving vertebrate animals, embryos or tissues)**   - Provide the name of the Institutional Animal Care and Use Committee (IACUC) or other relevant ethics board that reviewed the study protocol, and indicate whether they approved this research or granted a formal waiver of ethical approval - Include an approval number if one was obtained - If the study involved *non-human primates*, add *additional details* about animal welfare and steps taken to ameliorate suffering - If anesthesia, euthanasia, or any kind of animal sacrifice is part of the study, include briefly which substances and/or methods were applied   **Field Research**  Include the following details if this study involves the collection of plant, animal, or other materials from a natural setting:   - Field permit number - Name of the institution or relevant body that granted permission |  |
| --- | --- |
|  |  |
| **Data Availability**  Authors are required to make all data underlying the findings described fully available, without restriction, and from the time of publication. PLOS allows rare exceptions to address legal and ethical concerns. See the [PLOS Data Policy](http://journals.plos.org/plosone/s/data-availability) and [FAQ](http://journals.plos.org/plosone/s/data-availability#loc-faqs-for-data-policy) for detailed information. | No - some restrictions will apply |

*Powered by Editorial Manager® and ProduXion Manager® from Aries Systems Corporation*

| A Data Availability Statement describing where the data can be found is required at submission. Your answers to this question constitute the Data Availability Statement and **will be published in the article**, if accepted. |  |
| --- | --- |
| **Important:** Stating ‘data available on request from the author’ is not sufficient. If your data are only available upon request, select ‘No’ for the first question and explain your exceptional situation in the text box. |  |
| Do the authors confirm that all data underlying the findings described in their manuscript are fully available without restriction? |  |
| **Describe where the data may be found in full sentences. If you are copying our sample text, replace any instances of XXX with the appropriate details.**   - If the data are **held or will be held in a public repository**, include URLs, accession numbers or DOIs. If this information will only be available after acceptance, indicate this by ticking the box below. For example: *All XXX files are available from the XXX database (accession number(s) XXX, XXX.*). - If the data are all contained **within the manuscript and/or Supporting Information files**, enter the following: *All relevant data are within the manuscript and its Supporting Information files.* - If neither of these applies but you are able to provide **details of access elsewhere**, with or without limitations, please do so. For example:   *Data cannot be shared publicly because of [XXX]. Data are available from the XXX Institutional Data Access / Ethics Committee (contact via XXX) for researchers who meet the criteria for access to confidential data.*  *The data underlying the results presented in the study are available from (include the name of the third party* | Data cannot be shared publicly because of data integrity and security. Data are available from the Universitas Airlangga Institutional Data Access / Ethics Committee (contact via email tika.widiastuti@feb.unair.ac.id) for researchers who meet the criteria for access to confidential data. |

*Powered by Editorial Manager® and ProduXion Manager® from Aries Systems Corporation*

| *and contact information or URL).*   - This text is appropriate if the data are owned by a third party and authors do not have permission to share the data.   * typeset |  |
| --- | --- |
| Additional data availability information: | Tick here if your circumstances are not covered by the questions above and you need the journal’s help to make your data available. |

*Powered by Editorial Manager® and ProduXion Manager® from Aries Systems Corporation*

Manuscript	[Click here to access/download;Manuscript;PLOS ONE_LATEST MANUSCRIPT.docx](https://www.editorialmanager.com/pone/download.aspx?id=29959281&guid=1b7694f2-e47e-415f-aff3-3e5957d3609b&scheme=1)

# Integrating Sustainable Islamic Social Finance: Analytical Network Process using Benefit Opportunity Cost Risk (ANP BOCR) Framework: The Case of Indonesia

Tika Widiastuti^1^, Anidah Robani^2,^ Puji Sucia Sukmaningrum^3^, Imron Mawardi^4^, Sri Ningsih^5^, Sri Herianingrum^6^, Muhammad Ubaidillah Al-Mustofa^7^

^1,3,4,6,7^Department of Islamic Economic, Faculty of Economic and Business, Universitas Airlangga

^5^Department of Accounting, Faculty of Economic and Business, Universitas Airlangga

^2^Institute of Technology Management and Entrepreneurship, Universiti Teknikal Malaysia Melaka (UTeM)

Email: ^1*^[tika.widiastuti@feb.unair.ac.id,](mailto:tika.widiastuti@feb.unair.ac.id) [^2^anidah@utem.edu.my,](mailto:2anidah@utem.edu.my) [^3^puji.sucia@feb.unair.ac.id,](mailto:3puji.sucia@feb.unair.ac.id) [^3^ronmawardi@feb.unair.ac.id,](mailto:3ronmawardi@feb.unair.ac.id) [^5^sri.ningsih@feb.unair.ac.id,](mailto:5sri.ningsih@feb.unair.ac.id) [^6^sri.herianingrum@feb.unair.ac.id,](mailto:6sri.herianingrum@feb.unair.ac.id) [^7^ubaid9339902@gmail.com](mailto:7ubaid9339902@gmail.com)

# Abstract

**Purpose –** This research analyzes solutions and priority strategies for integrating sustainable Islamic Social Finance that could be implemented in the short and long term by analyzing it through the Benefit, Opportunity, Cost, and Risk (BOCR) framework, which comprises of six aspects: Governance, Sustainable Financing, philanthropic Institutions, Human Resources, Regulations, and Technology.

***User***

*2021-12-07 16:28:33*

--------------------------------------------

Before the objective chapter, provide background on the problem related to the topic discussed because the goal is to answer the existing problems. It is necessary to briefly explain what is meant by a philanthropic institution and how does it relate to the topic of discussion?

**Design/methodology/approach –** This qualitative research employs the Analytic Network Process method using Benefit, Opportunity, Cost, and Risk analysis. Data obtained mainly through focus group discussions and in-depth interviews with experts from academics, practitioners, associations, regulators,

***User***

*2021-12-05 13:57:27*

--------------------------------------------

explain how to determine the sample used by the author

and community leaders. The data is processed with Microsoft excel and Super Decision software.

**Findings –** There are several findings worth considering. First, the highest priority integrating Islamic social finance aspects are human resources (0.97), regulation (0.86), and technology (0.76). Second, based on a short and long-term analysis, financial integration through sustainable financing (0.01 and 1.44) and improving human resource quality through certification and training (0.01 and 1.56) is a priority solution and strategy to integrate sustainable Islamic Social Finance. Meanwhile, according to expert judgments, integrating national data (0.24) and optimizing technology use (0.18) is a priority solution and strategy.

**Practical implication –** The findings emphasize the critical importance of improving the quality of technology-based human resources, with experts identifying national data integration as the most critical solution. As a result, relevant stakeholders are concerned about technology management training for Islamic philanthropic managers, with the goal of maximizing the potential of technology's growing role and adoption.

**Originality/value –** This study develops solutions and strategies for short and long-term Islamic social finance integration by identifying the benefit, opportunity, cost, and risk of six related aspects. As a result, the solutions and strategies developed will be more integrative and sustainable for the betterment of Islamic Social Finance in the near future.

***User***

*2021-12-07 16:29:44*

--------------------------------------------

Add to keyword: priority strategy

**Keywords:** Financial Sustainability, Integration, Islamic Social Finance, ANP BOCR

**Paper Type** Research paper

# Introduction

Islamic social finance (ISF), which includes zakat, *infaq*, *sadaqah*, waqf, and Islamic microfinance, is critical to promoting long-term economic development (Ibrahim & Ghazali, 2014; Zauro et al., 2020; Widiastuti et al., 2021). Islamic social finance has also shown great

***User***

*2021-12-07 16:30:27*

--------------------------------------------

First, state the role of Islamic finance in global development and humanity, both in Muslim and non-Muslim countries when there are no pandemic issue. The illustration can be expanded not only to the problem of the Covid-19 Pandemic (monetary crisis or global economic crisis, global food and financial crisis)

promise in addressing socioeconomic issues, such as the Covid-19 pandemic. The Covid-19 pandemic has had a significant impact, especially in the economic field. The economic implications of Covid-19, particularly in Indonesia (Caraka et al., 2020; Suryahadi et al., 2020; World Bank, 2020), include increasing unemployment and underemployment rates, declining income levels, economic contraction, slow and uneven recovery process across sectors, including the health, economic and education sectors. This is due to the limited sources of funding owned by the state. The government has prepared a reserve budget of USD 5.3 billion and a budget for the National Economic Recovery program of USD 37.3 billion. The funding is used for the health sector (USD 1.0 billion), social protection (USD 2.5 billion), and support for Micro, Small, and Medium Enterprises (USD 1.8 billion) (Ministry of Finance, 2020). Sources of funds are derived from debt, government bonds, Sukuk, and foreign debt. The Ministry of Finance (2020) released funding components of Covid-19 originating from Market Financing, Bilateral and Multilateral Debt, Private placement, Financing from Over Budget Balance (SAL), and the use of Government Endowment Funds. These components certainly have limits to meet the growing needs of handling Covid-19. Consequently, the government needs to find alternative sources of funding. Islamic social finance with great potential can be utilized to lessen the burden of funding and accelerate the recovery from the impact of the Covid-19 pandemic.

Indonesia, as a Muslim majority country, has a high potential for Islamic Social Finance. The

***User***

*2021-12-07 16:31:33*

--------------------------------------------

What are other contributing factors besides governance, complete with other relevant literature reviews (LR), such as aspects of fundraising/sharia finance, management, transparency, mutual trust, etc.

potential for collecting zakat funds in Indonesia in 2020 is USD 23 billion (BAZNAS, 2021),

cash waqf is USD 12.6 billion per year, and the overall value of waqf land is USD 140 billion

(Ministry of Finance, 2021). However, the collection of zakat and waqf funds is still far from

its full potential. The zakat collection in Indonesia in 2019 was only USD 0.7 billion

(BAZNAS, 2021), while the cash waqf funds collected were only USD 0.2 billion (Ministry of

Finance, 2021). One of the reasons for the vast gap between the potential and potential of

Islamic social finance is a lack of optimal governance (Widiastuti et al., 2021).

***User***

*2021-12-05 15:03:17*

--------------------------------------------

First, explain what ISF governance is, then explain its aspects.

The issues of ISF governance can be categorized into six aspects consisting of Human Resources (HR), Regulation, Technology, Sustainable financing, Governance, and Institutions (Beik, 2015; Pitchay et al., 2018; Ahmad, 2019; Ali et al., 2020; Mustofa et al., 2020; Sukmana, 2020; Widiastuti et al., 2021). Problems in the HR aspect include the lack of ability and motivation of human resources to develop, both human resource managers and recipients of Islamic social funds (Widiastuti et al., 2021). In addition, human resources in Islamic philanthropic institutions are dominated by employees not from the background of Islamic Economics and ZISWAF management, causing the low ability of HR to manage ZISWAF. In addition, the low literacy related to Islamic social funds needs to be addressed by policymakers (Ali et al., 2020).

***User***

*2021-12-07 16:34:20*

--------------------------------------------

Please cite more fully and in detail regarding policies and regulations that do not support it, as well as what policy solutions can be offered?

Unsupported policies and regulations become issues from the regulatory aspect (Mustofa et al., 2020; Widiastuti et al., 2021). In Indonesia, zakat regulation is still voluntary. Furthermore, zakat and waqf are governed by different laws; zakat is governed by Law No. 23 of 2011,

whereas waqf is governed by Law No. 41 of 2004. Furthermore, the infrastructure required for management is incomplete, particularly in the field of technology. This becomes an issue that deserves consideration (IRTI, 2014). Many institutions do not have the financial capacity to purchase the infrastructure needed for technology-based governance.

Problems related to the sustainability aspects of the ISF program are of concern to IRTI (2014), Pitchay et al. (2018), and Sukmana (2020). Most empowerment programs using Islamic social

***User***

*2021-12-05 15:26:03*

--------------------------------------------

provide a clear source as a reference

funds do not consider sustainability aspects and seem to end when the program ends, so that

the primary goal of empowerment, namely the transformation of *mustahiq* into *muzakki*, or

*mauquf 'alaihi* into *wakif*, cannot be achieved. In addition, in the aspect of governance, Islamic

social fund instruments are still partially managed. These instruments have not been integrated

***User***

*2021-12-05 15:38:48*

--------------------------------------------

Add the challenge from the side of Muslims who oppose the ISF being regulated by the government because Indonesia is based on Pancasila, not a country with Islamic law, so its population consists of various beliefs and religions. There could be a sense of injustice if the government only regulates and takes money from Muslims to help government finances.

with the State's fiscal policy (Beik, 2015). On the institutional aspect, there is a lack of

coordination and synergy between stakeholders (Widiastuti et al., 2021). The lack of public

trust in Islamic social finance managers (Ahmad, 2019) needs to be an essential concern for

policymakers. To overcome these problems, the solution that can be done is to formulate the

integration of the Islamic social finance sector and establish a sustainable management

ecosystem.

The purpose of this research is to develop a strategy for the short and long-term integration of Islamic social finance and to determine the best way to achieve that goal. This study was carried out in Indonesia, a country with a high potential for accumulating Islamic social funds. In practice, however, the Islamic social funds are only partially managed and have not been fully integrated. Raimi et al. (2014), Haneef et al. (2015), Jouti (2019), and Shalleh et al. (2020) conducted research on ISF integration. Previous research formulated the integration of Islamic social funds without emphasizing sustainability; thus, the sustainability aspect in integrating Islamic social funds was highlighted in this research. Furthermore, previous studies employed a qualitative approach through case studies, literature reviews, content and thematic analysis, and a quantitative approach through Structural Equation Modeling (SEM). There has been no research that incorporates Islamic Social Funds that employ the two-level ANP method. The analysis of Benefit, Opportunity, Cost, and Risk (BOCR) to build the ANP framework is what makes this research unique. Several factors influenced the decision to use this method. To begin, the ANP method is used to determine the best integration in the long-term management of Islamic social finance. Second, analyze the benefits, opportunities, costs, and risks of each aspect to produce findings that can be implemented in the short and long term. Third, this study identifies priority solutions and strategies for achieving long-term ISF integration using the ANP BOCR method.

The findings show that the financing integration model is a priority solution in the short and long term. This demonstrates ISF serve several functions: consumptive zakat is used to provide emergency assistance in the short term, productive zakat and benevolence funds are used to support economic recovery in the medium term, and waqf or sukuk are used to build long-term resilience. Furthermore, the findings of this study highlight the importance of improving the quality of HR managers through technology-based certification and training (particularly for

data analysis and big database management) as the best short and long-term strategy for implementing Islamic social finance in a sustainable manner.

The findings of this study are critical for several reasons. First, solutions and strategies for the integration of sustainable ISF are expected to solve various problems of managing different instruments of ISF, resulting in the formation of a sustainable Islamic social finance ecosystem. Second, the findings of this study will serve as a resource for ISF institutions in developing long-term strategic plans. Third, the results can provide a broader impact on people's well- being. Fourth, in the context of the Covid-19 pandemic, the findings can provide an overview of strategic policy steps and guidelines for seeking alternative funding sources by leveraging the potential of sustainable Islamic social funds.

# LITERATURE REVIEW

2.1 **The concept of Islamic Social Finance and its instruments**

***User***

*2021-12-07 17:03:11*

--------------------------------------------

Literature Review for contemporary Islamic finance needs to be enriched especially from reputable global indexed primary journals, covering Islamic banking, Islamic insurance, etc.

Islamic Social Finance is a financial system that includes traditional and contemporary Islamic social finance instruments (IRTI, 2020). Traditional Islamic Social Finance can be divided into two types: philanthropy and cooperation. Zakat, *infaq*, sadaqah, and waqf are traditional Islamic social finance instruments based on philanthropy. Meanwhile, the traditional Islamic social finance based on collaboration includes *qard* and *kafalah*. Furthermore, Islamic microfinance can be categorized as contemporary Islamic social finance. Islamic Social Finance contributes to the third sector of the economy by assisting in reducing unemployment and poverty (Hamed, 2020). Several previous studies have revealed differences in the instruments used in Islamic Social Finance, as shown in Table 1:

# Table 1. Instruments of Islamic Social Finance

| No | Research | Year | Zakat | Infaq | Sadaqah | Waqf | Qard | Qard Hasan | Kafalah | Gift | Islamic Microfinance |
| --- | --- | --- | --- | --- | --- | --- | --- | --- | --- | --- | --- |
| 1 | Sulistyowati | 2018 | ✅ | ✅ |  | ✅ |  |  |  |  |  |
| 2 | Jouti | 2019 | ✅ |  | ✅ | ✅ | ✅ |  | ✅ |  | ✅ |
| 3 | Usman et al. | 2019 | ✅ | ✅ | ✅ |  |  |  |  |  |  |
| 4 | Kamaruddin and  Auzair | 2019 | ✅ |  | ✅ | ✅ |  | ✅ |  | ✅ |  |
| 5 | Adjar et al. | 2020 | ✅ |  |  | ✅ |  | ✅ |  |  |  |
| 6 | Arumsari and  Rikawati | 2020 | ✅ | ✅ | ✅ | ✅ |  |  |  |  |  |
| 7 | Hudaefi | 2020 |  | ✅ | ✅ | ✅ |  |  |  |  |  |

Source: Authors’ Compilation (2021)

Most of the previous research mentions that Islamic Social Finance instruments include Zakat, *Infaq*, *Sadaqah*, and Waqf (Sulistyowati, 2018; Jouti, 2019; Kamaruddin & Auzair, 2019; Adjar et al., 2020; Arumsari & Rikawati, 2020; Hudaefi, 2020). Therefore, in this study, what is meant by Islamic Social Finance instruments are Zakat, *Infaq*, Alms and Waqf, and Islamic microfinance.

Each of these instruments has different roles and functions but shares the same objective: achieving prosperity (Jouti, 2019). The first instrument is Zakat, a mandatory economic instrument for every Muslim (Kasri, 2017; Kailani & Slama, 2020). Zakat serves as a means for distributing wealth in society (Bilo & Machado, 2020) from the rich to the poor to achieve socio-economic justice, increase economic growth and reduce inequality (Manurung, 2013). The second instrument is *infaq* and *sadaqah*, voluntary gifts by capable people to seek the pleasure of Allah, significantly to help the needy (Azha et al., 2013). Zakat, *infaq*, and *sadaqah* are economic instruments that support economic growth by increasing the purchasing power of the needy (Raimi et al., 2014; Jermsittiparsert & Sommanawat, 2019; Widiastuti et al., 2021).

As a third instrument, Waqf is a social fund that plays a vital role in the Islamic framework. An asset owner donates and dedicates movable or immovable assets for the benefit of the community in perpetuity or impermanence. Waqf can be used to provide a variety of free community services, such as the construction of mosques, hospitals and healthcare centers, *madrasas*, education centers, and libraries (Cizakca, 1998). Waqf is a vital tool in the Islamic economic system used to meet the needs of the poorest communities and promote community economic growth (Khairi et al., 2014).

Islamic microfinance is the fourth instrument of Islamic social finance. Islamic microfinance serves two purposes: social and commercial. As a social institution, it manages zakat, *infaq, sadaqah*, and waqf in accordance with the *Syariah*. While as a commercial institution, it conducts businesses and provide financing for economic development. Islamic microfinance focuses on economic development through capital assistance and the empowerment of the marginal community with no access to capital to improve their well-being (Haneef et al., 2013).

# 2.2  Sustainability of Islamic Social Finance

Sustainability refers to the capacity to operate over a long period and continuously (Pischke, 1996). There are two paradigms in sustainability, namely the institutionalist and welfarist (Brau et al., 2004). The institutionalist paradigm considers that financial self-sufficiency is the key to achieving sustainability. Thus, a person can be said to be sustainable if he or she achieve financial independence (Brau et al., 2004). In contrast, the welfarist paradigm considers that social functions in poverty alleviation are more important in achieving sustainability. Both paradigms are essential in the context of Islamic Social Finance. Financial self-sufficiency refers to an institution's ability to fund its operations independently while also benefiting others. On the other hand, the main goals of Islamic Social Finance are poverty alleviation and its reachability (Ali et al., 2015; Ahmed et al., 2017; Rosli et al., 2018; Bin-Nashwan et al., 2020). Three factors contribute to Islamic Social Finance sustainability. First, the collection's sustainability is linked to the fact that funds will always be collected from the community. To ensure the long-term viability of supply funds, all information about zakat and its administration must be transparent and easily accessible to the public (Taha et al., 2017). Second, institutional sustainability is linked to the institution's ability and availability to carry out operational tasks (Chowdhury et al., 2011). Third, the sustainability of distribution is linked

***User***

*2021-12-05 16:10:23*

--------------------------------------------

Provide an explanation of the current state of sustainability of ISF along with references from previous research. If there is no review on this matter, please provide a reference that strengthens your statement that most of the empowerment programs sourced from the ISF do not meet the sustainability aspect.

to how the Islamic Social Finance Institution performs its social and intermediary functions to improve welfare and achieve broader benefits (Ahmed et al., 2017; Bassem et al., 2019).

# 2.3 Issues with Islamic Social Finance Integration

In order to achieve optimal management, Islamic Social Finance must overcome several challenges. A lack of motivation among beneficiaries to develop, a lack of synergy and coordination among stakeholders, a lack of human resources capabilities and quantity to manages Islamic social finance, a lack of ability to optimize technology, regulations that are still voluntary, and a lack of awareness and trust of muzakki in Islamic Social Finance institutions are among the problems encountered by Islamic Social Finance (IRTI, 2014; Pitchay et al., 2018; Ahmad, 2019; Ali et al., 2020; Mustofa et al., 2020; Sukmana, 2020; Widiastuti et al., 2021). Further, the Islamic social finance instruments are still partially

***User***

*2021-12-07 16:38:29*

--------------------------------------------

Is this sentence incomplete or imperfect? Correct this sentence so that it is easier to understand

managed. The following points demonstrate that Islamic social finance instruments are still partially managed. First, according to the 2017-2022 Indonesian Zakat Architecture, zakat management is supported by regulations but remains a voluntary instrument. Second, Islamic social fund instruments are governed by different regulations and laws. Third, because Islamic social fund instruments are managed by various institutions (government, corporate and social community), there exist differences in the rights, obligations, and interests of different institutions. Fourth, the regulators of zakat and waqf are not the same. The National *Amil* Zakat Agency (BAZNAS) regulates zakat, while the Indonesian Waqf Board (BWI) is the waqf management regulator. However, BAZNAS and BWI were eventually accountable to the Minister of Religion Affairs.

Given the challenges which consequently create a significant gap between the potential and

***User***

*2021-12-07 16:39:30*

--------------------------------------------

In Scientific Writing, avoid in one paragraph consisting of only one sentence

actualization of Islamic social finance management and accounting for the enormous potential

of Islamic social finance, it is necessary to integrate Islamic Social Finance instruments on an

ongoing basis to achieve optimum level of management.

# 2.4 Integration Model of Islamic Social Finance

***User***

*2021-12-07 16:40:42*

--------------------------------------------

The importance of including Literature Review about integration aspects of growth, aspects of justice and aspects of sustainability

Integration refers to the process of blending into a single unit, thus, integration in Islamic social finance combines funds management, institutions, governance, functions, etc. Raimi et al. (2014) incorporated Corporate Social Responsibility, Zakat, and Waqf into the Faith-Based Model to reduce poverty, promote business development, and strengthen the economy of Muslim-majority communities. Hassanain (2015) conducted a literature review on microfinance, waqf, and zakat to develop an integration model. Sulistyowati (2018) employs qualitative methods to construct the integration of zakat and waqf for disaster management. Haneef et al. (2015) use Structural Equation Model (SEM) analysis to examine the significant influence between differencct aspect of Islamic social finance that include waqf, takaful, human resource management, financing, etc. Pitchay et al. (2018) conducted a literature review to develop a cooperative-waqf model. Further, using thematic analysis, Ambrose et al. (2018) developed a waqf financing model for public goods and mixed public goods in Malaysia, where the proceeds of waqf investment can be used to fund 11 items of government spending.

Research that specifically discusses the sustainability of the Islamic Social Finance model was carried out by Jouti (2019), which focused on creating an Islamic Social Finance ecosystem,

and Shalleh et al. (2020), which focuses on the integration of waqf-based takaful for flood victims in Malaysia. This study develops previous research by building an integration model between Islamic Social Finance instruments with an emphasis on sustainability aspects using the two-level ANP method.

# METHOD

3.1 **Research Method and Analysis Approach**

***User***

*2021-12-07 16:41:19*

--------------------------------------------

Mention what criteria are meant?

The Analytical Network Process (ANP) method is used in this qualitative study, along with the analysis of Benefit, Opportunity, Cost, and Risk (BOCR). This two levels ANP method has the characteristics of mathematically examined conditions and inputs obtained based on several

criteria, allowing it to aid decision-making from various options and complex data (Saaty, 2013). The ANP method approach was chosen because Islamic social funds include various instruments, each with its own set of characteristics and issues, both tangible and intangible. In this study, the ANP approach is the best choice for producing strategic decisions. Meanwhile, the BOCR analysis was selected for a variety of reasons. To begin, every decision has both positive and negative consequences, which must be considered when determining priority decisions (Saaty & Vargas, 2006). Second, the BOCR approach is used to make decisions that can be implemented in the short and long terms. The ANP BOCR method prioritizes solutions and strategies to develop a sustainable Islamic social finance integration model. The ANP method of research has three phases, which are depicted in Figure 1 below (Ascarya, 2005):

***User***

*2021-12-07 16:41:41*

--------------------------------------------

Briefly state what reasons are meant?

# Figure 1. ANP BOCR Research Stage

Source: (Ascarya, 2005)

First, the decomposition phase seeks to construct and validate the ANP model through Focus Group Discussions and in-depth interviews with experts in Islamic social finance from academics, practitioners, associations, regulators, and community leaders. In addition, a

***User***

*2021-12-07 16:42:45*

--------------------------------------------

Mention the Literature Review focused on what aspects?

literature review was conducted as part of this study to strengthen the ANP model that was

developed. Three focus group discussions were held. The first and second focus group discussions (FGDs) were held with experts to identify aspects, solutions, and strategies for integrating sustainable Islamic social finance and identify benefits, opportunities, costs, and risks to determine the short-term and long-term analysis. The model developed through FGD 1 and 2 was then strengthened through in-depth interviews and literature reviews. Following that, the third FGD validates the constructed ANP model. Second, the pairwise comparison phase to design a questionnaire in the form of a pairwise comparison to determine and analyze aspects, solutions, and priority strategies in integrating sustainable Islamic social finance. The questionnaire that the respondents have answered will be used for the next phase. The third is the data analysis phase which aims to calculate the priority value of each aspect, solution, and strategy formulated using Microsoft Excel and Super Decision software. The use of the ANP method with the BOCR approach makes it possible to produce realistic and net values. The net value/additive negative describes alternative options that are more profitable or ideal to be prioritized in the long term, while realistic/multiplicative value represents more favorable outcomes for short-term decisions (Saaty & Vargas, 2006; Zammori et al., 2014). According

to (Saaty & Vargas, 2006), there are several steps to synthesize each priority option from each criterion where:

Net Value/additive negative = bB+oO-cC-rR

Realistic/multiplicative = (BxO)/(CxR)

(Equation 1)

(Equation 2)

B or b stands for Benefit, O or o stand for Opportunity, C or c stands for cost, R or r stands for Risk. After the analysis phase, the next step is to validate the results with various Islamic Social Finance experts to strengthen the integration model that will be built.

# 3.2 Data and Research Samples

The data used are primary data obtained through focus groups, in-depth interviews, and ANP questionnaires filled out by respondents. The data sources gathered from 13 experts from academics, practitioners, associations, regulators, and community leaders. In ANP research, the number of respondents is not the most important requirement for research validation (Firmansyah and Sukmana, 2014), but rather their expertise. Each expert completed the questionnaire consciously and in a mentally healthy state. The participants are required to fill in a specific consent form beforehand to ensure that they are not subject to any pressure from any party to participate in the interviews. The research team guarantees the confidentiality of the experts' data and answers and is fully responsible for all applicable laws. Applicable laws will account for the disclosure of confidential data and expert responses.

In terms of research ethics, this research follows the rules and ethics of research conduct and writing coordinated by the Institute for Research and Community Service (LPPM), Universitas Airlangga and those mandated by law. It is also explained in the Airlangga University Chancellor's Regulation Number 34 of 2019 concerning the Rules of Conduct clause 16 (b) that researchers are honest, objective, and adhere to all aspects of the research process and may not falsify or manipulate data or research results, as well as clause 16 (f) that researchers must respect and appreciate the object of research, whether in the form of humans or animals, both living and dead, or parts/fragments of the object of research. In addition, this research is supervised by the Center for Research and Publications (3P) within the Faculty of Economics and Business Universitas Airlangga, which functions as an ethics committee according to Dean Decree Number 88/UN3.1.4/2020.

The purposive sampling technique aims to select respondents according to the criteria required in the ANP method. For this study, the selected respondents must have the following criteria:

(1) respondents are experts in the field of Islamic social finance from academics, practitioners, associations, regulators, and community leaders; (2) have more than two journal publications in the field of Islamic social finance for academic representatives; and (3) for representatives of regulator, practitioners, and associations, should hold an important position and bear great responsibility in the management of Islamic social finance in their respective institutions. The

research team will contact prospective (representative) respondents by phone and email before conducting interviews to inquire about their willingness to complete the questionnaire. There are two options for completing the questionnaire: online or offline. In the case where the respondent opted to complete the questionnaire offline, the research team will attended to them, while adhering to a strict health protocol, by still maintaining a safe distance and wearing a mask. For respondents who opted to fill out questionnaires via teleconference zoom, accompanied by three team members with each job desk: a moderator, a note-taker, and a backup. The list of respondents in this study is shown in Table 2:

# Table 2. List of Research Respondents

| No | Representative | Name | Institution | | | | | Affiliation | | |
| --- | --- | --- | --- | --- | --- | --- | --- | --- | --- | --- |
| 1 | Association | KH | National Zakat Forum in East Java | | | | | Leader | | |
| 2 |  | BS | National Zakat Forum | | | | | Leader | | |
| 3 | Practitioner | CW | Zakat Institution “*Manajemen Infak*” | | | | | Director of mobilization, research,  and transformation of Zakat Institution | | |
| 4 |  | MHZ | BAZNAS Centre Strategic of Studies | | | | | Director | | |
| 5 | Regulator | SPY | Ministry of Religious Affairs | | | | | Staff at Ministry of Religious Affairs | | |
| 6 |  | SEH | National Committee on Islamic Economy and Finance (KNEKS) | | | | | Director of Infrastructure | Sharia | Ecosystem |
| 7 |  | AJ | National Committee on Islamic Economy and  Finance (KNEKS) | | | | | Director of Sharia Social Finance | | |
| 8 | Academics | NH | Universitas Yarsi | | | | | Professor in Islamic Economics | | |
| 9 |  | RS | Universitas Airlangga | | | | | Professor in Islamic Economics | | |
| 10 |  | MSN | Universitas Indonesia | | | | | Head of Sharia Finance Education and Human Resources Development  Division | | |
| 11 | Community Leader | MTH | Department  Registration | of | Population | and | Civil | Secretary | | |
| 12 |  | MZK | Al Mustofa  Foundation |  | Educational | and | Social | Founder | | |
| 13 |  | NS | Indonesian Zakat Initiative (IZI) | | | | | Program Director | | |

Source: Author’s compilation (2021)

This study's questionnaire is in the form of a pairwise comparison of the benefits, opportunities, costs, and risks of related aspects in integrating sustainable Islamic social finance. Governance, sustainable financing, institutions, regulations, technology, and human resources are among the issues at stake in integrating the instruments of Islamic social finance. The questionnaire also develops various solutions and strategies to achieve the research objectives. The pairwise comparison assessment in the questionnaire is rated on a scale of 1-9, where one means not important/needed/influential and nine means absolutely important/needed/influential.

# 4. Result

4.1 **Priority Aspect**

Human resources, regulation, technology, sustainable financing, governance, and institutions are six aspects related to the integration of sustainable Islamic social finance, according to the

FGD and in-depth interviews. Table 3 shows the aspects with the highest priority based on ANP data processing.

# Table 3. ANP BOCR Result of Priority Aspect

| Aspect | Code | Benefit | Opportunity | Cost | Risk | Realistic | Rank |
| --- | --- | --- | --- | --- | --- | --- | --- |
| Human Resources at Islamic Social Finance  Institution | A | 0.1029 | 0.19763 | 0.16966 | 0.12334 | 0.97181 | 1 |
| Regulation | B | 0.15479 | 0.09765 | 0.11073 | 0.1571 | 0.8689 | 2 |
| Technology Use | C | 0.24925 | 0.16373 | 0.24824 | 0.214 | 0.7682 | 3 |
| Sustainable Financing | D | 0.1229 | 0.06846 | 0.10973 | 0.10029 | 0.7645 | 4 |
| Governance | E | 0.23496 | 0.33413 | 0.17927 | 0.25364 | 0.7152 | 5 |
| Institution | F | 0.13521 | 0.13841 | 0.18238 | 0.15163 | 0.6767 | 6 |

Source: Primary Data Prosessed (2021)

***User***

*2021-12-07 16:43:48*

--------------------------------------------

What is the important role in this case? Give a brief explanation

The data processing results show that human resource is the highest priority aspect in Islamic social finance institutions, with a value of 0.97181. Human resources play a crucial role in

Islamic Social Finance management. With a value of 0.8689, the regulation comes in the second priority. Regulations are all rules put in place to provide legal guidance and support for the management and development of Islamic Social Finance. With a value of 0.7682, the third priority aspect is the use of technology, while sustainable financing comes in the following priority order with a value of 0.7645. Sustainable funding is associated with business development and achieving financial independence using Islamic social funds. Governance is a fifth priority aspect with a value of 0.7152. The collection, distribution, and utilization process in Islamic social finance are governed by transparency, accountability, responsibility, independence, and fairness. The institutional aspect comes as the least priority, with a score of 0.6767.

***User***

*2021-12-07 16:45:09*

--------------------------------------------

What institutional aspects are the focus of this article (structure, function/role, coordination system, business partnerships with other parties).

# 4.2 Priority Solution in integrating sustainable Islamic social finance

There are eight solutions to integrate sustainable Islamic social finance based on the findings of the focus group, in-depth interviews, and literature review. The first is (SL.1) financial integration in providing funding to beneficiaries through the distribution of zakat, waqf, and Islamic microfinance based on the beneficiaries' economic circumstances and capabilities. The second step is (SL.2) integrating Islamic social finance with state fiscal instruments, which is supported by existing regulations. Integrating Islamic social philanthropy with fiscal policy can be accomplished by making zakat a tax credit or tax deduction. Third is (SL.3) integration through non-formal coordination between Islamic social finance institutions. The Islamic social finance institutions may establish joint coordinations to conduct empowerment programs while prioritizing the work specialization of each institution. Fourth is (SL.4) integration among Islamic social finance institutions based on capacity building and role division. This

***User***

*2021-12-07 16:46:07*

--------------------------------------------

What types of integration are meant (horizontal integration, vertical integration or horizontal coordination, vertical coordination)?

type of integration is dependent on the specialization of each institution and community. Some organizations concentrate on the health sector, while others concentrate on economic empowerment and education. As a result, combining institutions based on their specialization

may be the best option for achieving the research objective. Fifth is (SL.5) establishing an integrated national data center for all Islamic social finance instruments; data that must be integrated include financial statements, donor funds, beneficiary data, and other relevant information. Sixth is (SL.6) includes integration based on regional clusters with solid authority. This integration may concentrate on integrating Islamic social finance institutions into geographical groups. The seventh solution (SL.7) incorporates integrating Islamic social finance through the passage of new laws and regulations. Each instrument of Islamic social finance currently has its own set of rules and regulations. The eighth solution is (SL.8) integration based on Islamic social finance management clusters, such as zakat *Amils* joining other zakat institutions and waqf manager joining other waqf institutions.

# Table 4. ANP Result of Solution

| Cluster | Code | Benefit | Opportunity | Cost | Risk | Net Value | Net Value  Priorit y | Realistic | Reasli stic  Priorit y | Expert Judment | Expert Judgme  nt Priority |
| --- | --- | --- | --- | --- | --- | --- | --- | --- | --- | --- | --- |
| Solution | SL.1 | 0.0526 | 0.0588 | 0.0466 | 0.04593 | **0.0188** | **1** | **1.4435** | **1** | **0.2118** | **2** |
|  | SL.2 | 0.0277 | 0.0262 | 0.0174 | 0.02957 | **0.0070** | **2** | **1.4149** | **2** | 0.1016 | 4 |
|  | SL.3 | 0.0258 | 0.0235 | 0.0227 | 0.02338 | 0.0032 | 5 | **1.1452** | **3** | 0.0952 | 5 |
|  | SL.4 | 0.0297 | 0.0204 | 0.0226 | 0.023715 | 0.0038 | 4 | 1.1313 | 4 | 0.0929 | 6 |
|  | SL.5 | 0.0555 | 0.0657 | 0.0537 | 0.063225 | **0.0042** | **3** | 1.0737 | 5 | **0.2438** | **1** |
|  | SL.6 | 0.0244 | 0.0144 | 0.0260 | 0.020225 | -0.0073 | 7 | 0.6719 | 6 | 0.0787 | 7 |
|  | SL.7 | 0.0159 | 0.0289 | 0.0380 | 0.020995 | -0.0141 | 7 | 0.5777 | 7 | **0.1071** | **3** |
|  | SL.8 | 0.0180 | 0.0118 | 0.0226 | 0.022965 | -0.0157 | 8 | 0.4113 | 8 | 0.0685 | 8 |

Source: Primary Data Processed (2021)

***User***

*2021-12-07 16:47:42*

--------------------------------------------

What is the difference between short-term and long-term priority solutions?

The formulated solution is then processed using ANP BOCR to determine the priority order of solutions. Table 4 shows the order of priority solutions in the short term, long term, and based

on expert agreement while Table 5 summarizes the results of data processing as follows:

# Table 5. Summary of Priority Solution

| **Solution/Rank** | **Solution in Sustainable Islamic Social Finance Integration** | | |
| --- | --- | --- | --- |
|  | **1** | **2** | **3** |
| Expert Judgment | Integration of national data centers (SL.5) | Financial integration in providing funding to  beneficiaries (SL.1) | Integration through the establishmen of new laws  and regulations (SL.7) |
| Short Term Realistic Value | Financial integration in providing funding to  beneficiaries (SL.1) | Integration with state fiscal policy (SL.2) | Integration through non- formal coordination  between institutions (SL.3) |
| Long Term Net Value | Financial integration in  providing funding to beneficiaries (SL.1) | Integration with state fiscal policy (SL.2) | Integration of national data centers (SL.5) |

Source: Author’s compilation (2021)

According to the data processing results, the first and second priorities in the short and long-

***User***

*2021-12-07 16:48:34*

--------------------------------------------

It is necessary to conduct a more in-depth analysis and based on this analysis, it is synthesized by the author.

term are in the same order. The solutions that will be implemented first and foremost in the

short and long term are financial integration in providing funding to beneficiaries and

integration of Islamic social finance with state fiscal policy. According to expert judgment,

integrating national data centers is the highest priority solution, with financial integration in

providing funding to beneficiaries coming in second.

# 4.3 Priority Strategies in integrating sustainable Islamic social finance

There are nine strategies to integrate sustainable Islamic social finance based on the findings of the FGD, in-depth interviews, and literature review. The first strategy (ST.1) is to improve the quality of human resources through training and certification. The second strategy (ST.2) promotes the availability of real-time data through database integration and reporting. The third strategy (ST.3) is optimizing digital technology in Islamic social finance instruments' collection, management, and distribution process. The fourth is (ST.4) coordination and synergy among stakeholders (practitioners, regulators, associations, academicians, etc.) to establish shared commitment and vision. Fifth, (ST.5) encourages broadening the range of benefits by establishing service units at lower levels, such as service units at the district, village, and mosque levels, as well as strategic locations that support the broadening of the reach of the benefits of Islamic social finance instruments. Sixth, (ST.6) establish a new institution capable of integrating all instruments of Islamic social finance. In Indonesia, various institutions manage different Islamic social finance instruments. The seventh is (ST.7) realizing the government's role in supporting the integration of Islamic social finance through regulation and socialization; several previous studies have concluded that government support for integrating Islamic social finance is still inadequate. The eighth strategy (ST.8) focuses on developing the Islamic social finance institutions to have two functions, namely *Amil* and *Nazir*. Several Baitul Maal wat Tamwiil (BMT) has operated the two functions under the same body. The Ninth is (ST.9) revising and updating all laws and regulations governing the management of Islamic social finance instruments to accommodate the development of Islamic finance instruments. This development includes the management of zakat on profession, corporate zakat, temporary waqf, cryptocurrency waqf, stock waqf, etc.

***User***

*2021-12-07 16:49:07*

--------------------------------------------

What is the equivalent in Syar'i or Arabic?

# Table 6. ANP Result of Strategies

| Cluster | Code | Benefit | Opportunity | Cost | Risk | Net Value | Net Value Priority | Realistic | Reaslisti c Priority | Expert Judment | Expert Judgme nt  Priority |
| --- | --- | --- | --- | --- | --- | --- | --- | --- | --- | --- | --- |
| Strategies | ST.1 | 0.0455 | 0.0393 | 0.0377 | 0.030375 | **0.0168** | **1** | **1.5651** | **1** | **0.1546** | **3** |
|  | ST.2 | 0.0334 | 0.0437 | 0.0297 | 0.03745 | **0.0099** | **2** | **1.3113** | **2** | 0.1515 | 4 |
|  | ST.3 | 0.0500 | 0.0463 | 0.0444 | 0.048875 | **0.0030** | **3** | **1.0676** | **3** | **0.1885** | **1** |
|  | ST.4 | 0.0364 | 0.0450 | 0.0443 | 0.03603 | 0.0011 | 4 | 1.0289 | 4 | **0.1666** | **2** |
|  | ST.5 | 0.0235 | 0.0148 | 0.0181 | 0.02406 | -0.0038 | 6 | 0.8001 | 5 | 0.0752 | 6 |
|  | ST.6 | 0.0086 | 0.0087 | 0.0089 | 0.01088 | -0.0023 | 5 | 0.7807 | 6 | 0.0365 | 9 |
|  | ST.7 | 0.0173 | 0.0179 | 0.0213 | 0.019505 | -0.0055 | 7 | 0.7485 | 7 | 0.0750 | 7 |
|  | ST.8 | 0.0214 | 0.0140 | 0.0203 | 0.02178 | -0.0066 | 8 | 0.6796 | 8 | 0.0723 | 8 |
|  | ST.9 | 0.0135 | 0.0199 | 0.0250 | 0.02105 | -0.0126 | 9 | 0.5124 | 9 | 0.0795 | 5 |

Source: Data Primary Result (2021)

***User***

*2021-12-07 16:50:59*

--------------------------------------------

Give a brief description before referring to tables 6 and 7. In scientific writing, avoid one paragraph consisting of only one sentence.

The order of priority strategies is shown in table 6, which displays short-term, long-term

priority solutions and expert judgment. The strategies are summarized in table 7 as follows:

# Table 7. Summary of Priority Strategies

| **Strategies** | **Strategies in Sustainable Islamic Social Finance Integration** | | |
| --- | --- | --- | --- |
|  | **1** | **2** | **3** |
| Expert Judgment | Optimizing the use of  digital technology (ST.3) | Coordination and synergy  between stakeholders (ST.4) | Improving the quality of  Human Resources (ST.1) |
| Short Term Realistic Value | Improving the quality of Human Resources (ST.1) | Establishing real time information through database  integration and reporting (ST.2) | Optimizing the use of digital technology (ST.3) |
| Long Term Net Value | Improving the quality of Human Resources (ST.1) | Establishing real time information through database  integration and reporting (ST.2) | Optimizing the use of digital technology (ST.3) |

Source: Author’s compilation (2021)

The results show that the short and long-term strategies are in the same order. Improving the

***User***

*2021-12-07 16:51:34*

--------------------------------------------

What is the argument for each of the first and second statements, give a brief analysis or review.

quality of human resources, establishing real-time database integration and reporting, and

optimizing the use of digital technology, become the priority strategies. However, the expert

judgment put the optimization use of digital technology to be the priority strategy.

5. Discussion
   5.1 Priority Solution in developing a sustainable Islamic social finance integration model

***User***

*2021-12-07 16:52:46*

--------------------------------------------

Provide adequate arguments, in the financing program covered by sharia financing, success is determined by aspects of distribution, use or utilization (consumptive, productive economy), returns, and governance

Financial integration in providing financing to beneficiaries is a high-priority solution

that can be implemented in the short and long term. Further, this solution is very consistent with the sustainability paradigm, where the funding provided will broaden the range of benefits and, ultimately, create financial independence for the beneficiaries, as stated by Haneef et al. (2015), Nurlita & Ekawaty (2018), Taufiq et al. (2018), Azganin et al. (2021), Thaker et al. (2021), and Widiastuti et al. (2021). This also supports Hamed (2020) claim that financing benefits	small	businesses	by	maximizing	Islamic	social	financial	instruments (zakat, *infaq*, *sadaqah*, and waqf) as the best strategy for improving the recipients' quality of life. The financing provided through integrating Islamic social finance instruments has a cheap cost of the fund; thus, it ensures the beneficiaries' welfare. Furthermore, the integration of financing demonstrates that Islamic social fund instruments serve multiple purposes: consumptive zakat is used to provide emergency assistance in the short term, productive zakat and benevolence funds are used to support economic recovery in the medium term, and waqf or Sukuk are used to build long-term resilience. The financial integration of Islamic social fund instruments has also occurred in several institutions, though only on a micro-scale. Several institutions in Indonesia have worked to integrate zakat and waqf funds. Dompet Dhuafa, for example, integrated zakat and waqf funds when constructing an Integrated Health Home (Hospital). Cash Waqfs are used to build infrastructure on waqf land, whereas zakat funds provide free health care to the poor. Further, recently, Dompet Dhuafa, BAZNAS, and BWI have collaborated to integrate zakat and waqf, the newly established MoU, to collaborate on social projects.

This priority, however, differs from expert opinion, which emphasizes the importance of integrating nationally centralized data, such as database on donors, beneficiaries, financial reports, and so on. According to Afriadi & Sanrego (2016), Arwani (2020), and Hudaefi et al. (2021), the formation of national-centered data is critical for optimizing the integration and management of Islamic social finance. The integration of beneficiary-related information is

crucial for detecting data on beneficiary development and preventing overlapping distributions. Since Islamic social funds are still managed in a fragmented manner (partially), no single platform currently integrates data from all instruments of Islamic social finance in Indonesia. As the latest development, a data integration platform, has been established in the zakat sector. BAZNAS, an Indonesian zakat institution and regulator, released SIMBA (BAZNAS Information Management System), an integrated platform for zakat database management from all BAZNAS and LAZNAS across Indonesia. This platform only integrates data from national zakat collections. As a result, establishing a central platform capable of integrating data on all Islamic social finance instruments is critical.

Financial integration and integration of national data centers are interrelated. This is since funding distribution necessitates specific data to ensure the long-term viability of the funds provided. Data integration is needed to reduce the potential risk and realize the collection of distributed financings so that the supply of funds will be guaranteed. Further, the integration of beneficiary data is needed to ensure the depth of the reach of benefits and the creation of financial independence; thus, sustainability will be achieved. On another side, financial integration remains a short- and long-term priority because it is directly related to the primary goal of Islamic social finance, which is to create prosperity by improving the business and economic capacity of beneficiaries. As one of the priority solutions in this research, the

***User***

*2021-12-07 16:56:28*

--------------------------------------------

What are the operational steps to create a legal environment?

regulator must provide a legal environment to accommodate all changes and developments in

the management of Islamic Social Finance.

5.2 Strategic Priorities in developing a sustainable Islamic social finance integration model According to expert judgments, the strategy with the highest priority is optimizing the

use of digital technology. This goes in line with Saad et al. (2017), Thaker et al. (2018), Muneeza & Nadwi (2019), Usman et al. (2020), Berakon et al. (2021), Nor et al. (2021), and Widiastuti et al. (2021), all of which emphasize the importance of using technology in

***User***

*2021-12-07 16:57:38*

--------------------------------------------

Type of technology. technology level, and what technology transfer and adoption processes will be implemented

managing the instrument of Islamic social finance. This strategy is also connected to the immediate solution, integrating national data centers for all Islamic social and finance instruments. To become effective and efficient, data integration necessitates the development of an advanced web-based platform that all stakeholders can use, which necessitates the optimization of digital technology.

However, in terms of short-term and long-term priorities, optimizing the use of technology has slipped to third place. Experts consider improving the quality of Human Resources to be the most important aspect, both in the short and long term. The importance of enhancing the capability of human resources goes in line with Hidayatullah & Priantina (2018), Ali et al. (2020), Widiastuti et al*.* (2021). The difference in priorities between expert judgments and short/long term orientations occurs because technology investment, which is considered a long-term investment, necessitates a significant funding source, which some zakat institutions may not afford (Widiastuti et al., 2021). Optimizing the use of technology, which requires a large amount of investment, is a challenge for Islamic social finance institutions, particularly in Indonesia. This is due to the fact that the operational costs incurred by Islamic social fund institutions are determined by the amount of funds that can be raised. Meanwhile, there is a

significant gap between the realization and potential of Islamic social funds. As a result, institutions prefer to improve competencies and capabilities of human resources, relevant to their respective fields of work compared to improving technological features. This is also consistent with the aspects of BOCR addressed in this study. The cost of optimizing technology use is 0.0537, higher than the cost of improving the quality of human resources, which is around 0.0466. Costs associated with technology investments include acquiring digital channels, website management, social media, purchase of computers, maintenance cost, etc.

Furthermore, when determining priority strategies in the short and long term, experts also consider the risks exposed. The risk of optimizing the use of technology is high (0.0632), with the benefits obtained are also higher (0.0555). Meanwhile, the threat from the institution's strategy to improve the quality of human resources is lower at 0.0459. Cybercrime is one of the risks of optimizing the use of technology in Islamic social finance (Ninglasari & Muhammad, 2021). To mitigate this risk, competent human resources in their field are required. Implementing technology without qualified human resources will result in sub-optimal management. This is consistent with the findings of Widiastuti et al. (2021), who found a limited number of human resources capable of optimizing the use of advanced technology, particularly in those newly established institutions. Therefore, experts place qualified human resources as the prioritized strategy to mitigate risks, not only risks associated with technology application, but all risks associated with the integration of sustainable Islamic social finance.

Optimizing the use of technology and improving human resource quality can be done concurrently as stated by Widiastuti et al. (2021) that highlighted the importance of improving the quality of zakat management resources, particularly in the technological aspect, by emphasizing technology intensification (by developing *Amil*'s ability to use technology) and extensification (by increasing the number of *Amil* who master technology). Improving the quality of human resources is the foundation for the long-term integration of Islamic social finance, while technology facilitates optimal Islamic social finance management.

Finally, in order to integrate sustainable Islamic social finance, stakeholders must prioritize solutions and strategies, taking into account the benefits, opportunities, costs, and risks (BOCR) of each priority solution and strategy. Improving the quality of human resources and optimizing the use of digital technology are two strategies that must be implemented immediately. Based on short and long-term analysis and expert consensus, the two strategies are given the highest priority.

# 6. Managerial Relevancy

This study has several implications for stakeholders. The findings of this study emphasize solutions and priority strategies for integrating Islamic social finance, with Islamic social finance institutions playing a critical role in providing certification and training to

***User***

*2021-12-07 16:58:45*

--------------------------------------------

Aspects of human resources that must be improved are technical skills and managerial capabilities. Which of these two aspects is dominant depends on their respective positions.

improve the quality of human resources. Each training activity and accreditation must be given in accordance with the employee's field of work. Technology optimization, such as big data management, data security, e-marketing, and others, are examples of certification and training that must be provided.

The government, as a regulator, must assist Islamic philanthropic institutions in providing facilities or infrastructure to improve the quality of human resource managers, such as free training and certification for managers. Further, no law or regulation has required

Islamic Social Finance managers to have standardized or specific competencies. As a result, one form of input for the revision of the law and regulation requires all Islamic Social Finance managers to have specific competencies in managing Islamic philanthropic institutions so that these institutions can be managed more professionally.

This study also emphasizes the significance of financial integration and data integration on the national scale. Associations involved in Islamic philanthropy can use the research findings to establish strategic planning on integrating Islamic Social Finance instruments. From an academic standpoint, the results of this study develop previous studies on the integration of the Islamic Social Finance model, which were conducted without using specific mathematical methods. This study uses a systematic approach to create an ideal integration model that can be priorities both in the short and long term while also taking the aspect of BOCR into account.

# 7. Conclusion

This research uses ANP BOCR to develop priority solutions and strategies to integrate Islamic social finance based on expert agreement, short-term and long-term analysis. The aspect of human resources, regulation, and technological use become the highest aspect to be considered in achieving the research objective. Priority solutions in the short and long terms have the same order of priorities. Financial integration in providing funding to beneficiaries and integration with state fiscal policy is the first and second priority solution. Meanwhile, integration through non-formal coordination between institutions is the third priority solution in the long term. Besides, integrating national data centers has been the third priority solution for a long time and the highest solution based on expert judgments. In essence, the resulting priority solutions relate to aspects of financing, regulation, and technology.

As a strategy, improving the quality of Human Resources, establishing real-time information through database integration and reporting, and optimizing digital technology are three priority strategies that can be implemented in the short and long term. Furthermore, the experts mentioned the need for coordination and synergy between stakeholders in integrating sustainable Islamic social finance. To successfully incorporate Islamic social finance and ensure sustainability, stakeholders are encouraged to implement the research results (solution and strategy).

# 8. Limitation and Study Forward

This research only focuses on six aspects to develop ideal solutions and strategies: governance, sustainable financing, institutions, regulations, institutional human resources, and technology. However, this research has thoroughly examined each aspect's benefits, opportunities, costs, and risks. Future research can consider essential elements that have not been addressed in developing a sustainable Islamic social finance integration model, such as cultural aspects, donor behavior, government support, and others.

# Acknowledgment

This research grant is a Covid-19 Research Mandate with the contract number 1025/UN3.15/PT/2021. The authors would like to thank the Research and Community Service Institution (LPPM) of Universitas Airlangga for their financial assistance. The authors would like to thank Center Research and Publication (3P) of Faculty Economic and Business Universitas Airlangga as ethics committee. The authors would also like to thank all

***User***

*2021-12-07 17:01:53*

--------------------------------------------

Acknowledgments should also be conveyed to someone (individual) who played an important role in writing this article, but is not listed as a member of the writing team.

academicians, regulators, associations, practitioners, and community leaders for their support in writing this research.

# References

Adjar, H., Chakir, A., & Meskine, L. El. (2020). Exploratory Study of Islamic Social Finance in Morocco. *International Review of Entrepreneurial Finance*, *3*(2), 45–69. https://revues.imist.ma/index.php/IREF/article/view/25539

Afriadi, F., & Sanrego, yulizar d. (2016). Mengurai Permasalahan Distribusi Zakat Kepada Fakir Miskin: Pengalaman Organisasi Pengelola Zakat Baznaz, Dompet Dhuafa, Dan Lazizmu. *Jurnal Madania*, *20*(1), 23–36. https://doi.org/10.29300/madania.v20i1.83

Ahmad, M. (2019). An Empirical Study of The Challenges Facing Zakat and Waqf Institutions in Northern Nigeria. *ISRA International Journal of Islamic Finance*, *11*(2), 338–356. https://doi.org/10.1108/IJIF-04-2018-0044

Ahmed, B. O., Johari, F., & Wahab, K. A. (2017). Identifying the Poor and the Needy Among the Beneficiaries of Zakat Need for a Zakat-based Poverty Threshold in Nigeria. *International Journal of Social Economics*, *44*(4), 446–458. https://doi.org/10.1108/IJSE- 09-2015-0234

Ali, A. F. M., Rashid, Z. A., Johari, F., & Aziz, M. R. A. (2015). The effectiveness of Zakat in reducing poverty incident: An analysis in Kelantan, Malaysia. *Asian Social Science*, *11*(21), 355–367. https://doi.org/10.5539/ass.v11n21p355

Ali, M. M., Devi, A., Furqani, H., & Hamzah, H. (2020). Islamic Financial Inclusion Determinants in Indonesia: An ANP Approach. *International Journal of Islamic and Middle Eastern Finance and Management*, *13*(4), 727–747. https://doi.org/10.1108/IMEFM-01-2019-0007

Ambrose, A. H. A. A., Hassan, M. A. G., & Hanafi, H. (2018). A Proposed Model For Waqf Financing Public Goods and Mixed Public Goods in Malaysia. *International Journal of Islamic and Middle Eastern Finance and Management*, *11*(3), 395–415. https://doi.org/10.1108/IMEFM-01-2017-0001

Arumsari, V., & Rikawati, R. (2020). Islamic Social Funds: Enabling the Unable To Be Able. *Journal of Applied Business Administration*, *4*(1), 88–97. https://doi.org/10.30871/jaba.v4i1.1902

Arwani, A. (2020). Sharia Accounting on Indonesian Financial Accounting Standard on Zakat and Waqf Take on Industrial Revolution 4.0 and Society Era 5.0. *Journal of Islamic Accounting	and	Finance	Research*,	*2*(2),	229–258. https://doi.org/10.21580/jiafr.2020.2.2.6295

Ascarya. (2005). Analytic Network Process (ANP): Pendekatan Baru Studi Kualitatif. *Center for Central Banking Education and Studies, Bank Indonesia*.

Azganin, H., Kassim, S., & Auwal Adam, S. (2021). Proposed waqf crowdfunding models for small farmers and the required parameters for their application. *Islamic Economic Studies*. https://doi.org/10.1108/IES-01-2021-0006

Azha, L., Baharuddin, S., Sayurno, Salahuddin, S. S., Afandi, M. R., & H., H. A. (2013). The Practice and Management of Waqf Education in Malaysia. *Procedia - Social and Behavioral	Sciences*,	*90*(InCULT	2012),	22–30. https://doi.org/10.1016/j.sbspro.2013.07.061

Bassem, I., Ousman, J., Ansar, A., & Hamidar. (2019). Pathway of Welfare and Prosperity : Does Zakat Implementation Reach Indonesian To Economic Sustainability ? *Journal of Internet Banking and Commerce*, *24*(1), 1–13.

BAZNAS. (2021). *Outlook Zakat Indonesia 2021*. Pusat Kajian Strategis - Badan *Amil* Zakat Nasional (PUSKAS BAZNAS).

Beik, I. S. (2015). Towards International Standardization of Zakat System. *Fiqh Zakat International	Conference	2015*,	*November*,	3–17. https://doi.org/10.13140/RG.2.1.4490.1207

Berakon, I., Aji, H. M., & Hafizi, M. R. (2021). Impact of Digital Sharia Banking Systems on Cash-Waqf among Indonesian Muslim Youth. *Journal of Islamic Marketing*. https://doi.org/10.1108/JIMA-11-2020-0337

Bilo, C., & Machado, A. C. (2020). The Role of Zakat in the Provision of Social Protection: A Comparison between Jordan and Sudan. *International Journal of Sociology and Social Policy*, *40*(3–4), 236–248. https://doi.org/10.1108/IJSSP-11-2018-0218

Bin-Nashwan, S. A., Abdul-Jabbar, H., Aziz, S. A., & Haladu, A. (2020). Zakah compliance behavior among entrepreneurs: economic factors approach. *International Journal of Ethics and Systems*, *36*(2), 285–302. https://doi.org/10.1108/IJOES-09-2019-0145

Brau, J. C., Woller, G. M., Brau, J. C., & Woller, G. M. (2004). Microfinance: A comprehensive review of the existing literature. *Journal of Entrepreneurial Finance*, *9*(1), 1–28.

Caraka, R. E., Lee, Y., Kurniawan, R., Herliansyah, R., Kaban, P. A., Nasution, B. I., Gio, P. U., Chen, R. C., Toharudin, T., & Pardamean, B. (2020). Impact of COVID-19 large scale restriction on environment and economy in Indonesia. *Global Journal of Environmental Science	and	Management*,	*6*(Special	Issue),	65–84. https://doi.org/10.22034/GJESM.2019.06.SI.07

Chowdhury, M. S. R., Ghazali, M. F. bin, & Ibrahim, M. F. (2011). Economics of Cash WAQF management in Malaysia: A proposed Cash WAQF model for practitioners and future researchers. *African Journal of Business Management*, *5*(30), 12155–12163. https://doi.org/10.5897/ajbm11.1810

Cizakca, M. (1998). Awqaf in History and Its Implications for Modern Islamic Economies.

*Islamic Economic Studies*, *6*(1), 43–70. https://ssrn.com/abstract=3164811

Hamed, M. M. (2020). The Role of Islamic Social Finance in Mitigating Humanitarian Crises; A Multi-Range Strategy to Mitigate COVID-19 Impacts. *European Journal of Islamic Finance*, *0*(16), 1–10. https://doi.org/10.13135/2421-2172/4937

Haneef, Mohamed A, Huq, A., Mohammed, M. O., & Amin, F. B. (2013). Integration of Waqf and Islamic Microfinance for Poverty Reduction: A Survey in Kuala Selangor, Malaysia. *Journal of Islamic Finance*, *2*(2), 1–16.

Haneef, Mohamed Aslam, Pramanik, A. H., Mohammed, M. O., Bin Amin, M. F., & Muhammad, A. D. (2015). Integration of Waqf-Islamic Microfinance Model for Poverty Reduction: The Case of Bangladesh. *International Journal of Islamic and Middle Eastern Finance and Management*, *8*(2), 246–270. https://doi.org/10.1108/IMEFM-03-2014-0029

Hassanain, K. M. (2015). Integrating Zakah, Awqaf, and IMF for Poverty Allevation: Three Models of Islamic Micro Finance. *Journal of Economic and Social Thought*, *2*(2), 106–

120.	https://e-

resources.perpusnas.go.id:2350/docview/1873027697/10B524F6CCAD47DAPQ/5?acco untid=25704

Hidayatullah, A., & Priantina, A. (2018). Toward Zakat Management Integration in Indonesia: Problems and Solution. *Ahkam: Jurnal Ilmu Syariah*, *18*(2), 321–346. https://doi.org/10.15408/ajis.v18i2.6319

Hudaefi, F. A. (2020). How does Islamic fintech Promote the SDGs? Qualitative Evidence from Indonesia. *Qualitative Research in Financial Markets*, *12*(4), 353–366. https://doi.org/10.1108/QRFM-05-2019-0058

Hudaefi, F. A., Caraka, R. E., & Wahid, H. (2021). Zakat Administration in Times of COVID- 19 Pandemic in Indonesia: A Knowledge Discovery via Text Mining. *International Journal of Islamic and Middle Eastern Finance and Management*. https://doi.org/10.1108/IMEFM-05-2020-0250

Ibrahim, P., & Ghazali, R. (2014). Zakah as an Islamic Micro-Financing Mechanism to Productive Zakah Recipients. *Asian Economic and Financial Review, 4(1), 117-125.*, *4*(1), 117–125. <http://www.pakinsight.com/pdf-files/aefr> 4(1), 117-125.pdf

IRTI. (2014). Islamic Social Finance Report 2014. In *Islam and the Moral Economy*. https://doi.org/10.1017/cbo9780511617614.005

IRTI. (2020). *Islamic Social Finance Report 2020*.

Jermsittiparsert, K., & Sommanawat, K. (2019). The contributions of strategic Zakat in supply chain and profitability of the National Zakat Agency: A case study from National Zakat Agency Indonesia. *Humanities and Social Sciences Reviews*, *7*(2), 471–478. https://doi.org/10.18510/hssr.2019.7255

Jouti, A. T. (2019). An Integrated Approach for Building Sustainable Islamic Social Finance Ecosystems. *ISRA International Journal of Islamic Finance*, *11*(2), 246–266. https://doi.org/10.1108/IJIF-10-2018-0118

Kailani, N., & Slama, M. (2020). Accelerating Islamic Charities in Indonesia: Zakat, Sedekah and the Immediacy of Social Media. *South East Asia Research*, *28*(1), 70–86. https://doi.org/10.1080/0967828X.2019.1691939

Kamaruddin, M. I. H., & Auzair, S. M. (2019). Integrated Islamic Financial Accountability Model for Islamic Social Enterprise (ISE). *The Journal of Muamalat and Islamic Finance Research*, *16*(1), 17–36. https://doi.org/10.33102/jmifr.v16i1.206

Kasri, R. A. (2017). Management of Zakah Distribution: Empirical Evidence From Indonesia.

*AFEBI Islamic Finance and Economic Review (AIFER)*.

Khairi, K. F., Aziz, M. R. A., Laili, N. H., Nooh, M. N., Sbri, H., & Basah, M. Y. A. (2014).

Human and Economic Development through Share WAQF (WAQF Corporate): The Case of GLCS in Malaysia. *Life Science Journal*, *11*(x), 104–110. [http://www.lifesciencesite.com](http://www.lifesciencesite.com/)

Manurung, S. (2013). Islamic Religiosity and Development of Zakat Institution. *Qudus International Journal of Islamic Studies*, *1*(2), 197–220. https://doi.org/10.21043/QIJIS.V1I2.186

Ministry of Finance. (2020). APBN Kita: Kinerja dan Fakta. In *Kemenkeu.Go.Id* (Issue April). https:/[/www.kemenkeu.go.id/media/14243/](http://www.kemenkeu.go.id/media/14243/apbn-kita-januari-2020.pdf)a[pbn-kita-januari-2020.pdf](http://www.kemenkeu.go.id/media/14243/apbn-kita-januari-2020.pdf)

Ministry of Finance. (2021). Mengolah Potensi Ekonomi Syariah. *Majalah Media Keuangan*, *XIV*(166), 1–12.

Mohd Nor, S., Abdul-Majid, M., & Esrati, S. N. (2021). The role of blockchain technology in enhancing Islamic social finance: the case of Zakah management in Malaysia. *Foresight*, *23*(5), 509–527. https://doi.org/10.1108/FS-06-2020-0058

Mohd Thas Thaker, M. A., Mohd Thas Thaker, H., & Allah Pitchay, A. (2018). Modeling crowdfunders’ behavioral intention to adopt the crowdfunding-waqf model (CWM) in Malaysia: The theory of the technology acceptance model. *International Journal of Islamic and Middle Eastern Finance and Management*, *11*(2), 231–249. https://doi.org/10.1108/IMEFM-06-2017-0157

Muneeza, A., & Nadwi, S. (2019). The Potential of Application of Technology-Based Innovations for Zakat Administration in India. *International Journal of Zakat*, *4*(2), 87– 100. https://doi.org/10.37706/ijaz.v4i2.191

Mustofa, I., Santoso, D., & Rosmalinda, U. (2020). The Implementation of the Regulation of Cash Waqf Management in Higher Educational Institution in Indonesia and Malaysia (a Study of Legal System Theory). *Humanities & Social Sciences Reviews*, *8*(4), 69–77. https://doi.org/10.18510/hssr.2020.848

Ninglasari, S. Y., & Muhammad, M. (2021). Zakat Digitalization: Effectiveness of Zakat Management in the Covid-19 Pandemic Era. *Journal of Islamic Economic Laws*, *4*(1), 26– 44. https://doi.org/10.23917/jisel.v4i1.12442

Nurlita, E., & Ekawaty, M. (2018). The Direct and Indirect Effect of Zakat on the Household Consumption of Mustahik (A Study of Zakat Recipients from BAZNAS Probolinggo Municipality). *International Journal of Zakat*, *3*(2), 41–56. https://doi.org/10.37706/ijaz.v3i2.77

Pitchay, A. A., Thaker, M. A. M. T., Mydin, A. A., Azhar, Z., & Latiff, A. R. A. (2018). Cooperative-waqf Model: A Proposal to Develop Idle Waqf Lands in Malaysia. *ISRA International Journal of Islamic Finance*, *10*(2), 225–236. https://doi.org/10.1108/IJIF- 07-2017-0012

Raimi, L., Patel, A., & Adelopo, I. (2014). Corporate social responsibility, Waqf system and Zakat system as faith-based model for poverty reduction. *World Journal of Entrepreneurship, Management and Sustainable Development*, *10*(3), 228–242. https://doi.org/10.1108/wjemsd-09-2013-0052

Rosli, M. R. Bin, Salamon, H. Bin, & Huda, M. (2018). Distribution management of zakat

fund: Recommended proposal for asnaf riqab in Malaysia. *International Journal of Civil Engineering and Technology*, *9*(3), 56–64.

Saad, R. A. J., Idris, K. M., Shaari, H., Sawandi, N., & Derashid, C. (2017). Governance of non-profit organizations: A case of zakat institutions in Malaysia. *International Journal of Economic Research*, *14*(16), 253–265.

Saaty, T. L. (2013). Decision Making with the Analytic Network Process. In *International Series in Operations Research & Management Science* (pp. 1–40). Springer.

Saaty, T. L., & Vargas, L. G. (2006). Decision Making with the Analytic Network Process: Economic, Political, Social and Technological Application with Benefit, Opportunities, Cost and Risks. In *International Series in Operations Research and Management Science* (II, Vol. 195). Springer. https://doi.org/10.1007/978-1-4614-7279-7_15

Shalleh, M. C. M., Razali, S. S., Laksana, N. N. M., Embi, N. A. C., & Abdullah, N. I. (2020). Developing a sustainable model of Waqf-based Takaful for flood victims in Malaysia. *Journal of Islamic Accounting and Business Research*, *11*(9), 1941–1952. https://doi.org/10.1108/JIABR-10-2016-0114

Sukmana, R. (2020). Critical Assessment of Islamic Endowment Funds (Waqf) Literature: Lesson For Government and Future Directions. *Heliyon*, *6*(10), e05074. https://doi.org/10.1016/j.heliyon.2020.e05074

Sulistyowati. (2018). Designing Integrated Zakat-Waqf Models for Disaster Management.

*Journal of Islamic Monetary Economics and Finance*, *4*(2), 347–368.

Suryahadi, A., Izzati, R. Al, & Suryadarma, D. (2020). Estimating the Impact of Covid-19 on Poverty in Indonesia*. *Bulletin of Indonesian Economic Studies*, *0*(0), 175–192. https://doi.org/10.1080/00074918.2020.1779390

Taha, R., Adam, F., Ali, N. N. M., & Ariff, A. M. (2017). Religiosity and Transparency in the Management of Zakat Institutions. *Journal of Legal, Ethical and Regulatory Issues*, *20*(1), 1–9.

Taufiq, I. F., Kusnendi, K., & Nurasyiah, A. (2018). The Effect of Productive Zakat , Business Experience , and Mentoring on Farmers ’ Revenues ( Survey on Lumbung Desa Program by Sinergi Foundation in Cibaeud Village , Cigalontang District , Tasikmalaya Regency

). *International Journal of Zakat*, *3*(3), 55–67.

Thaker, M. A. M. T., Amin, M. F., Thaker, H. M. T., Khaliq, A., & Pitchay, A. A. (2021). Cash Waqf Model for Micro Enterprises’ Human Capital Development. *ISRA International Journal of Islamic Finance*, *13*(1), 66–83. https://doi.org/10.1108/ijif-08-2018-0091

Usman, H., Mulia, D., Chairy, C., & Widowati, N. (2020). Integrating Trust, religiosity and image into technology acceptance model: the case of the Islamic philanthropy in Indonesia. *Journal of Islamic Marketing*. https://doi.org/10.1108/JIMA-01-2020-0020

Von Pischke, J. D. (1996). Measuring the Trade-off Between Outreach and Sustain-ability of Microenterprise Lenders. *Journal of International Development*, *8*(2), 225–239. https://doi.org/10.1002/(SICI)1099-1328(199603)8:2<225::AID-JID370>3.0.CO;2-6

Widiastuti, T., Cahyono, E. F., Zulaikha, S., Mawardi, I., & Al Mustofa, M. U. (2021). Optimizing Zakat Governance in East Java using Analytical Network Process (ANP): The

Role of Zakat Technology (ZakaTech). *Journal of Islamic Accounting and Business Research*, *12*(3), 301–319. https://doi.org/10.1108/JIABR-09-2020-0307

World Bank. (2020). From Containment to Recovery. In *World Bank* (Issue October). https://openknowledge.worldbank.org/bitstream/handle/10986/34497/9781464816413.p df

Zammori, F., Bigliardi, B., & Carmignani, G. (2014). Selecting Inbound Logistic Policies: An ANP-based Multi Criteria Decision Making Approach. *International Journal of Logistics Systems and Management*, *19*(2), 212–244. https://doi.org/10.1504/IJLSM.2014.064659

Zauro, N. A., Saad, R. A. J., & Sawandi, N. (2020). Enhancing socio-economic justice and financial inclusion in Nigeria: The role of zakat, Sadaqah and Qardhul Hassan. *Journal of Islamic Accounting and Business Research*, *11*(3), 555–572. https://doi.org/10.1108/JIABR-11-2016-0134
