# Supplementary material for: Integrating sustainable Islamic social finance: An Analytical Network Process using the Benefit Opportunity Cost Risk (ANP BOCR) framework: The case of Indonesia
Source: PLoS One. 2022 May 26;17(5):e0269039. doi: 10.1371/journal.pone.0269039 (PMC9135187; doi:10.1371/journal.pone.0269039)
Supplement: Attachment — Submitted filename: Response to Reviewers.doc [file pone.0269039.s003.doc]

**Author’s Responses to the Reviewer Comments**

| Paper ID | PONE-D-21-31476 |
| --- | --- |
| TITLE | **Integrating Sustainable Islamic Social Finance: An Analytical Network Process using the Benefit Opportunity Cost Risk (ANP BOCR) Framework: The Case of Indonesia** |

| **No.** | **Comments from Reviewer (s)** | **Action** | **Page** | **Paragraph** |
| --- | --- | --- | --- | --- |
| 1. | Before the objective chapter, provide background on the problem related to the topic discussed because the goal is to answer the existing problems. It is necessary to briefly explain what is meant by a philanthropic institution and how does it relate to the topic of discussion? | Thank you for the advice.   a. We have added the problem in the abstract (purpose). b. Regarding "philanthropic institution", we replaced the word with the word "institutional aspect" because it is easier to understand the integration of Islamic social finance | 1 | Abstract |
| 2. | Explain how to determine the sample used by the author | Thank you for the comments   a. In the abstract section, we add the following sentence to explain how to determine the sample used   “The respondents were selected for their expertise and experience in the selected topic”   b. In method section, the purposive sampling technique aims to select respondents. There are three criteria to select respondent | 1  10 | Abstract  Method section, in “Data and research samples” paragraph 3 |
| 3. | Add to keyword: priority strategy | Thank you for the advice.  We have added those keywords. | 1 | Abstract |
| 4. | The author needs to describe the role of Islamic social finance in a broader perspective, not only in Muslim countries, or limited to the COVID-19 pandemic. The illustration can be expanded not only to the problem of the Covid-19 Pandemic (monetary crisis or global economic crisis, global food and financial crisis). | Thank you for your comment  .  We have revised this section by adding:  1. The role of Islamic social finance in general (before covid)  2. the role of Islamic social finance in several non-majority Muslim countries | 1 | Introduction paragraph 1 |
| 5. | The author needs to explain what are other supporting factors besides lack of optimal governance, that can be the reasons for the vast gap between the potential of ISF and the reality? Complete with another relevant literature review (LR), such as aspects of Islamic fundraising/finance, management, transparency, mutual trust, etc. | Thank you for your comments  We have added several factors that cause the vast gap between the potential of Islamic social finance.  Some of these factors are:  1. Lack of technology in the management  2. Lack of the ability and competence of the manager  3. Lack of synergy  4. Lack of trust and public literacy  We support these factors with various reputable journals. | 2-3 | Introduction paragraph 4 |
| 6. | First, explain what ISF governance is, then explain its aspects. | Thank you for the advice  We have added an explanation of ISF governance. | 3 | Introduction paragraph 5 |
| 7. | Please cite more fully and in detail regarding policies and regulations that do not support it, as well as what policy solutions can be offered? | Thank you for the comments  We've added explanations regarding unsupported policies, which include:   a. according to regulations, Islamic social finance instruments are still voluntary, so there is a gap in realization and potential for the collection b. there are no sanctions for those who do not pay zakat there are different regulations between Islamic social finance instruments | 3 | Introduction paragraph 6 |
| 8. | Provide an explanation of the current state of sustainability of ISF along with references from previous research. If there is no review on this matter, please provide a reference that strengthens your statement that most of the empowerment programs sourced from the ISF do not meet the sustainability aspect. | Thank you for the review  Regarding statement that strengthens sustainability of empowerment programs, we apologize that we could not find any journals that support this statement.  However, we wrote this statement based on the practice that occurred in the field and the results of the focus group discussions that we conducted with experts in Islamic social finances, especially zakat and waqf. | 3 | Introduction paragraph 8 |
| 9. | Add the challenge from the side of Muslims who oppose the ISF being regulated by the government because Indonesia is based on Pancasila, not a country with Islamic law, so its population consists of various beliefs and religions. There could be a sense of injustice if the government only regulates and takes money from Muslims to help government finances | Thank you for the advice  We have added the challenge to strengthen our introduction. | 3 | Introduction paragraph 8 |
| 10. | Literature Review for contemporary Islamic finance needs to be enriched especially from reputable global indexed primary journals, covering Islamic banking, Islamic insurance, etc. | Thank you for the advice  We have added reputable journals related to contemporary Islamic social finance. | 4 | Literature review, sub section “The concept of Islamic social finance and its instrument” paragraph 1 |
| 11. | Regarding sentences “Further, the Islamic social finance instruments are still partially Managed”, Is this sentence incomplete or imperfect? Correct this sentence so that it is easier to understand | Thank you for your review  We have revised this sentence | 6 | Section of Literature review, sub section “Issues with Islamic social finance integration”, paragraph 1 |
| 12. | Regarding sentences “Given the challenges which consequently create a significant gap between the potential and actualization of Islamic social finance management and accounting for the enormous potential of Islamic social finance, it is necessary to integrate Islamic Social Finance instruments on an ongoing basis to achieve optimum level of management.”, In Scientific Writing, avoid in one paragraph consisting of only one sentence | Thank you for your review  We have revised this sentence | 6 | Section of Literature review, sub section “Issues with Islamic social finance integration”, paragraph 1 |
| 13. | The importance of including Literature Review about integration aspects of growth, aspects of justice and aspects of sustainability | Thank you for the review.  We have revised this section as directed. We also include citations from reputable journals to support this revision. | 7 | Literature review sub section “Integration model of Islamic social finance” paragraph 1 |
| 14. | Mention what criteria are meant? | Thank you for the comments  Criteria that we mean is construct. In ANP, there is a construct to build the ANP model framework. Construct consists of six aspects, solutions, and strategies that we develop. | 7 | Method, sub section “research method and analysis approach” paragraph 1 |
| 15. | The author needs to explain some of the criteria used as input in this research using the two-level ANP method and why the BOCR analysis was selected | Thank you for the review  We have explained the reason we use two-level ANP (BOCR analysis) in the method.  We use two-level ANP (BOCR analysis) to analyze solutions and strategies that can be implemented in the short term and the most priority in integrating Islamic social finance by analyzing every benefit, opportunity, cost, and risk of each integration aspect. | 7-8 | Method, sub section “Research method and analysis approach” paragraph 1 line 8-13 |
| 16. | In sentences “literature review was conducted as part of this study to strengthen the ANP model that was Developed”, Mention the Literature Review focused on what aspects  ? | Thank you for your comments  The literature review in our method is used to strengthen the ANP model and collect problems, solutions, and strategies related to optimizing Islamic social finance.  We have added the explanation in the manuscript. | 7-8 | Method, sub section “Research method and analysis approach” paragraph 2 |
| 17. | In sentences “Human resources play a crucial role in Islamic Social Finance management”, What is the important role in this case? Give a brief explanation | Thank you for the advice  We have added explanations regarding the urgency and definition of each aspect we analyze | 11 | Result sub section “Priority aspect” paragraph 2 |
| 18. | What institutional aspects are the focus of this article (structure, function/role, coordination system, business partnerships with other parties). | Thank you for the review  We focus on the structure and coordination system. And we have added the explanation in our manuscript. | 11-12 | Result, sub section “Priority aspect” paragraph 2 |
| 19. | About integration between Islamic social finance institutions based on capacity development and division of roles, what types of integration are meant (horizontal integration, vertical integration or horizontal coordination, vertical coordination)? Please explain | Thank you for your comments  The integration is done vertically and horizontally. We have added it to our manuscript. | 12 | Result, sub section “Priority solution in integrating sustainable Islamic social finance” paragraph 1 |
| 20. | Explain what is the difference between short-term and long-term priority solutions shown in table 4 based on the expert agreement? | Thank you for the advice.  We have added an explanation of the differences between short-term and long-term priority solutions in our manuscript.  Short term solution considers the marginal cost of the benefits obtained. On the contrary, in the long term, the priority is achieved when the total number of benefits and opportunities are obtained after reducing the total cost and risk (Saaty & Vargas, 2006; Zammori et al., 2014).  On the other hand, an expert judgment does not consider the benefits, opportunity, cost, and risk aspects of each solution formulated in this study. | 13 | Result, sub section “Priority solution in integrating sustainable Islamic social finance” paragraph 2 |
| 21. | Regarding sentences “According to the data processing results, the first and second priorities in the short and long- term are in the same order. The solutions that will be implemented first and foremost in the short and long term are financial integration in providing funding to beneficiaries and integration of Islamic social finance with state fiscal policy. According to expert judgment, integrating national data centers is the highest priority solution, with financial integration in providing funding to beneficiaries coming in second.”, It is necessary to conduct a more in-depth analysis and based on this analysis, it is synthesized by the author | Thank you for your comment  We provide arguments in the discussion section | 15 | Discussion, sub section “Priority solution in developing a sustainable Islamic social finance integration model” |
| 22. | Regarding sentences “cryptocurrency waqf”, What is the equivalent in Syar'i or Arabic? | Thank you for the comments  We reviewed the cryptocurrency waqf and its relevance to Sharia. Then, we decided not to include this type of waqf in our manuscript. | 15 | Result sub section “Priority strategies in integrating sustainable Islamic social finance” paragraph 1 |
| 23. | Regarding sentences “The order of priority strategies is shown in table 6, which displays short-term, long-term priority solutions and expert judgment. The strategies are summarized in table 7 as follows”, Give a brief description before referring to tables 6 and 7. In scientific writing, avoid one paragraph consisting of only one sentence. | Thank you for the advice  We've revised this sentence by giving a brief description | 14 | Result, sub section “Priority strategies in integrating sustainable Islamic social finance”, paragraph 2 |
| 24. | Regarding sentences “The results show that the short and long-term strategies are in the same order. Improving the quality of human resources, establishing real-time database integration and reporting, and optimizing the use of digital technology, become the priority strategies. However, the expert judgment put the optimization use of digital technology to be the priority strategy.”, What is the argument for each of the first and second statements, give a brief analysis or review. | Thank you for your comment  We provide arguments for the first and second statements in the discussion section   a. Optimizing the use of digital technology b. Improving the quality of human resource | 17  17  17 | Discussion, sub section “strategic solution in developing a sustainable Islamic social finance integration model”  Paragraph 1  Paragraph 2-3 |
| 25. | Provide adequate arguments, in the financing program covered by sharia financing, success is determined by aspects of distribution, use or utilization (consumptive, productive economy), returns, and governance | Thank you for the advice  We have added an adequate argument about program financing.  We add arguments that include:   1. Relation of financing integration with existing integration theory 2. Reputable journals which shows the importance of distribution in zakat management 3. Reputable journals which shows that financing from Islamic social, financial instruments has a positive impact on the welfare of the poor | 15 | Discussion, sub section “Priority solution in developing a sustainable Islamic social finance integration model” paragraph 1 |
| 26. | What are the operational steps to create a legal environment? | Thank you for the comments  In our opinion, there are several steps to create a legal environment which at least includes: government must revise the regulation, the government must socialize the revised laws and regulations to all stakeholders, encourage Islamic social finance management institutions to implement the law by offering rewards or incentives that the Institution can use, such as free training and accompaniment. | 17 | Discussion sub section “Priority solution in developing a sustainable Islamic social finance integration model” paragraph 3 |
| 27. | Type of technology, technology level, and what technology transfer and adoption processes will be implemented in managing the instrument of ISF | Thank you for the comments  We added an explanation regarding the type of technology.  There are at least four types of technology that must be implemented in the integration of Islamic social finance, which consist of:   a. technology for creating a centralized database linked to the government and Islamic social finance supervisors b. technological advancements in supervision and monitoring c. technology in the marketing component to aid in the collection of target achievement d. technology to generate real-time management reports | 17 | Discussion, sub section “Strategic priorities in developing a sustainable Islamic Social finance integration model” paragraph 1 |
| 28. | Aspects of human resources that must be improved are technical skills and managerial capabilities. Which of these two aspects is dominant depends on their respective position. | Thank you for the comment.  We think that both skills (technical and management) are equally important. Technical skills are essential for staff to do administrative work and empowerment.  Meanwhile, management skills are needed by leaders or managers in making strategic ISF management decisions. | 18 | Managerial relevancy paragraph 1 |
| 29. | Acknowledgments should also be conveyed to someone (individual) who played an important role in writing this article, but is not listed as a member of the writing team. | Thank you for the comments  We did not name anyone involved in this study. However, we mention all representatives of academics, regulators, associations, and community leaders involved in this research for their support in the FGDs, in-depth interviews, and filling out our questionnaires. | 20 | Acknowledgement paragraph 1 |
